# Targeted Dual‐Responsive Liposomes Co‐Deliver Jolkinolide B and Ce6 to Synergistically Enhance the Photodynamic/Immunotherapy Efficacy in Gastric Cancer through the PANoptosis Pathway

**DOI:** 10.1002/advs.202502289

**Published:** 2025-05-19

**Authors:** Chenhui Ma, Lei Gao, Kewei Song, Baohong Gu, Bofang Wang, Yang Yu, Xueyan Wang, Xuemei Li, Jike Hu, Weigao Pu, Yunpeng Wang, Na Wang, Dedai Lu, Zhijian Han, Hao Chen

**Affiliations:** ^1^ The Second Clinical Medical College Lanzhou University Lanzhou 730000 China; ^2^ Department of Thyroid Surgery Second Affiliated Hospital Zhejiang University School of Medicine Hangzhou 310009 China; ^3^ Gansu Provincial Key Laboratory of Environmental Oncology Lanzhou Gansu 730030 China; ^4^ National Institute for Data Science in Health and Medicine Xiamen University Xiamen 361102 China; ^5^ Key Laboratory of Eco‐Functional Polymer Materials of the Ministry of Education Northwest Normal University Lanzhou 730070 China; ^6^ Department of Tumor Surgery Lanzhou University Second Hospital Lanzhou 730030 China; ^7^ Shanghai General Hospital Shanghai Jiao Tong University School of Medicine Shanghai 201620 China

**Keywords:** gastric cancer, immunotherapy, liposomes, PANoptosis, photodynamic therapy

## Abstract

Improving the efficacy of gastric cancer (GC) treatment remains an ongoing challenge. Considering the increasing importance of PANoptosis, a novel form of programmed cell death, the current study integrates photodynamic therapy (PDT) and chemodynamic therapy (CDT) into nanoliposomes. This approach utilizes the ability of photosensitizer Chlorin e6 (Ce6) to generate reactive oxygen species (ROS) and the function of the natural targeting agent Jolkinolide B to activate the PANoptosis molecular switch, inducing the ROS‐caspase8/PANoptosis pathway to promote GC cell death. The designed CJP–TiN liposome targets GC via internalizing RGD peptide (iRGD), and demonstrates ROS/pH dual responsiveness in the tumor microenvironment. In vitro and in vivo experiments show effective ROS generation ability under light exposure, killing tumor cells and triggers thioether bond cleavage for dual‐controlled drug release. The combined therapy enhances antitumor effect, converting “cold tumors” into “hot tumors,” thereby enhancing the success of immunotherapy. The role of CJP–TiN as a PANoptosis inducer in the tumor microenvironment is confirmed, thereby expanding its application potential as a molecularly targeted therapy for GC treatment, and providing a novel perspective for therapeutic strategies.

## Graphical abstract

Schematic model of CJP–TiN liposomes for gastric cancer (GC) treatment. Internalizing RGD peptide (iRGD) targets tumor cells, while Chlorin e6 (Ce6) generates reactive oxygen species (ROS). Jolkinolide B (JB) activates the PANoptosis pathway via caspase‐8, enhancing GC cell death and immune response, transforming “cold tumors” into “hot tumors” by promoting M1 macrophage and T‐cell infiltration.

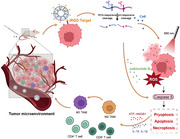

## 1 Introduction

Gastric cancer (GC) is a prevalent malignancy of the digestive system, with advanced‐stage patients experiencing a 5‐year survival rate of merely 10.4%.^[^ ^1^ ^]^Although surgery is the primary treatment approach, advanced gastric cancer still presents significant challenges.^[^ ^2^ ^]^ Currently, R0 resection can be achieved in only 40–50% of patients suffering from the advanced disease, and even after successful resection, 50–90% of these patients will relapse or die, resulting in an overall 5‐year survival rate of < 30%.^[^ ^3^ ^]^ Current treatments for advanced or recurrent GC include chemotherapy, radiotherapy, molecularly targeted therapy, and immunotherapy, with immunotherapy being recommended as both a first‐ and second‐line treatment option.^[^ ^4^ ^]^ Although immunotherapy has led to increased survival rates, many patients still fail to benefit.^[^ ^5^ ^]^ It is therefore necessary to explore more efficient and less harmful treatment options to significantly improve patient prognosis.

Photodynamic therapy (PDT) is a promising cancer treatment approach that exerts tumoricidal effects by generating reactive oxygen species (ROS).^[^ ^6^ ^]^ Due to its spatiotemporal control and minimal side effects, PDT can induce immunogenic cell death, triggering a systemic immune response that suppresses both primary and metastatic tumors.^[^ ^7^ ^]^ PDT has shown promising results in the treatment of cervical cancer,^[^ ^8^ ^]^ skin cancer,^[^ ^9^ ^]^ and digestive system cancers,^[^ ^10^ ^]^ and is gradually becoming an important adjunct to surgery, radiotherapy, and chemotherapy. However, the broader application of PDT is still limited by challenges in improving its overall treatment efficacy. Despite the critical role of photosensitizers in PDT, current options face several drawbacks, including poor water solubility, limited photostability, and low selectivity, all of which hinder their clinical use.^[^ ^11^ ^]^ To address these issues, drug delivery vehicles, such as liposomes, can be considered. More specifically, liposomes are nanocarriers that can carry hydrophilic drugs in their internal aqueous phases and incorporate hydrophobic drugs into their phospholipid bilayers. As drug delivery vehicles that are approved by the United States food and drug administration, liposomes can optimize the pharmacokinetics of photosensitizers, reduce the degrees of systemic and off‐target toxicity, extend circulation times, and achieve targeted delivery;^[^ ^12^ ^]^ consequently, they can offer a potential solution to enhancing the efficacy of PDT.

In recent years, the combination of PDT with other treatment approaches has drawn considerable attention in the medical field.^[^ ^13^ ^]^ Indeed, our group previously demonstrated that the combined application of PDT with chemotherapy, targeted therapy, and immunotherapy achieved significant efficacies in patients with advanced GC.^[^ ^14^, ^15^ ^]^ In addition, PANoptosis, which was proposed by Malireddi et al. in 2019, is a new form of programmed cell death that combines pyroptosis, apoptosis, and necroptosis.^[^ ^16^ ^]^ Our preliminary work in this area indicated that the natural product Jolkinolide B (JB) exhibits a significant anti‐GC activity, and its primary mechanism of action involves binding to and activating caspase‐8 as a molecular targeted agent, thereby initiating the PANoptosis molecular switch.^[^ ^17^ ^]^ In another study, Lin et al.^[^ ^18^ ^]^ found that an NFS1 deficiency can induce PANoptosis and promote synergy with oxaliplatin treatment, wherein the increased ROS levels activated caspase‐8 and its downstream pathways.

In GC immunotherapy, commonly used anti‐PD‐1/PD‐L1 drugs include nivolumab and pembrolizumab. Previous research has indicated that 12–65% of GC patients exhibit upregulated PD‐L1 expression, which is closely linked to a poor prognosis.^[^ ^19^, ^20^ ^]^ As a “cold tumor” with a poor immunogenicity, GC renders it difficult for immune checkpoint inhibitors to effectively block immune escape, resulting in a limited efficacy for immune checkpoint blockade therapy.^[^ ^21^, ^22^ ^]^ Therefore, there is an urgent requirement to the development of a comprehensive therapeutic strategy that can both kill tumors and convert “cold tumors” into “hot tumors.”

**Scheme 1:**
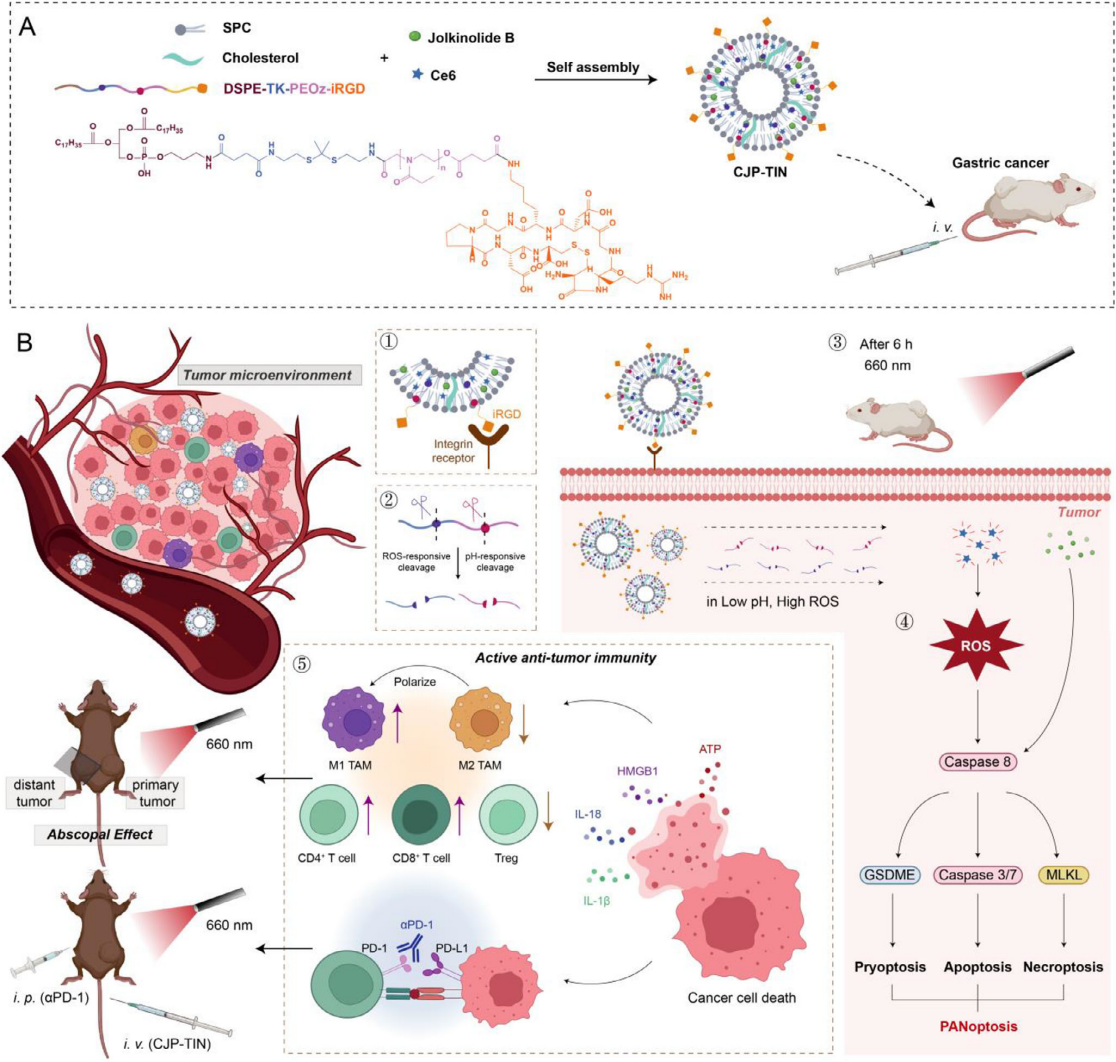
Nanoparticle CJP‐TiN enhances GC treatment efficacy by delivering Jolkinolide B and Ce6. A) Liposomal nanoparticles were prepared using a self‐assembly thin‐film hydration method. B) Ce6 was activated in vitro to generate ROS, which, together with Jolkinolide B, activated the PANoptosis molecular switch caspase 8, reshaping the immune microenvironment to improve photodynamic and immunotherapy efficacy.

Thus, we herein report the design of a functionalized liposome (CJP–TiN) for the treatment of GC (**Scheme** **1**). This liposome combines the ROS responsiveness of the thioether bond (TK), the pH responsiveness of poly(2‑ethyl‑2‑oxazoline) (PEOz), and the specific binding of internalizing RGD peptide (iRGD) to α_v_β_3_,^[^ ^23^, ^24^, ^25^ ^]^ by encapsulating hydrophobic Chlorin e6 (Ce6) and JB via the thin‐film hydration method, with the novel synthesis of DSPE‐TK‐PEOz_2k_‐iRGD for the first time. It is expected that the activation of CJP–TiN under light irradiation will induce GC cell death via the ROS‐caspase8/PANoptosis signaling pathway. This combined therapeutic approach should overcome the limitations of single therapies by targeting tumor cells through multiple pathways, providing a new perspective for GC treatment. In principle, the aim of this study is to improve overall GC treatment efficacy by combining PDT with enhanced photosensitizing materials.

## 2 Results

### 2.1 Target Validation and Characterization of CJP–TiN

**Figure 1:**
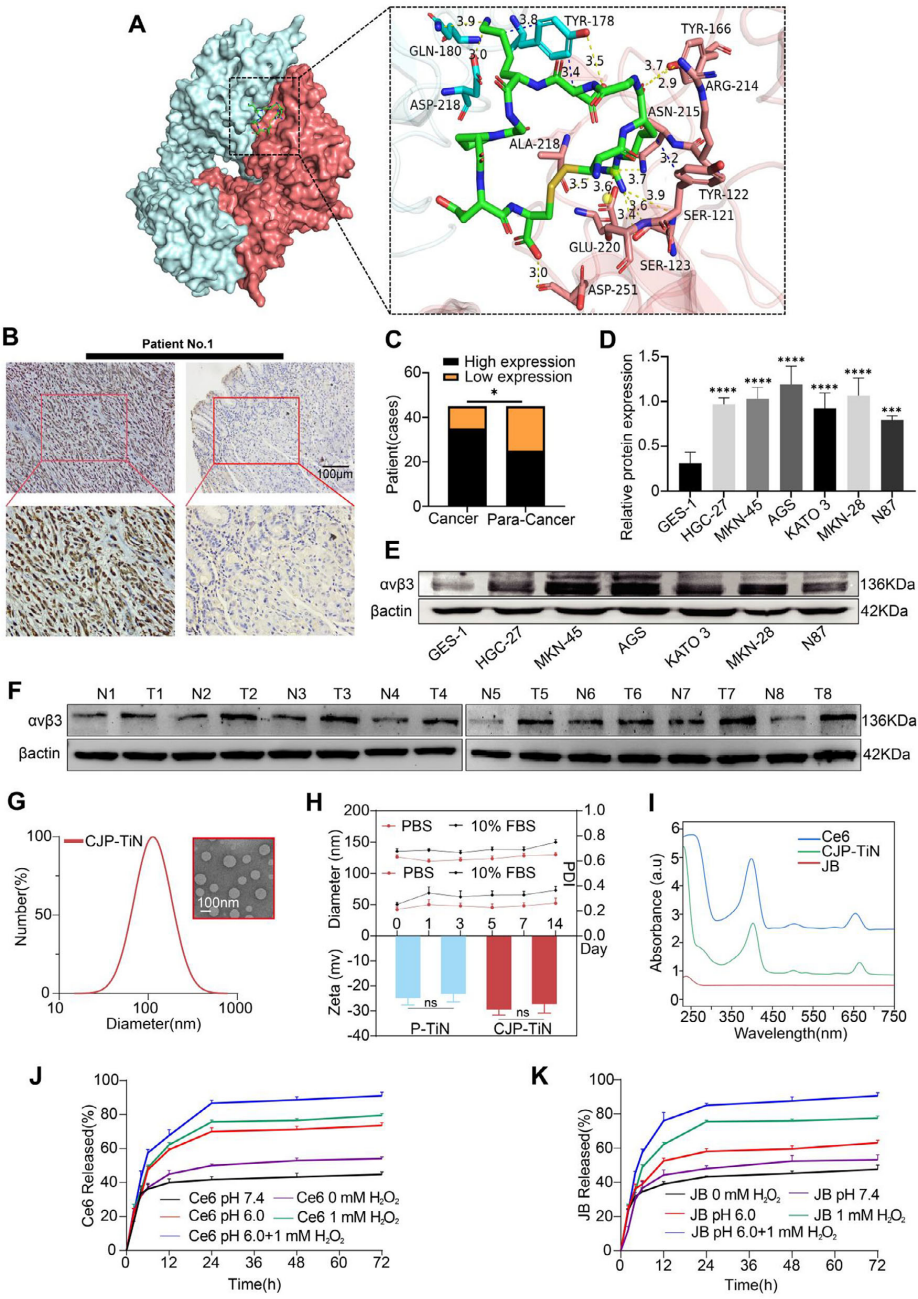
Target validation and characterization of CJP–TiN. A) The molecular docking between the CJP–TiN ligand (iRGD) and the GC cell membrane surface receptor (α_v_β_3_) revealed a binding energy of −8.2 kcal m ^−1^. B) Staining of human GC and adjacent non‐cancerous tissues showed high expression of α_v_β_3_ in gastric cancer tissues, with scale bars of 100 µm. C) α_v_β_3_ IHC scoring showed significantly higher expression in cancer tissues compared to para‐cancer tissues (*n* = 45). ^*^ *p* < 0.05. D,E) Expression and quantitative analysis of α_v_β_3_ in GC cell lines and human gastric epithelial cells GES‐1 (*n* = 3). ^***^ *p* < 0.001, ^****^ *p* < 0.0001. F) Expression of α_v_β_3_ in cancerous and adjacent tissues from eight GC patients (*n* = 8). G) Particle size distribution and TEM characterization of CJP–TiN. Scale bar: 100 nm. H) Stability evaluation of drug‐loaded CJP–TiN. Hydrodynamic diameter, polydispersity index (PDI), and zeta potential were measured following incubation in PBS and cell culture medium (10% FBS) at 37 °C for up to 14 days (*n* = 3). ns indicates no statistical significance. I) UV‐vis absorption spectra of Ce6 (blue), JB (red), and CJP–TiN (green) in aqueous solution. J,K) Release rates of Ce6 and JB from CJP–TiN under normal pH and simulated tumor microenvironment conditions.

To evaluate the feasibility of nanoparticle targeting, molecular docking techniques were employed to analyze the binding mode of iRGD with α_v_β_3_ (**Figure** **1**A), providing information regarding the binding energy, interaction types, and bond lengths (Table S1, Supporting Information). The obtained results indicated that the binding energy of iRGD with α_v_β_3_ was −8.2 kcal m ^−1^, and it was deduced that iRGD exhibited van der Waals interactions with the Thr 212, Tyr 178, Gln 180, and Phe 177 residues of α_v_β_3_. Furthermore, it was found that the active site of α_v_β_3_ includes Ser, Tyr, Gln, Arg, and Asp residues (Figure S1, Supporting Information). Furthermore, the interaction between α_v_β_3_ and iRGD was further validated by surface plasmon resonance (SPR) analysis and alanine mutation analysis, as detailed in Figure S2 and Tables S2,S3 (Supporting Information). To validate the differences in target expression between normal and cancerous cells, immunohistochemical (IHC) staining was performed on 45 pairs of patient‐derived GC and their adjacent non‐cancerous tissues to identify α_v_β_3_ (Figure 1B). The obtained results showed that expression levels were significantly higher in the cancerous tissues than in the adjacent non‐cancerous tissues (*p* < 0.05) (Figure 1C). In addition, Western blot (WB) analysis was performed on eight pairs of GC tissues and their adjacent non‐cancerous tissues, demonstrating that α_v_β_3_ expression was significantly higher in the GC tissues (Figure 1F). To support these results, α_v_β_3_ expression was analyzed across multiple human GC cell lines (i.e., HGC‐27, MKN45, AGS, KATO 3, MKN‐28, and N87) and one normal gastric epithelial cell line (GES‐1). Notably, the expression levels were especially pronounced in the AGS, MKN45, KATO 3, and MKN‐28 cell lines (^****^ *p* < 0.0001) (Figure 1E,D).

The synthesis process of DSPE‐TK‐PEOz_2k_‐iRGD is shown in Figure S3 (Supporting Information). To determine the structural characteristics of DSPE‐TK‐PEOz_2k_‐iRGD, we used nuclear magnetic resonance (NMR) spectroscopy (Figure S4, Supporting Information). In addition, the structure was further validated by Fourier transform infrared (FTIR) spectroscopy and MALDI‐TOF mass spectrometry (Figures S5,S6, Supporting Information). To ensure the maximum encapsulation efficiency and drug loading of CJP–TiN, the material/drug mass ratio was optimized. Based on the IC_50_ (half‐maximal inhibitory concentration) values of Ce6 and JB in AGS and MKN45 cells (Figure S7, Supporting Information), 1:1 ratios were selected, achieving a maximum encapsulation efficiency and drug loading when the drug/material ratio was 10% (Table S4, Supporting Information). Consequently, this ratio was used for subsequent experiments. Transmission electron microscopy (TEM) observations revealed that CJP–TiN was spherical (Figure 1G), and while dynamic light scattering (DLS) analysis indicated a good monodispersity, with a particle size of 109.36 ± 3.2 nm, a polydispersity index (PDI) of 0.228 ± 0.003 (Table S5, Supporting Information), a zeta potential of −31.58 ± 1.73 mV, and a good stability over 14 d (Figure 1H). Absorption spectroscopy (Figure 1I) showed an intense signal corresponding to the characteristic absorption peak of Ce6 (398, 503, or 660 nm, blue curve), indicating the successful loading of Ce6 into CJP–TiN, and confirming effective encapsulation by the CJP–TiN composite. Moreover, the Ce6 and JB release properties were found to be significantly enhanced in an acidic environment (pH 6.0) and in the presence of hydrogen peroxide (1 mm H_2_O_2_), with a combination of these conditions leading to > 80% release within 72 h (Figure 1J,K). These findings suggest that the release of Ce6 and JB is modulated by the environmental acidity and oxidative stress, confirming that CJP–TiN exhibits pH/ROS‐responsive properties.

### 2.2 In Vitro Evaluation of the Nanoparticles

**Figure 2:**
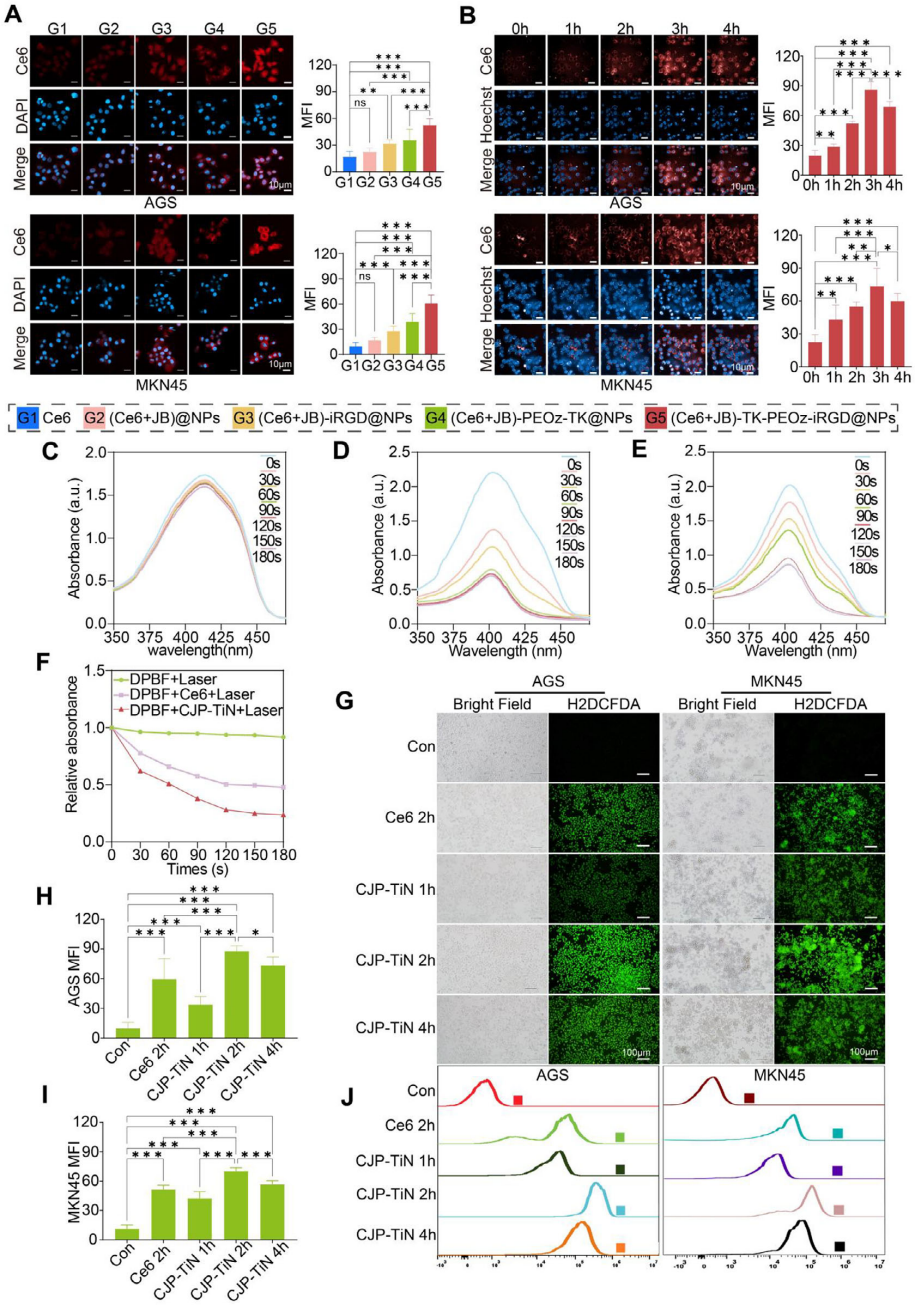
In vitro characterization of the nanoparticles. A) Uptake rates of different nanoparticles by AGS and MKN45 cells, as observed using CLSM: red (Ce6), blue (DAPI). B) Images captured using high‐content cellular imaging system after the co‐incubation of CJP–TiN with MKN45 and AGS cells for 4 h (*n* = 3). ^*^ *p* < 0.05, ^**^ *p* < 0.01, ^***^ *p* < 0.001. ns indicates no statistical significance. Scale bar: 10 µm. C–E) UV spectra of DPBF, Ce6+DPBF, and CJP‐TiN+DPBF after laser irradiation. F) Changes in the relative absorbance of DPBF over time for the different treatment groups. G–I) ROS levels, detected and quantitatively analyzed using H2DCFDA staining (*n* = 3). ^*^ *p* < 0.05, ^***^ *p* < 0.001. Scale bar: 100 µm. J) Flow cytometry detection and analysis of the cell ROS levels.

Confocal laser scanning microscopy (CLSM) was used to visualize the uptake of various materials by both AGS and MKN45 cells (**Figure** **2**A). The findings showed that Ce6 (red) was significantly distributed across all groups, particularly in the (Ce6+JB)‐TK‐PEOz‐iRGD@NPs group (CJP–TiN), where the intracellular red fluorescence was stronger. This indicated that iRGD modification and the pH/ROS‐responsive nature of the system led to significantly improved intracellular drug uptake. The results of high‐content cellular imaging analysis also demonstrated a time‐dependent uptake of Ce6 by CJP–TiN in AGS and MKN45 cells, peaking at 3 h (Figure 2B).

1,3‐Diphenylisobenzofuran (DPBF) is a commonly used chemical probe for detecting the generation of singlet oxygen (^1^O_2_). As shown in Figures 2C–E, after subjecting CJP–TiN to 660 nm laser irradiation, ¹O_2_ was rapidly generated, indicated by a significant decrease in the UV absorption peak of DPBF at 415 nm (0–180 s). The absorbance of DPBF significantly decreased in the Ce6+L (Figure 2D) and CJP–TiN+L (Figure 2E) groups, with the CJP–TiN+L group exhibiting a higher ¹O_2_ generation efficiency (Figure 2F), thereby demonstrating the high photosensitivity of CJP–TiN. To further verify the ROS release efficiency of CJP–TiN in cells, H2DCFDA fluorescence staining (green) was employed. As presented in Figures 2G, both the Ce6 and CJP–TiN treatment groups produced significant amounts of ROS, with the fluorescence intensity significantly increasing with a prolonged incubation time (especially in the CJP–TiN group after 2 h), reaching the highest levels of intracellular ROS. The flow cytometry (FCM) results also supported this conclusion Figures 2J, thereby indicating that CJP–TiN can effectively induce ROS generation under 660 nm excitation, thereby enhancing the efficacy of PDT.

### 2.3 TEM Images of CJP–TiN Cellular Uptake and Cell Viability Assessment

**Figure 3:**
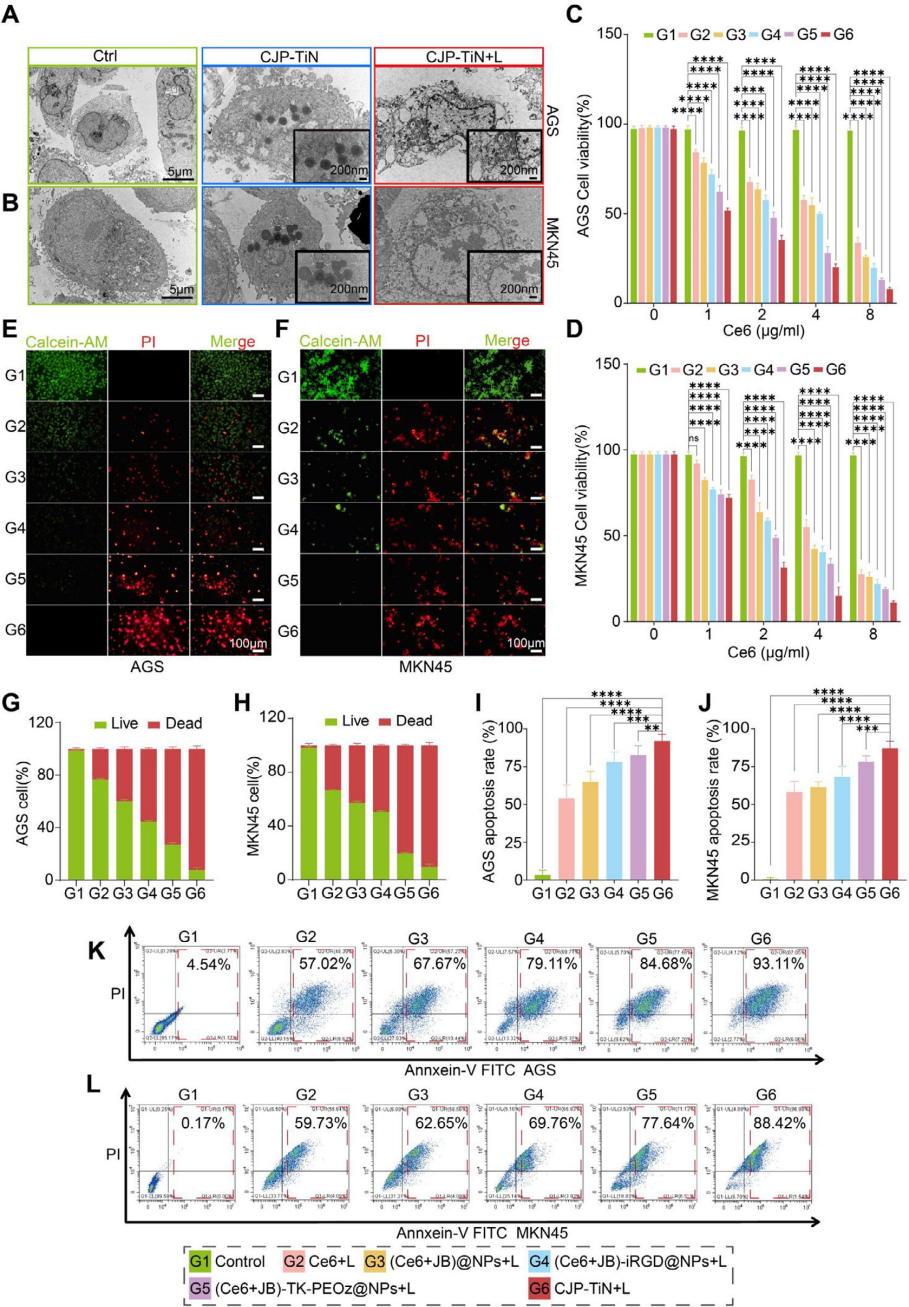
TEM images of CJP–TiN uptake and cell viability assessments. A,B) TEM images of the AGS and MKN45 cells showing CJP–TiN uptake and post‐intervention cell morphology (left scale bar: 5 µm, right scale bar: 200 nm). C,D) Cell viability assays of AGS and MKN45 for the different treatment groups and nanoparticle concentrations (*n* = 3). ^****^ *p* < 0.0001. ns indicates no statistical significance. E–H) Live/dead staining of the AGS and MKN45 cells cultured for 24 h with the different treatment groups (*n* = 3). Scale bar: 100 µm. I–L) Use of FCM to detect apoptosis and analyze the AGS and MKN45 cells with the different treatment groups (*n* = 3). ^**^ *p* < 0.01, ^***^ *p* < 0.001, ^****^ *p* < 0.0001.

The PDT potential of CJP–TiN nanoparticles was assessed in AGS and MKN45 GC cells through multiple experimental approaches. As can be seen from the TEM images presented in **Figure** **3**A,B, the CJP–TiN nanoparticles were effectively internalized into both types of cells, resulting in membrane rupture, organelle breakdown, and cellular structure disappearance following laser exposure (CJP–TiN+L group). The increased size of intracellular liposomes may result from osmotic swelling, but this does not affect the interpretation of cellular uptake behavior. In addition, the cell viability tests (Figure 3C,D) indicated that both the Ce6 and CJP–TiN+L groups displayed notable dose‐dependent cytotoxicities against the AGS and MKN45 cells, with the more pronounced effect observed for the CJP–TiN+L group suggesting that CJP–TiN and PDT exhibit a synergistic cytotoxic effect in cancer cells. The results obtained from the Calcein‐AM/propidium iodide (PI) dual staining experiment (Figure 3E,F) revealed that the CJP–TiN+L group exhibited a markedly decreased cell viability and a significantly increased rate of cell death. Annexin V‐FITC/PI flow cytometry (Figure 3K,L) further confirmed that CJP–TiN significantly induced apoptosis, with this group exhibiting the highest rate of apoptosis.

### 2.4 RNA Sequencing and In Vitro Validation after CJP–TiN Intervention

**Figure 4:**
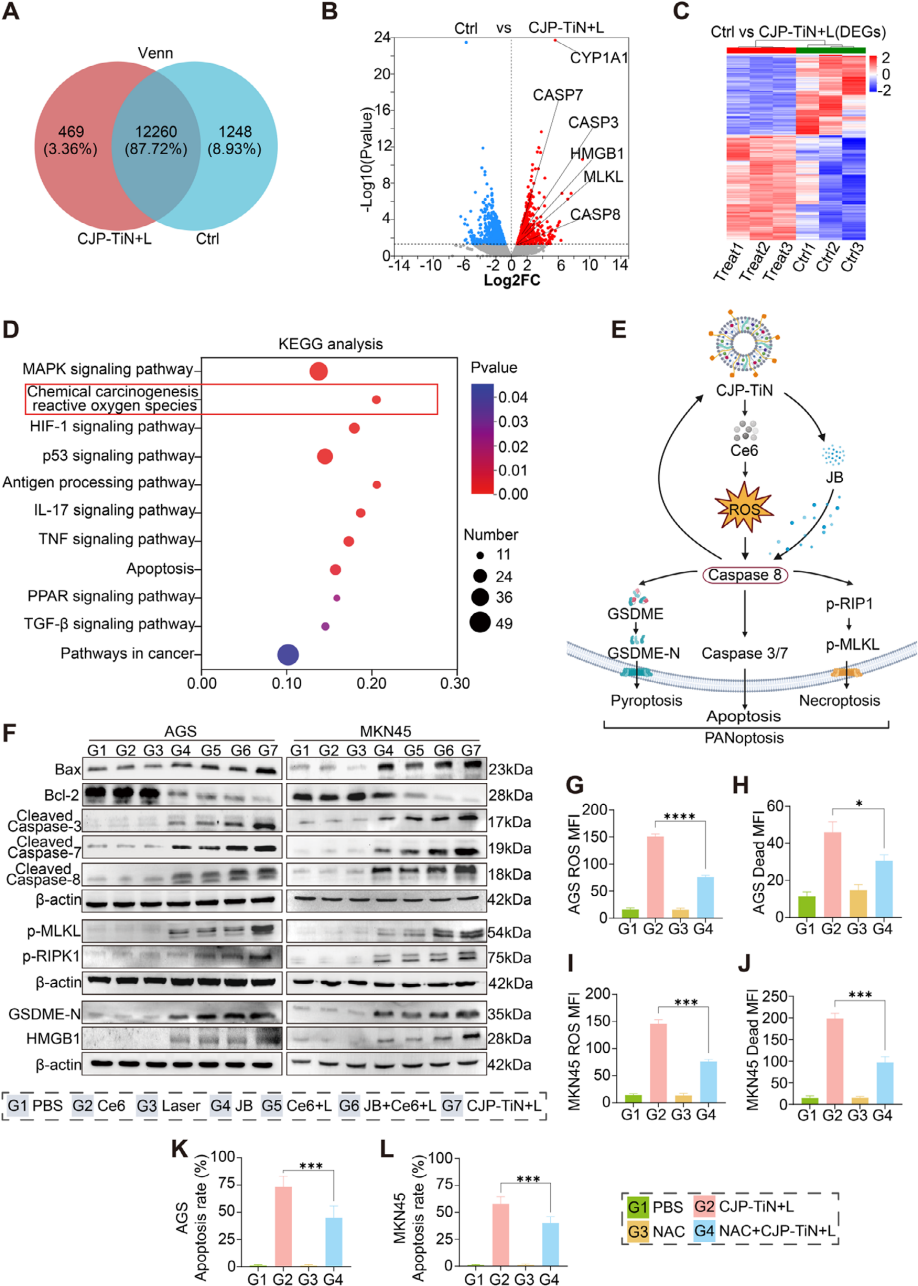
RNA sequencing and in vitro validation after CJP–TiN intervention. A) Venn diagram showing the overlap of gene expression in MKN45 cells pretreated with PBS and CJP–TiN+L. B) Volcano plot indicating elevated expression of the key genes related to the PANoptosis and immunogenic cell death pathways in the CJP–TiN+L group. C) Heatmap of differentially expressed genes (DEGs) between the control and CJP–TiN+L treatment groups. Significant differences in gene expression levels between the two groups can be observed, with red representing the upregulated genes and blue representing the downregulated genes. D) KEGG enrichment analysis shows that DEGs are significantly enriched in multiple signaling pathways. E) Schematic representation of the process mechanism. F) Changes in the expression of key proteins (Bax, Bcl‐2, cleaved caspase‐3/7/8, p‐MLKL, p‐RIPK1, GSDME‐N, and HMGB1) in the AGS and MKN45 cells under different treatment conditions. Significant differences in the expression of PANoptosis‐related proteins under the various treatment conditions suggest the role of CJP–TiN+L in inducing PANoptosis. G,I) ROS generation in AGS and MKN45 cells was significantly reduced in the G4 group (NAC + CJP–TiN + L) compared to the G2 group (CJP–TiN + L). (^***^ *p* < 0.001). H,J) Dead cell MFI in AGS and MKN45 cells showed a significant decrease in the G4 group compared to the G2 group. (^*^ *p* < 0.05). K,L) Apoptosis rate in AGS and MKN45 cells was higher in the G2 group and decreased in the G4 group (^***^ *p* < 0.001). Apoptosis in AGS and MKN45 cells depends on ROS generation, as NAC, an ROS scavenger, reduced apoptosis when combined with CJP–TiN+L. Groups: G1: PBS; G2: CJP‐TiN + L; G3: NAC; G4: NAC + CJP–TiN + L. Data are presented as mean ± SEM (*n* = 3).

To further elucidate the mechanism of cell death in GC cells after CJP–TiN+L treatment, the transcriptomic expression differences were compared between the PBS group and the CJP–TiN+L group. Out of a total of 13 977 genes (**Figure** **4**A), 469 genes were found to exhibit significant changes in the CJP–TiN+L group. Compared to the PBS group (Figures 4B,C), the CJP–TiN+L group contained 1049 upregulated genes and 1335 downregulated genes, with the key PANoptosis and immunogenic‐related genes (i.e., CASP3, CASP7, CASP8, MLKL, and HMGB1) being significantly upregulated in the CJP–TiN+L treatment group. To validate the reliability of the RNA‐seq results, qPCR analysis was performed on selected key PANoptosis‐ and immunity‐related genes (including CASP3, CASP7, CASP8, MLKL, and HMGB1), as well as CYP1A1, the gene with the highest fold change. The expression is shown in Figure S8 (Supporting Information), and the results were consistent with the RNA‐seq data.

KEGG (Kyoto Encyclopedia of Genes and Genomes) enrichment analysis revealed the presence of significantly enriched signaling pathways, including the MAPK, HIF‐1, and p53 signaling pathways, all of which are closely related to ROS, apoptosis, and the immune response (Figure 4D). To further validate the transcriptomic results, WB experiments were performed to detect the expression of PANoptosis‐related proteins (Figure 4F). To further confirm the changes in HMGB1 expression at the cellular level, confocal microscopy was performed on AGS and MKN45 cells across different treatment groups. As shown in the Figure S9 (Supporting Information), HMGB1 expression was markedly elevated following CJP–TiN+L treatment. Notably, it was found that the CJP–TiN nanoparticles significantly enhanced the effectiveness of PDT under laser irradiation, indicating their potential value in anti‐tumor applications. Analysis of the supernatant obtained from the AGS and MKN45 cells showed that the CJP–TiN+L group contained the highest ATP concentration and the highest release rates of IL‐1β and IL‐18. Compared to other treatment groups (i.e., the JB, JB+Ce6+L, CJP‐TiN groups), the CJP–TiN+L group exhibited a significantly enhanced inflammatory response and promoted cell activation (Figure S10, Supporting Information). The results indicated that in the AGS and MKN45 cells, CJP–TiN+L treatment significantly upregulated the expression of Bax, Cleaved Caspase‐3/7/8, p‐MLKL, and GSDME‐N, whilst downregulating expression of the inhibitory protein Bcl‐2. Furthermore, the expression of HMGB1 in the CJP–TiN+L group increased, validated by immunofluorescence cell staining. This indicates that CJP–TiN+L treatment induced an immunogenic cell death (ICD) effect and confirmed that CJP–TiN+L enhances cell death through PANoptosis activation. Moreover, Figure S11G (Supporting Information) illustrates the impact of N‐acetylcysteine (NAC) pretreatment on the expression of key proteins in the AGS and MKN45 cells after CJP–TiN+L treatment. From the DCHF–DA staining results (Figure 4G,I), it can be seen that CJP–TiN+L treatment significantly increased ROS production, while NAC pretreatment inhibited this phenomenon. Additionally, Calcein‐AM/PI double staining (Figure 4H,J) and Annexin V/PI FCM analysis (Figure 4K,L) showed that CJP–TiN+L treatment induced cell death, while NAC pretreatment reduced the rates of apoptosis and death. Overall, the anti‐tumor mechanism of CJP–TiN+L relies on ROS generation (Figure S11, Supporting Information). The cell thermal shift assay (CETSA) demonstrated that JB enhanced the thermal stability of Caspase‐8, indicating a direct interaction. SPR analysis further confirmed this binding, showing a concentration‐dependent response with a dissociation constant (KD) of 27 nm. These results confirm the specific and high‐affinity interaction between JB and Caspase‐8, as shown in Figure S12 (Supporting Information).

### 2.5 Evaluation of the Biological Safety of CJP–TiN for Tumor Therapy

To evaluate the systemic toxicity of CJP–TiN, multiple biochemical and histological assessments were performed, namely blood indices (WBC, Lymph, Gran) (Figure S13A, Supporting Information), erythrocyte parameters (HGB, HCT, MCV, MCH, MCHC, and RDW) (Figure S13B, Supporting Information), liver function markers (ALT, AST, TBIL, and ALB), kidney function markers (UREA, CREA) (Figure S13C, Supporting Information), and platelet‐associated parameters (PLT, MPV, PDW, and PCT; Figure S13D, Supporting Information), among others. It was found that, when compared to the saline group, no significant abnormalities were detected in any of the treatment groups, suggesting that CJP–TiN exhibits a low systemic toxicity in vivo. Hemolysis experiments were also employed to assess the blood compatibility of CJP–TiN at different concentrations (1, 5, 10, and 50 µg mL^−1^). The relative hemolysis rate at each concentration was < 5% (Figure S13E, Supporting Information), indicating that CJP–TiN demonstrates a good blood compatibility. Hematoxylin and eosin (H&E) staining of tissues from the heart, liver, spleen, lungs, and kidneys (Figure S13F, Supporting Information) revealed no significant tissue damage or inflammatory infiltration in any treatment group compared to the saline group, demonstrating that the formulation exhibits a good biocompatibility and does not induce organ toxicity, thereby confirming its suitable safety profile for use in systemic administration.

### 2.6 In Vivo Biodistribution and Antitumor Effects of CJP–TiN

**Figure 5:**
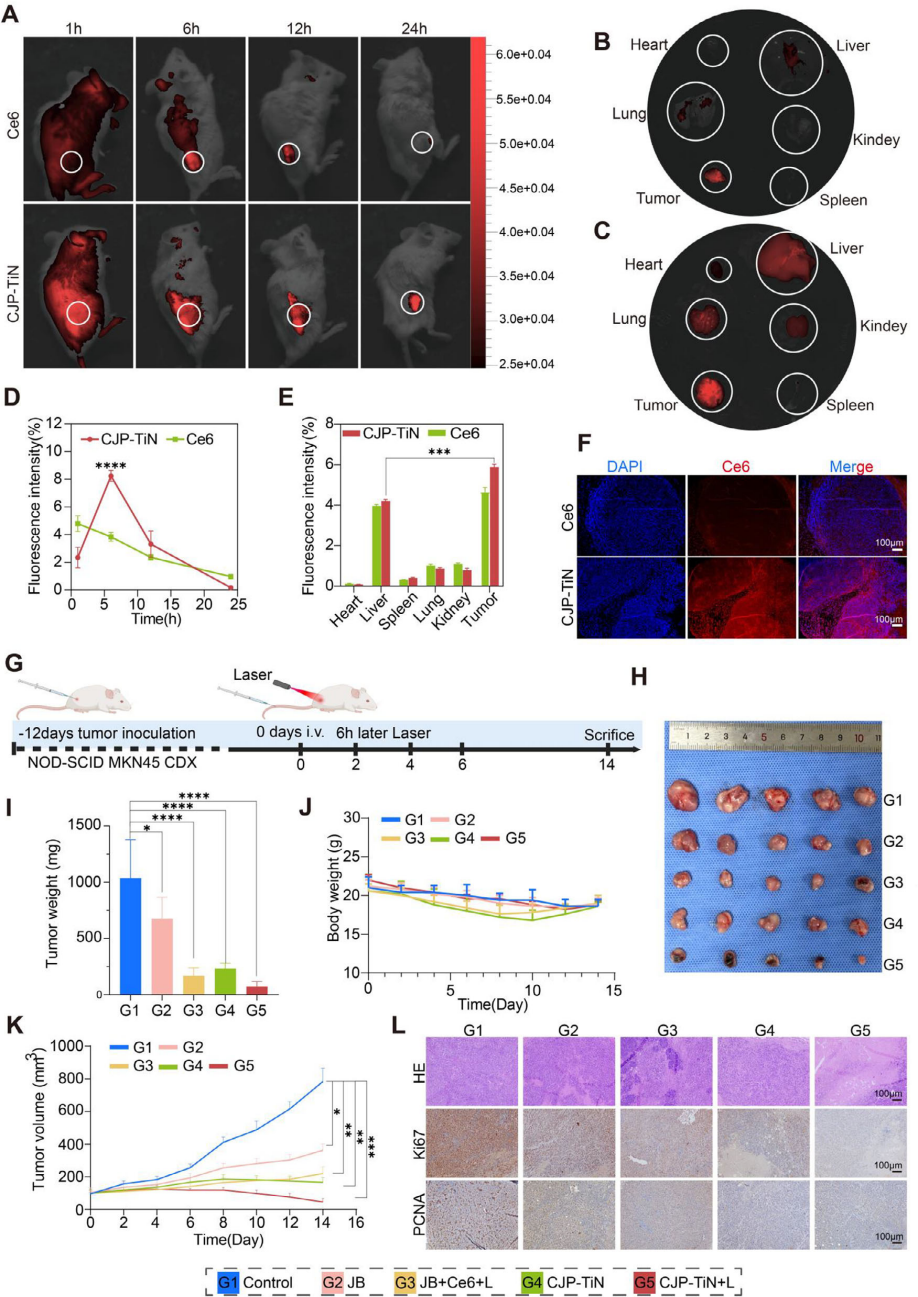
In vivo biodistribution and antitumor effects of CJP–TiN. A) IVIS images showing the distribution of Ce6 and CJP–TiN in MKN45‐CDX mice at different time points. D) The results of fluorescence intensity analysis are presented for the tumors (*n* = 3). ^****^ *p* < 0.0001. B,C,E) Biodistribution and fluorescence intensity analysis of Ce6 and CJP–TiN in MKN45‐CDX tumors and major organs 24 h after intravenous injection. The results show that CJP–TiN significantly enhances tumor accumulation compared to Ce6 (*n* = 3). ^***^ *p* < 0.001. F) IF results for Ce6 and CJP–TiN in the subcutaneous MKN45‐CDX tumor tissues 24 h after intravenous injection. Scale bar: 100 µm. G) Schematic diagram of PDT treatment using CJP–TiN in MKN45‐CDX tumor‐bearing mice. H–K) Images of the tumors excised from the mice, in addition to the tumor weight, mouse body weight, and tumor volume growth curves after treatment (*n* = 5). ^*^ *p* < 0.05, ^**^ *p* < 0.01, ^***^ *p* < 0.001, ^****^ *p* < 0.0001. L) Tumor growth inhibition observed by H&E, Ki67, and PCNA staining. Scale bar: 100 µm.

To further verify the tumor targeting and PDT efficacy of CJP–TiN in vivo, its biodistribution, safety, and tumor inhibition capacity were assessed in the MKN45‐CDX mouse model. **Figure** **5**A presents the in vivo fluorescence imaging results obtained for Ce6 and CJP–TiN at various time points (i.e., 1, 6, 12, and 24 h) following intravenous injection via the mouse tail. At 6 h, the fluorescence of CJP–TiN peaked at the tumor site, while the fluorescence intensity of Ce6 was significantly lower, and CJP–TiN continued to effectively accumulate in tumor tissue at 24 h (Figure 5D). After 24 h, the mice were euthanized and dissected, showing weak fluorescence signals in the tumor and major organs, including the liver, kidneys, and spleen, which indicated low accumulation levels of Ce6 in these tissues (Figure 5B). In contrast, Figure 5C shows a significant enhancement in the fluorescence signals for the tumor area after 24 h, indicating that CJP–TiN possesses a higher targeting ability and accumulation efficiency (see also Figure 5E). Furthermore, fluorescence sections of the tumor tissues demonstrated stronger Ce6 fluorescence in the CJP–TiN group (Figure 5F), demonstrating that CJP–TiN can effectively accumulate in the tumor tissue, showing good targeting and delivery effects. To investigate the benefits of this drug delivery platform in tumors, MKN45‐CDX mice with a tumor volume of ≈100 mm^3^ were randomly allocated to different treatment groups for intervention (Figure 5G). After 14 d of treatment, the tumors were excised and their volumes were measured. Significant tumor growth was detected in the control and JB groups, indicating limited therapeutic efficacies. In contrast, the tumor volumes in the JB+Ce6+L, CJP–TiN, and CJP–TiN+L groups were significantly reduced, especially in the CJP–TiN+L group, which exhibited the most pronounced inhibitory effect (Figure 5H,I). The temporal changes in tumor volume indicated significant suppression of tumor growth in the CJP‐TiN+L group (^***^ *p* < 0.001), demonstrating the strongest anticancer effect. Furthermore, H&E staining (Figure 5L) revealed severe destruction of the tumor tissue in the CJP–TiN+L group, accompanied by obvious necrosis, and showing significant tissue damage compared to the control, JB, JB+Ce6+L, and CJP–TiN groups. Moreover, Ki67 and PCNA staining results indicated a significant reduction in the quantity of proliferation markers in the CJP–TiN+L group, indicating that this treatment approach effectively inhibited tumor cell proliferation. During the entire treatment period, monitoring of the mouse body weight revealed no significant weight loss in any of the groups (Figure 5J). These results collectively indicate that the combination of CJP–TiN with PDT results in a significant antitumor effect.

### 2.7 In Vivo Study of the Abscopal Effects of CJP–TiN

**Figure 6:**
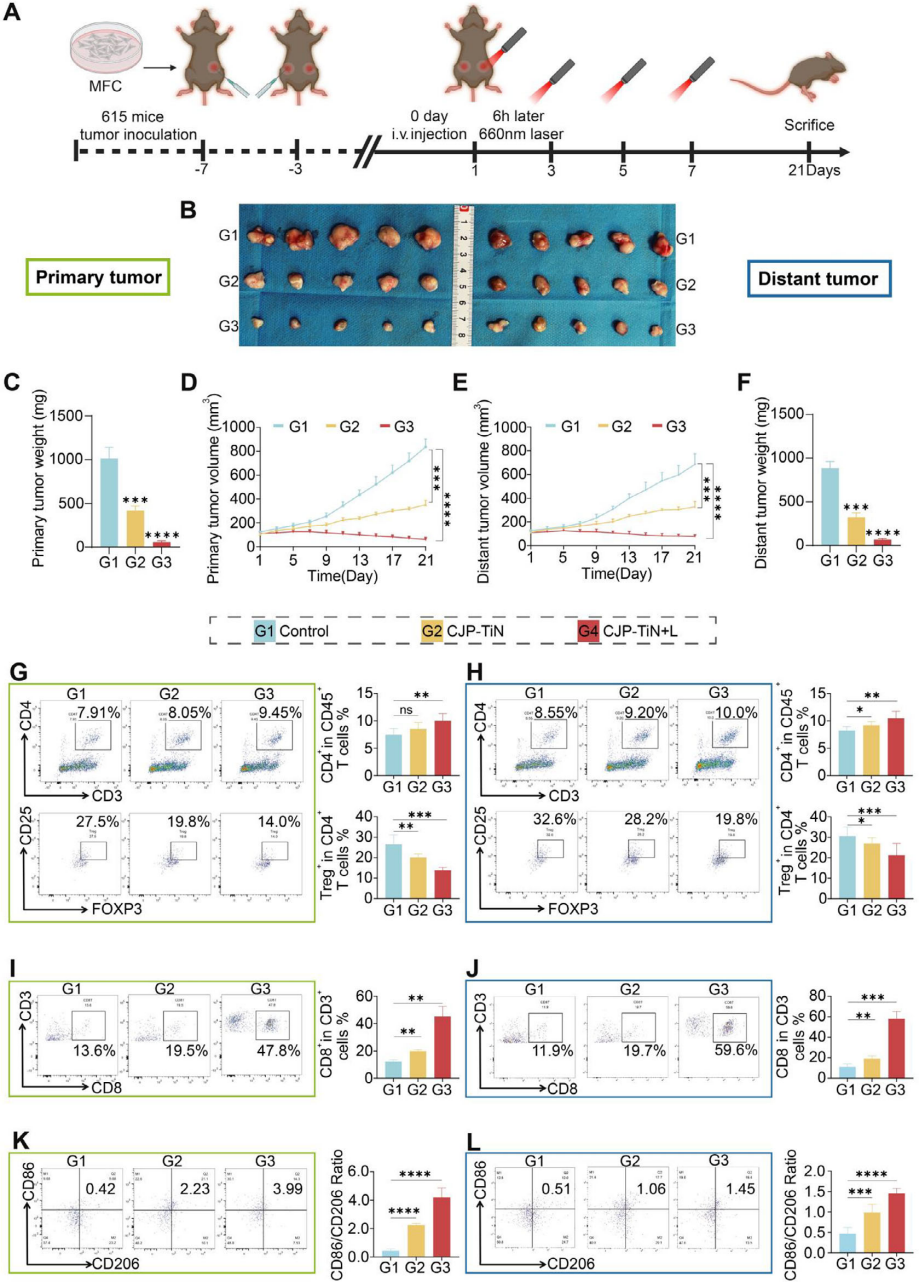
Abscopal effect of CJP–TiN‐mediated PDT. A) The diagram illustrates an MFC‐615 tumor study timeline. The tumor on the left side was the primary site, and the tumor on the right side was the distant tumor. B–F) Tumors excised from both sides of the mice, in addition to the tumor weights and tumor volume growth curves after treatment (*n* = 5). ^***^ *p* < 0.001, ^****^ *p* < 0.0001. G,H) Representative FCM histograms and quantitative analysis of CD4+ and Tregs in tumors on both sides of the MFC tumor (*n* = 3). ^*^ *p* < 0.05, ^**^ *p* < 0.01, ^***^ *p* < 0.001, ns indicates no statistical significance. I,J) Representative flow cytometry histograms and quantitative analysis of CD3+ and CD8+ cells in tumors on both sides of the MFC tumor (*n* = 3). ^**^ *p* < 0.01, ^***^ *p* < 0.001. K,L) FCM was performed on tumors from both sides of the MFC tumor to detect CD86+CD206−M1 macrophages and CD86−CD206+M2 macrophages, and to quantitatively analyze the percentages of M1 and M2 macrophages (*n* = 3). ^***^ *p* < 0.001, ^****^ *p* < 0.0001.

The pronounced tumor‐inhibiting effect of the CJP–TiN+L group was observed not only in MKN45‐CDX primary tumors, but also in distant tumors, further confirming its efficacy in controlling metastatic tumors. More specifically, in the bilateral tumor MFC‐615 model (**Figure** **6**A), it was observed that compared with the control and CJP–TiN groups, mice in the CJP–TiN+L treatment group receiving irradiation only at the primary site showed significantly reduced weights and volumes of both their primary and distant tumors (^****^ *p* < 0.0001) (Figure 6B–F). Pathological H&E, Ki67, and PCNA staining of the primary and distant tumors was also performed, as shown in Figure S14 (Supporting Information). To further explore the immune regulatory mechanisms of CJP–TiN in the tumor microenvironment, FCM analysis was performed on the primary and distant subcutaneous tumors (Figure 6G,H). Compared to the control group, the proportion of CD4+ T cells significantly increased in the CJP–TiN+L group, while the proportion of Treg cells (CD25+FOXP3+) notably decreased (^**^ *p* < 0.01) in the CJP–TiN+L group, and the CD8+ T cell levels significantly increased (Figure 6I,J), representing a significant enhancement compared to the control and CJP‐TiN groups (^**^ *p* < 0.01, ^***^ *p* < 0.001). These results indicate that CJP–TiN+L treatment effectively enhanced immune activation in the tumor microenvironment, reduced the infiltration of immunosuppressive cells, and increased CD8+ T cell infiltration, which may enhance the antitumor immune response and provide immunological support for effectively inhibiting tumor growth. Furthermore, the phenotypic changes in the macrophages were assessed, demonstrating a significant increase in the M1 (CD86+)/M2 (CD206+) macrophage ratio in the CJP–TiN+L group (^****^ *p* < 0.0001) (Figure 6K,L). The gating strategy for flow cytometry is shown in Figure S15 (Supporting Information). These results suggest that the developed treatment approach effectively promotes macrophage polarization toward the M1 antitumor phenotype, thereby enhancing the antitumor immune response.

**Figure 7:**
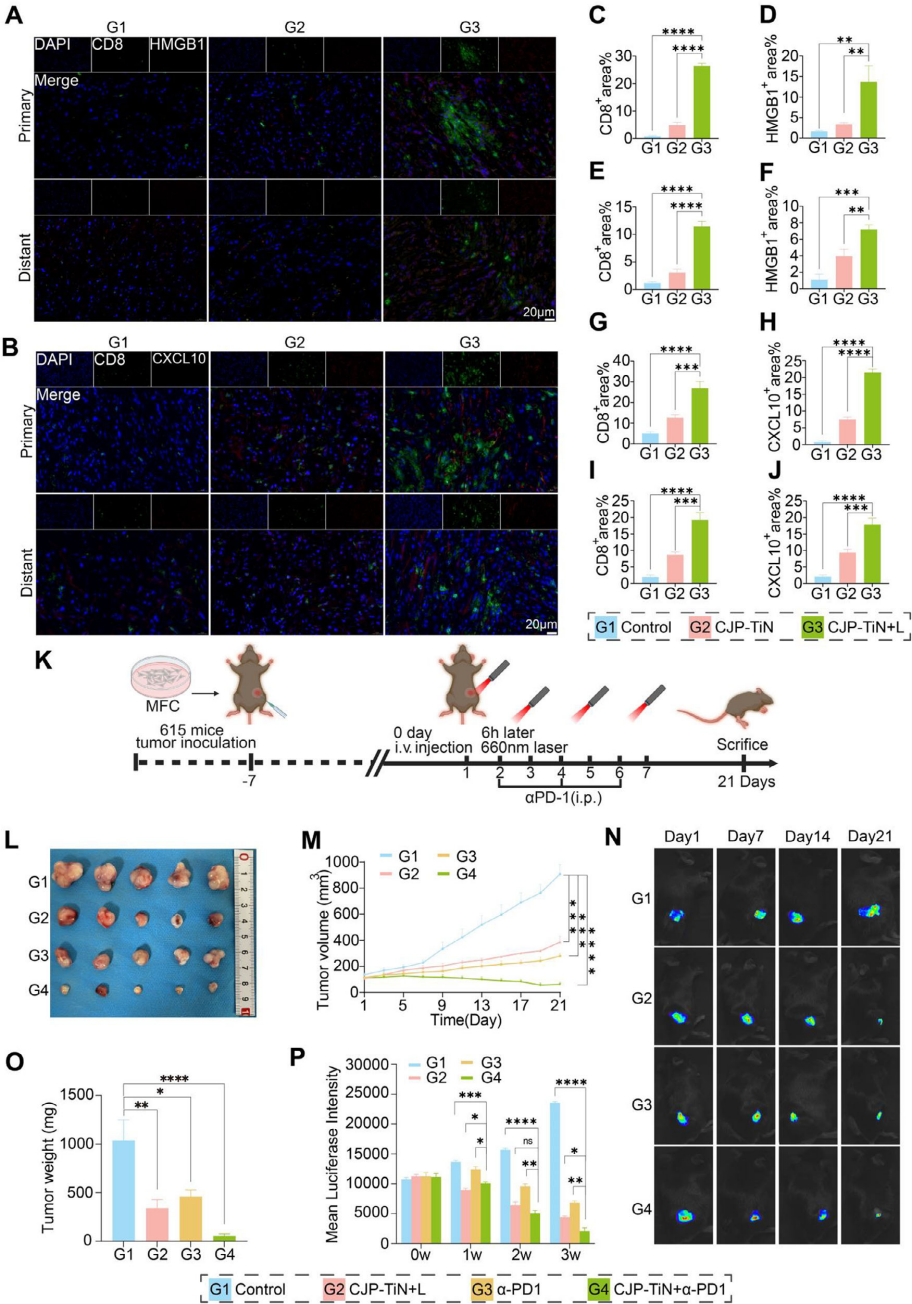
Bilateral tumor IF measurements to evaluate the abscopal effects and synergistic enhancement of immune effects by CJP–TiN in combination with α‐PD1 blockade therapy. A) Multiplex IF images of CD8+ (green) and HMGB1 (red) in tumors on both sides of the MFC tumor. B) Multiplex IF images of CD8+ (green) and CXCL10 (red) in tumors on both sides of the MFC tumor. C–J) Statistical analysis was performed for the fluorescence intensity of CD8+, HMGB1, and CXCL10 in tumors on both sides of the MFC tumor (*n* = 3). ^**^ *p* < 0.01, ^***^ *p* < 0.001, ^****^ *p* < 0.0001. K) Schematic diagram representing the combined treatment of MFC‐615 tumor model mice with CJP–TiN and α‐PD1. L,M) Images of the tumors excised from the mice and the corresponding tumor volume growth curves after treatment (*n* = 5). ^***^ *p* < 0.001, ^****^ *p* < 0.0001. N) IVIS evaluation of the antitumor efficacy under different treatment approaches (*n* = 5 per group). O) Weights of the tumors excised from the mice after treatment. P) IVIS fluorescence statistical analysis of the antitumor efficacy under different treatment approaches (*n* = 5). ^*^ *p* < 0.05, ^**^ *p* < 0.01, ^***^ *p* < 0.001, ^****^ *p* < 0.0001. ns indicates no statistical significance.

To further explore the mechanism of the distant effect, the distributions of CD8+ T cells, HMGB1, and CXCL10 in the tumor tissue were assessed by means of immunofluorescence (IF) staining (**Figure** **7**A,B). In the CJP–TiN+L group, the expression levels of CD8+ T cells, HMGB1, and CXCL10 were significantly elevated in both the primary and distant tumor regions (Figures 7C–J), demonstrating that CJP–TiN+L treatment significantly enhanced T cell infiltration and chemokine expression, in addition to promoting macrophage polarization toward an antitumor phenotype. These results indicate the potential of this approach to reshape the tumor immune microenvironment and enhance antitumor immune responses. Moreover, they provide new insights into the distant effect mechanism of CJP‐TiN and establish a theoretical basis for combination immunotherapy.

### 2.8 Combination of CJP‐TiN with α‐PD1 Blockade Therapy to Synergistically Enhance the Immune Effects

Based on the above results, it was clear that CJP–TiN+L markedly enhanced immune cell infiltration and chemokine expression, establishing a basis for its use in combination with immunotherapy, and suggesting the potential for further exploration of synergistic mechanisms. In the MFC‐615 mouse model, the first intravenous drug injection was administered after tumor inoculation, and this was followed by 660 nm laser irradiation after 6 h, along with three consecutive doses of the PD‐1 antibody and subsequent laser irradiation. On day 21, the mice underwent endpoint observation and tissue sampling (Figure 7K), and it was found that, compared to the control, CJP–TiN+L, and α‐PD1 groups, the combination treatment group showed the most significant tumor inhibition effect (^****^ *p* < 0.0001; Figure 7L, M, O). In combination with the IVIS imaging results (Figure 7N), it was clear that the CJP–TiN+α‐PD1+L group exhibited the most pronounced tumor inhibition effect throughout the experiment (Figure 7P). Moreover, HE, Ki67, and PCNA staining of the primary tumor (Figure S16, Supporting Information) indicated that the combination of CJP–TiN+L with the α‐PD1 antibody significantly inhibited tumor cell proliferation, emphasizing its superiority in terms of synergistic antitumor effects. Importantly, this combination not only enhanced tumor inhibition, but also significantly increased immune activation in the tumor microenvironment, indicating its great potential utility in cancer treatment.

## 3 Discussion

Owing to their small size, excellent biosafety profiles, good drug‐loading capacities, and desirable physical properties, nanoparticles are increasingly being used as carriers in novel tumor treatment approaches. In this context, liposomes are a type of nanoscale biodegradable drug delivery system that can be used for targeted delivery to specific cells, thereby reducing the systemic side effects.^[^ ^26^ ^]^ The encapsulation of a drug by liposomes can enhance the drug efficacy and reduce its toxicity. Consequently, this approach has been widely applied in anticancer chemotherapy, such as in the encapsulation of doxorubicin for inhibiting cell growth.^[^ ^27^ ^]^ Previously, studies have shown that free JB exhibits poor pharmacokinetics and a weak antitumor efficacy; however, using black phosphorus quantum dots as JB carriers, Zhao et al.^[^ ^28^ ^]^ showed that the prepared BPQDS@JB significantly induced apoptosis in tumor tissues, thereby achieving lymphoma inhibition.

The CJP–TiN employed herein possesses multiple properties, the first being targeting. More specifically, iRGD is a tumor tissue‐specific penetrating peptide that has the ability to target α_v_β_3_ on the tumor surface, and has been shown to selectively deliver chemotherapeutic drugs and imaging agents to specific tumor sites.^[^ ^29^, ^30^ ^]^The WB and IHC analyses of clinical GC tissues and cells indicated high expression levels of α_v_β_3_ on the surfaces of GC cells, thereby rendering it an ideal target for the current system. In this context, Zhou et al.^[^ ^31^ ^]^ previously modified red blood cells using DSPE‐PEG‐iRGD to target α_v_β_3_ on the surfaces of GC cells to enhance the sensitivity of radiotherapy. In addition, Zhang et al.^[^ ^32^ ^]^ utilized the active targeting capability of iRGD to enhance the anticancer efficacy by increasing the drug delivery efficiency. The second desirable property of CJP–TiN is its pH/ROS responsive nature. Under hypoxic conditions, tumor cells preferentially undergo glycolysis, producing protons (H^+^ ions), which render the tumor tissue slightly acidic (pH 6.5) compared to normal tissue (i.e., pH 7.48).^[^ ^33^ ^]^ Although ROS take part in cell signaling under normal physiological conditions, excessive ROS levels can lead to oxidative stress, which is linked to the development of various diseases, including cancer.^[^ ^34^ ^]^ Thus, in the current study, liposomes capable of promoting selective drug release within tumor tissues were developed. This approach was based on the lower pH and elevated ROS levels of tumor cells, along with the principle that substantial amounts of ROS are generated post‐PDT. In a similar example, Liu et al.^[^ ^24^ ^]^ loaded pH‐sensitive lipid DSPE‐PEOz onto platelet membranes, imparting the material with both a strong tumor affinity and an excellent pH responsiveness in the tumor microenvironment. Furthermore, Hao et al.^[^ ^35^ ^]^ utilized the ROS responsiveness of the TK bond to link camptothecin to a photosensitizer, ultimately achieving controlled release upon 660 nm laser irradiation. In another study, Huang et al.^[^ ^36^ ^]^ used mPEG‐TK‐PBAE copolymers to load Ce6 and TPL. Utilizing the pH sensitivity of PBAE and the ROS sensitivity of the TK bond, the PDT‐induced oxidative stress was enhanced, and TPL‐induced apoptosis was promoted in HepG2 cells, leading to synergistic anticancer effects in vitro. Moreover, Chen et al.^[^ ^37^ ^]^ developed a multifunctional nanoplatform, namely Fe^2+^@UCM‐BBD, to facilitate tumor cell internalization via an enhanced permeability and retention, which led to the release of doxorubicin in the acidic tumor microenvironment. Notably, this system was characterized by the self‐supply of H_2_O_2_, thereby enhancing the efficacy of the chemodynamic therapy.

In the current system, both PEOz and the TK bond were employed for the first time, yielding CJP–TiN, which exhibited a good stability, strong tumor accumulation properties, ideal endocytosis, and a superior drug release performance, as validated through particle size stability, drug release, and cell endocytosis experiments. It was observed that CJP–TiN demonstrated a significant efficacy in the PDT of GC, offering a promising new treatment option for advanced GC, which primarily relies on systemic chemotherapy.^[^ ^38^ ^]^ Existing research indicates that PANoptosis, similar to other forms of cell death, can induce tumor cell death and suppress tumor progression, thereby rendering it a promising target for cancer therapy.^[^ ^39^ ^]^ Lin et al.^[^ ^40^ ^]^ found that YBX1 promotes tumor cell growth and inhibits PANoptosis in AGS cells. Thus, by inhibiting the expression of YBX1, its ability to promote cell proliferation and inhibit PANoptosis was weakened, ultimately reducing the cell resistance to oxaliplatin chemotherapy. Previous investigations performed in our group, which involved both in vivo and in vitro experiments, along with molecular docking studies, demonstrated that JB effectively targets and activates the PANoptosis switch, caspase‐8.^[^ ^17^ ^]^ Upon irradiation, the photosensitizer itself generates ROS, and the high ROS environment causes the TK bond to break, leading to drug release from the liposome, and demonstrating a controlled release effect; additionally, JB itself acts as an agonist of caspase‐8. Using ROS inhibitors, it was found that ROS are involved in the upstream pathway of caspase‐8 activation, and as a result, the combined use of Ce6 and JB activated the ROS‐caspase8/PANoptosis signaling pathway, inducing PANoptosis in GC cells. Notably, among the differentially expressed genes, CYP1A1 exhibited the highest fold change following CJP–TiN+L treatment. A well‐established downstream target of the aryl hydrocarbon receptor (AhR), CYP1A1 is involved in the biotransformation of lipophilic xenobiotics and has been associated with ROS generation and oxidative stress‐induced cytotoxicity.^[^ ^41^, ^42^ ^]^ Its pronounced upregulation suggests a metabolic response to xenobiotic stimulation, potentially enhancing intracellular ROS levels and thereby amplifying PANoptotic signaling.^[^ ^43^ ^]^ These results indicate that, beyond its metabolic function, CYP1A1 may act as a critical mediator of ROS‐driven cell death in GC under CJP–TiN treatment.

During tumor radiotherapy, the abscopal effect (AE) is observed, wherein unirradiated lesions also shrink along with the irradiated ones.^[^ ^44^ ^]^ The occurrence of the AE is similar to the mechanism of the inflammatory response generated by tumor cells post‐irradiation, with immune system activation being the primary reason for radiation‐induced AE. After tumor cell injury, ICD occurs, releasing multiple DAMPs with immunostimulatory properties, including IFN‐I, CRT, HMGB1, ATP, and HSP.^[^ ^45^ ^]^ The DAMPs released following tumor cell ICD can promote the transition of M2‐type tumor associated macrophages (TAMs) to M1‐type in the distant tumor microenvironment. DAMPs are known to activate dendritic cells (DCs), further promoting maturation of the CD4+ T cells into cytotoxic T lymphocytes (CTLs), which secrete IFN‐γ and TNF‐α, and drive TAM polarization towards the M1 phenotype.^[^ ^46^ ^]^ Additionally, in the distant tumor microenvironment, the HMGB1 released from irradiated tumor cells binds to the TLR4 receptors on the TAMs, further promoting M1 polarization. These polarized M1‐type TAMs secrete various immune factors (e.g., IL‐1β, IL‐6, and TNF‐α), thereby enhancing the immunosuppressive environment and inhibiting distant tumor growth and metastasis.^[^ ^47^ ^]^ CJP–TiN is also notable for its role in activating immunity in vivo. Previous studies have indicated that PDT‐induced immunity mainly occurs via two mechanisms. First, by activating the innate immunity, PDT treatment can lead to an acute inflammatory response, enhancing neutrophil circulation,^[^ ^48^ ^]^ and second, by activating the adaptive immunity, DAMP expression and release are induced, triggering ICD and stimulating the immune response.^[^ ^49^ ^]^ The immunosuppressive microenvironment, comprising Treg cells, myeloid‐derived suppressor cells, and M2‐type TAMs, has consistently been a significant factor that impacts the efficacy of PDT. More specifically, during the recovery phase of PDT treatment, the levels of M2‐type macrophages significantly increase, which may lead to tumor recurrence.^[^ ^50^ ^]^ In the current study, analysis of the immune microenvironments of the primary and distant tumors in MFC‐615 mice revealed an increase in the CD3+, CD4+, and CD8+ T cell numbers and proportions, along with reduced Treg levels, and evidence of M1 macrophage polarization. These alterations ultimately lead to the AE, suggesting robust immune activation by CJP‐TiN. Moreover, it has been reported that the T cells activated by radiotherapy are recruited to new tumor sites by the CXCL10 secreted by tumor cells,^[^ ^51^ ^]^ and the release of HMGB1 facilitates the synthesis of pro‐inflammatory factors, including type I interferons, subsequently guiding CXCL10 production. Ultimately, CD4+ T cells proliferate into cytotoxic T cells and natural killer cells, releasing large quantities of chemokines and cytokines, and promoting cell infiltration. This phenomenon was similarly observed in the current CJP–TiN+L group, wherein high levels of CD8+ T cells, HMGB1, and CXCL10 were detected in both the primary and distant tumors. It can therefore be inferred that CJP–TiN can convert some “cold tumors” into “hot tumors” under laser irradiation, and in combination with α‐PD1, the tumors volumes were reduced, indicating the effectiveness of this immunotherapeutic approach.

## 4 Conclusion

In summary, the developed CJP–TiN system stably delivers Jolkinolide B and Chlorin e6 via functionalized liposomes, effectively targeting tumor sites, triggering drug release through pH/ROS (reactive oxygen species) responsiveness, and enhancing the synergistic effects of the drug treatment. Gastric cancer (GC) growth was significantly inhibited both in vitro and in vivo, and it was deduced that this was occurred through the ROS‐caspase8/PANoptosis pathway. Additionally, a distinct abscopal effect was observed in metastatic models. Furthermore, CJP–TiN was found to modulate the tumor microenvironment, promote antitumor immune responses, and effectively mitigate immunosuppression. Overall, it was deduced that the CJP–TiN‐based photodynamic therapy approach developed herein provides a promising, safe, and efficient strategy for treating GC, with the potential to overcome the limitations of current therapies.

## 5 Experimental Section

### Materials and Tissue Samples

JB was supplied by Shanghai Yuanye Biotechnology Co., Ltd. Ce6, soybean lecithin, cholesterol, DSPE‐TK‐NH_2_, COOH‐PEOz_2k_‐OH, PyBOP, Triethylamine, Dimethylformamide, Succinic anhydride, Diethyl Ether, 1‐Ethyl‐3‐(3‐dimethylaminopropyl)carbodiimide, N‐Hydroxysuccinimide and iRGD peptide were sourced from Xi'an Ruixi Biological Technology Co., Ltd. The RPMI‐1640 medium was purchased from Hyclone (USA), while the 10% fetal bovine serum was obtained from ExCell Bio, the 1% penicillin/streptomycin was purchased from Beyotime, the CCK‐8 and ROS detection kits were obtained from Biosharp (China), and the live/dead cell double‐staining kit was purchased from Yesen Biotechnology Co., Ltd. NAC was obtained from MCE (HY‐B0215, Shanghai, China). Detailed information could be found in Table S6 (Supporting Information). The study was approved by the ethics committee of the Second Hospital of Lanzhou University (2024A‐788), and informed consent was obtained from all human participants. Paraffin‐embedded and fresh GC tissues were collected for target validation.

### Cell Lines and Animals

All cell lines were sourced from the Key Laboratory of Environmental Oncology, Gansu Province. The AGS and MKN45 cell lines were cultured at 37 °C in RPMI 1640 medium containing 10% fetal bovine serum. Female NOD/SCID mice (5–6 weeks old) were purchased from Beijing Vital River Laboratory Animal Technology Co., Ltd., and 5–6 week‐old male 615 mice were purchased from the Institute of Hematology, Chinese Academy of Medical Sciences. The animal experiments were reviewed and approved by the Animal Experiment Ethics Committee of the Second Hospital of Lanzhou University (D2022‐339).

### Molecular Docking

The molecular docking of iRGD (PubChem CID: 134 611 625) with the α_v_β_3_ protein (PDB ID: 4MMX) was conducted using AutoDock Vina 1.1.2. Pre‐processing of the protein was carried out using PyMol 2.4, and the generated PDBQT file served as the input for the docking simulation. The docking box size was 58 × 48 × 40 Å, with a grid spacing of 0.719 Å, and center coordinates at x: 17.432, y: 42.6, z: 41.031. The docking results yielded 10 best conformations, and the conformation with the lowest binding energy was selected as the optimal conformation. The results were analyzed and visualized using PyMol 2.4.

### Immunohistochemistry and Immunofluorescence

The collected tissues were fixed in 37% neutral formaldehyde, dehydrated, and embedded in paraffin. Sections (4 µm) were cut, baked, dewaxed, and rehydrated. Antigen retrieval and blocking were performed, followed by incubation with primary antibodies at 4 °C overnight. The next day, sections were washed with PBS and incubated with secondary antibodies, and tissue staining was performed using KIT‐5920 (Fuzhou Maixin Biotechnology Co., Ltd.) according to the manufacturer's instructions. Tissue immunofluorescence staining was performed according to the protocol provided with the immunofluorescence staining kit (G1259‐50T, Servicebio, China). For each section, a pathologist selected 5 high‐power fields for result interpretation and image analysis.

### Western Blot Analysis

The GC cells or tissue samples (as appropriate) were lysed using radioimmunoprecipitation assay (RIPA) buffer, and the protein concentrations were determined according to the bicinchoninic acid assay (BCA) method. Equivalent sample quantities from each group were subjected to sodium dodecyl‐sulfate polyacrylamide gel electrophoresis (SDS‐PAGE) gel electrophoresis. After transfer to a membrane, it was blocked for 1 h, incubated with the primary antibody at 4 °C overnight, washed, and subsequently incubated with the secondary antibody for 1 h. After washing, the ECL substrate was added, followed by exposure. To calculate the relative protein expression levels, the grayscale values were measured using β‐actin as the internal control.

### Synthesis of DSPE‐TK‐PEOz_2k_‐iRGD

DSPE‐TK‐NH_2_ (50 mg) was dissolved in 5 mL DMF, and COOH‐PEOz_2k_‐OH (1.1 eq.), PyBop (2.0 eq.), and triethylamine (3.0 eq.) were added. The mixture reacted at room temperature for 2 h, followed by dialysis (MWCO 2000 Da) in pure water for 24 h to obtain DSPE‐TK‐PEOz_2k_‐OH. DSPE‐TK‐PEOz_2k_‐OH (100 mg) was reacted with succinic anhydride (2.0 eq.) and triethylamine (3.0 eq.) at 40 °C for 2 h, precipitated in ice‐cold ether, and filtered to yield DSPE‐TK‐PEOz_2k_‐COOH. DSPE‐TK‐PEOz_2k_‐COOH (50 mg) was activated with EDC (1.1 eq.) and NHS (1.1 eq.) for 2 h, then reacted with iRGD peptide (1.1 eq.) and triethylamine (3.0 eq.) at room temperature for 12 h. The solution was dialyzed (MWCO 2000 Da) for 24 h and freeze dried to obtain the final product, and stored at 4 °C.

### Liposome Preparation

The liposomes were prepared using the thin‐film hydration method. Soybean phosphatidylcholine, cholesterol, DSPE‐TK‐PEOz_2k_‐iRGD, Ce6 and JB were dissolved in chloroform at a mass ratio of 1:0.15:0.12:0.1:0.1, followed by under reduced pressure at 40 °C for 15 min to create a thin film. The film was hydrated under ultrasonic irradiation for 3 min at 100 W and 42 kHz, then processed using a liposome extruder (100 nm, Mini‐Extruder, Avanti Polar Lipids, Inc.). Dialysis was conducted using a nano‐dialysis device (ND‐1, Xi'an Ruixi), and deionized water was added (5 mL final volume) to produce nanoparticles (100 nm diameter) for further in vitro and in vivo experiments. Remaining products were stored at −20 °C.

### Characterization

The liposome morphology and size were analyzed using a Hitachi HT7800 transmission electron microscope (TEM, Hitachi, Tokyo, Japan), while the PDI and zeta potential were determined by DLS (Brookhaven, NY, USA). The stability was evaluated by monitoring changes in particle size and PDI over 14 d at 37 °C (100 rpm) in both 10 mm PBS (pH 7.4) and cell culture medium containing 10% FBS. The UV‐vis absorbances of the liposomes, Ce6, and JB were determined (TU‐1810, Beijing Purity), and a standard curve was constructed. Fluorescence spectra of the liposomes and Ce6 were acquired (Infinite E Plex, Tecan, Switzerland), and the release profiles of JB and Ce6 were determined by treating the CJP–TiN solutions under different conditions: 1) H_2_O_2_ (0, 1 mm); 2) PBS solution (pH 6 or 7.4); and 3) 1 mm H_2_O_2_ + PBS solution (pH 6). The solutions were transferred into dialysis bags and immersed in the release medium (100 mL) at 37 °C, continuously shaking at 100 rpm. At specified time intervals (0, 2, 4, 8, 12, 24, 48, and 72 h), an aliquot (2 mL) of the release medium was sampled for measurement, and an equivalent volume of fresh release medium was added.

### Encapsulation Efficiency and Drug Loading Rate

The absorbance of the supernatant was recorded at 450 and 240 nm (UV‐vis spectrophotometer) to determine the concentrations of Ce6 and JB. The following formulae were used to calculate encapsulation efficiency and the drug loading rate, respectively:

(1) $$\begin{array}{ccc} & & \mathrm{Encapsulation}\;\mathrm{efficiency}\;\left(\%\right)\\ & & =\mathrm{mass}\;\mathrm{of}\;\mathrm{encapsulated}\;\mathrm{drug}/\mathrm{initial}\;\mathrm{mass}\;\mathrm{of}\;\mathrm{drug}\times 100\% \end{array}$$

(2) $$\begin{array}{ccc} & & \mathrm{Loading}\;\mathrm{efficiency}\;\left(\%\right)\\ & & =\mathrm{mass}\;\mathrm{of}\;\mathrm{encapsulated}\;\mathrm{drug}/\mathrm{total}\;\mathrm{nanoparticle}\;\mathrm{mass}\times 100\% \end{array}$$

### In Vitro Detection of Singlet Oxygen in the Liposomes

The in vitro generation of ^1^O_2_ from CJP–TiN was measured using DPBF under 660 nm excitation. DPBF (5 mm) was combined with CJP–TiN and free Ce6 (1 mL, 4 µg mL^−1^), and treated under 660 nm laser irradiation (0.5 W cm^−2^) for 0–180 s. The change in absorbance of DPBF at 415 nm was monitored concurrently using a UV‐vis spectrophotometer, and an absorbance vs time curve was generated.

### Liposomal Cellular Uptake

The liposomal cellular uptake was determined using CLSM (Zeiss LSM 880) and a high‐connotation imaging analysis system (Operetta CLS, PerkinElmer). AGS and MKN45 cells were cultured overnight at a density of 1 × 10^5^ cells mL^−1^ in either 6‐ or 96‐well plates. Subsequently, the cells were co‐incubated at 37 °C with (Ce6+JB)@NPs, (Ce6+JB)‐iRGD@NPs, (Ce6+JB)‐TK‐PEOz‐@NPs, (Ce6+JB)‐TK‐PEOz‐iRGD@NPs, or free Ce6 (4 µg mL^−1^) for 4 h. CLSM was employed to observe the cellular localization and Ce6 uptake levels in the different treatment groups, using DAPI to stain the cell nuclei. To further monitor the dynamics of CJP‐TiN uptake and identify the optimal uptake time, high‐connotation imaging system analysis was utilized to track specific AGS and MKN45 cells (4 µg mL^−1^ Ce6, 4 h incubation). The Hoechst stain (2 µg mL^−1^) was used for nuclear staining.

### ROS Determination in AGS and MKN45

AGS and MKN45 cells were cultured overnight at a density of 2 × 10^5^ cells mL^−1^ in 24‐well plates, then treated with PBS, Ce6, or CJP‐TiN (containing 4 µg mL^−1^ Ce6) for 3 h. DCFH‐DA was then added and incubated for 20 min, followed by exposure of the cells to a 660 nm laser (0.1 W cm^−2^) for 5 min. The cells were observed between 0 and 4 h using an inverted microscope (Olympus IX53). Similarly, the cells were incubated with 10 µm DCFH‐DA at 37 °C for 30 min, ensuring to avoid light exposure during staining. Following incubation, the cells were washed three times with PBS to eliminate any residual dye, and the cell suspension was collected for DCF fluorescence analysis (CytoFlex, Beckman, USA) to measure the intracellular ROS levels.

### Cell Viability Tests

Cell cytotoxicity in the AGS and MKN45 cells was assessed using the CCK‐8 kit. The cells (5.0 × 10^4^ cells mL^−1^) were seeded in 96‐well plates and exposed to various concentrations (0–8 µg mL^−1^) of Ce6, CJP–TiN, and CJP–TiN+L (JB/Ce6 molar ratio = 1:1) for 24 h. After adding the CCK‐8 reagent (10 µL) to each well, the plates were incubated for 2 h, and cell viability measurements were performed (Tecan M200 Pro, Switzerland). Under the same grouping conditions, cells were cultured in 6‐well plates and stained with the AM/PI dual‐staining kit (40747ES76, Yisheng Biotechnology Co., Ltd.) to identify live/dead cells. Cells from each group were collected, washed with PBS, and resuspended in Annexin V binding buffer to achieve a concentration of 1 × 10^6^ cells mL^−1^. Subsequently, Annexin V‐FITC (5 µL) and PI (5 µL) were added, and the samples were incubated in the dark at room temperature for 10–15 min. Following incubation, the fluorescence signals of Annexin V‐FITC and PI were detected using a flow cytometer to determine the proportion of apoptotic cells.

### RNA‐sequencing

MKN45 cells (5.0 × 10^4^ cells mL^−1^) were cultured in 6 mm dishes and treated with CJP‐TiN (containing 4 µg mL^−1^ of Ce6) for 24 h. The cells were then exposed to a 660 nm laser (0.1 W cm^−2^ for 5 min) and incubated for another 24 h. Afterward, total RNA was extracted from both the cells and the supernatant, and RNA sequencing was performed at Majorbio Biotechnology Co., Ltd. RNA‐seq libraries were sequenced on a NovaSeq X Plus (PE150) using the NovaSeq Reagent Kit. TPM values were estimated by RSEM. DEGs were identified with DESeq2 (|log2FC| ≥ 1, FDR < 0.05). KEGG enrichment was performed via SciPy (*p* < 0.05, Bonferroni‐corrected).

### Enzyme‐Linked Immunosorbent Assay (ELISA)

ELISA was performed using pre‐coated 96‐well plates. Cell culture supernatants and standards were added to the wells, followed by incubation with detection antibodies and HRP‐conjugated secondary antibodies. After washing, TMB substrate was added and the reaction was terminated with stop solution. Absorbance was measured at 450 nm with a reference at 570 or 630 nm. Sample concentrations were calculated using a four‐parameter logistic (4‐PL) standard curve.

### CJP–TiN Biodistribution Study

A CDX model of GC was generated by subcutaneously injecting MKN45 cells (1 × 10⁷ cells mL^−1^, 100 µL per mouse) into the right flanks of female NOD/SCID mice (18–20 g). The mice were randomly allocated into groups and administered PBS, free Ce6, or CJP‐TiN (Ce6 at 10 mg kg^−1^) via tail vein injection. Imaging was performed at various time points post‐injection (1, 6, 12, and 24 h) using an in vivo imaging system (Invivo Smart‐LF, Vieworks, Korea). Tumor tissues and major organs were collected from the 24 h group, and samples of the tumor tissue were prepared into 6 µm sections and stained with DAPI.

### Toxicity Evaluation of CJP–TiN

MKN45‐CDX mice were injected via the tail vein with saline, free JB (40 mg kg^−1^), free Ce6 (10 mg kg^−1^), Lip‐TK‐PEOz_2k_‐iRGD, or CJP‐TiN (JB = 40 mg kg^−1^, Ce6 = 10 mg kg^−1^). After 48 h, mice were euthanized, and blood was collected via orbital puncture, incubated at 4 °C for 30–120 min, centrifuged (4 °C, 3000 rpm, 10 min), and the serum was aliquoted (150 µL), flash‐frozen in liquid nitrogen, and stored at −80 °C for biochemical analysis. Simultaneously, major organs from the mice were harvested for H&E staining to assess the degree of organ damage.

### In Vivo Anti‐Tumor Effects of CJP–TiN

Using the MKN45‐CDX model, when the average tumor volume reached ≈100 mm^3^ (Volume = 0.5 × Length × Width^[^ ^2^ ^]^), the mice were randomly divided into five groups (*n* = 5), and intravenously injected with saline, free JB, JB+Ce6+L, CJP–TiN, or CJP–TiN+L (Ce6 = 10 mg kg^−1^, JB = 40 mg kg^−1^). In the JB+Ce6+L and CJP–TiN+L groups, the mice received 660 nm laser irradiation for 5 min (100 mW cm^−2^) 6 h after treatment. The above treatments were conducted four times. The body weight and tumor volume were monitored, and on day 14, the tumors were excised and weighed. In addition, the heart, liver, spleen, and lungs were collected for H&E, Ki67, and PCNA staining, to evaluate the tumor suppression effects and potential organ damage.

### In Vivo Study of the Abscopal Effect of CJP–TiN

To evaluate the distant effects of CJP–TiN in a mouse MC model, the subcutaneous GC model was employed in immunocompetent 615 mice. Initially, MFC cells (1 × 10^7^/100 µL) were injected subcutaneously into the right dorsal regions of male 615 mice (18–20 g) to establish a primary tumor model. After 6 d, an identical injection was performed into the left flanks to create a secondary tumor model. When the primary tumor volume reached 50 mm^3^, the mice were randomly assigned to three groups (*n* = 5), i.e., the Saline, CJP–TiN, and CJP–TiN+L groups. After 6 h, CJP–TiN injection was performed (Ce6 = 10 mg kg^−1^), and the primary tumor site was subjected to 660 nm laser irradiation for 5 min (100 mW cm^−2^). On day 21, tumor tissues from both sides were collected for H&E, Ki67, and PCNA staining to further evaluate the tumor suppression effects. In addition, for the mouse GC model, tumor tissues were collected, cut into small fragments, and incubated in RPMI 1640 medium containing collagenase (1 mg mL^−1^) and DNase (10 µg mL^−1^) at 37 °C for 1 h. The resulting cell suspension was filtered, centrifuged, and washed with PBS containing 1% FBS. ACK lysis buffer was then used to remove the red blood cells, and the single‐cell suspension was washed again with PBS containing 1% FBS, followed by FCM analysis.

### Combination of α‐PD1 Immunotherapy with CJP–TiN

To assess the anti‐tumor synergy between CJP–TiN and anti‐PD1 immunotherapy, the subcutaneous GC model was employed in immunocompetent 615 mice. When the volume of the right tumor reached 100 mm^3^, the mice were randomly divided into four groups (*n* = 5), namely the Saline, CJP‐TiN+L, α‐PD1 (Bio X Cell, USA), and CJP‐TiN+L+α‐PD1 groups. α‐PD1 was administered intraperitoneally at 10 mg kg^−1^, while CJP–TiN was given at a dose of 10 mg kg^−1^ along with 660 nm laser irradiation for 5 min (100 mW cm^−2^). The tumor volume and body weight were recorded every 2 d, and a tumor volume > 2000 mm^3^ was considered indicative of death.

### Statistical Analysis

All data were expressed as the mean ± SEM from at least three independent experiments. Statistical analysis was performed using independent sample *t*‐tests, with graphical representation created using GraphPad Prism version 10. Statistical significance was defined as follows: ^*^ *p* < 0.05, ^**^ *p* < 0.01, ^***^ *p* < 0.001, and ^****^ *p* < 0.0001, ns indicated no statistical significance. Final figure formatting was done using Adobe Illustrator 2021.

## Conflict of Interest

The authors declare no conflict of interest.

## Supporting information



Supporting Information

Supporting Information

Supporting Information

## Data Availability

The data that support the findings of this study are available in the supplementary material of this article.
