# Supplementary material for: Targeted Dual‐Responsive Liposomes Co‐Deliver Jolkinolide B and Ce6 to Synergistically Enhance the Photodynamic/Immunotherapy Efficacy in Gastric Cancer through the PANoptosis Pathway
Source: Adv Sci (Weinh). 2025 May 19;12(29):e02289. doi: 10.1002/advs.202502289 (PMC12362734; doi:10.1002/advs.202502289)
Supplement: Supplementary file 1 — Supporting Information [file ADVS-12-e02289-s001.docx]

**Targeted Dual-Responsive Liposomes Co-deliver Jolkinolide B and Ce6 to Synergistically Enhance the Photodynamic/Immunotherapy Efficacy in Gastric Cancer through the PANoptosis Pathway**

Chenhui Ma^1*^, Lei Gao^1*^, Kewei Song^1^, Baohong Gu^1^, Bofang Wang^1^, Yang Yu^2^, Xueyan Wang^1^, Xuemei Li^3^, Jike Hu^1^, Weigao Pu^1^, Yunpeng Wang^1^, Na Wang^4^, Dedai Lu^5^, Zhijian Han^3^, Hao Chen^3,6 #^

1. The Second Clinical Medical College, Lanzhou University, Lanzhou, China.

2. Department of Thyroid Surgery, Second Affiliated Hospital, Zhejiang University School of Medicine, Hangzhou, China.

3. Gansu Provincial Key Laboratory of Environmental Oncology, Lanzhou, Gansu, China.

4. National Institute for Data Science in Health and Medicine, Xiamen University, Xiamen, China.

5. Key Laboratory of Eco-Functional Polymer Materials of the Ministry of Education, Northwest Normal University, Lanzhou, China.

6. Department of Tumor Surgery, Lanzhou University Second Hospital, Lanzhou, China.

*These authors contributed equally to this work.

^#^**Corresponding author**: Hao Chen, No. 82, Cuiyingmen, Chengguan District, Lanzhou city, Gansu Province; Email (ery_chenh@lzu.edu.cn).

**Table S1.** Molecular docking site.

|  | Binding energy(Kcal/mol) | Hydrogen Bonds | Hydrophobic Interaction | Salt Bridges |
| --- | --- | --- | --- | --- |
| iRGD-α_v_β_3_ | -8.2 | Ser 121B (3.41 Å),  Tyr 122B (3.90 Å),  Ser 123B (3.57 Å),  Tyr 166B (3.74 Å),  Tyr 178A (3.50 Å), Gln 180A (3.92 Å), Arg 214B (2.91 Å), Asn 215B (3.69 Å), Asp 218A (3.03 Å), Ala 218B (3.55 Å), Asp 251B (3.04 Å) | Tyr 122B (3.21 Å), Tyr 178A (3.42 Å, 3.80 Å) | Glu 220B (3.57 Å) |

**Table S2. Kinetic parameters of iRGD binding to αvβ3 integrin measured by SPR**

| Ligand | Ligand Conc. | Level (RU) | Analyte | Analyte Conc. | ka(1/Ms) | kd(1/s) | KD(M) | Chi²(RU²) |
| --- | --- | --- | --- | --- | --- | --- | --- | --- |
| αvβ3 | 20 μg/mL | 12600 | iRGD | (0.02-1) μM | 8.30e+04 | 3.21e-03 | 3.87e-08 | 0.98 |

**Table S3. Alanine mutation analysis**

| Mutation | dAffinity(kcal/mol) |
| --- | --- |
| 1:D150A | 0.0978 |
| 1:D218A | 0.4111 |
| 1:D219A | -0.0957 |
| 1:F177A | 0.0037 |
| 1:I216A | 0.0067 |
| 1:Q180A | -0.0173 |
| 1:Q214A | -0.0036 |
| 1:T212A | 0.0192 |
| 1:W179A | -0.1058 |
| 1:Y178A | 0.6619 |
| 2:D217A | 0.0471 |
| 2:D251A | 0.2191 |
| 2:E220A | 0.5305 |
| 2:K253A | 0.1222 |
| 2:M180A | 0.2369 |
| 2:M335A | -0.0043 |
| 2:N215A | 0.2125 |
| 2:N313A | 0.2078 |
| 2:P219A | -0.0147 |
| 2:R214A | 0.3209 |
| 2:R216A | 0.0648 |
| 2:S121A | 0.0248 |
| 2:S123A | 0.3213 |
| 2:T311A | -0.0303 |
| 2:V314A | 0.0975 |
| 2:Y122A | 0.0548 |
| 2:Y166A | 0.1478 |

**Table S4.** Encapsulation efficiency and drug loading.

|  | Ce6 Drug loading% | JB Drug loading% | Ce6 Encapsulation efficiency% | JB Encapsulation efficiency% |
| --- | --- | --- | --- | --- |
| 5% | 1.89±0.14 | 2.38±0.37 | 75.60±1.5 | 95.20±4.72 |
| 10% | 4.12±0.16 | 4.66±0.22 | 82.40±3.89 | 93.20±2.08 |
| 20% | 4.23±0.26 | 2.01±0.05 | 42.30±1.56 | 20.10±0.6 |
| 30% | 4.910.13 | 2.98±0.02 | 32.70±2.82 | 19.80±1.39 |

**Table S5.** Zeta potential and Particle diameters, PDI and Zeta potential.

|  | Hydrodynamic size (nm) | PDI | Zeta potential (mV) |
| --- | --- | --- | --- |
| 1st | 112.56 | 0.231 | -31.05 |
| 2nd | 106.08 | 0.225 | -30.38 |
| 3rd | 109.45 | 0.229 | -33.31 |
| Average | 109.36±3.2 | 0.228±0.003 | -31.58±1.73 |

**Table** **S6**. Information of antibodies assays.

| **Protein targets** | **Assays** | **Host** | **Company** | **product code** | **dilution ratio** |
| --- | --- | --- | --- | --- | --- |
| Primary antibodies |  |  |  |  |  |
| β-actin | WB | Mouse | Proteintech | #60008-1-Ig | 1:5000 |
| αvβ3 | WB  IHC | Rabbit | Bioss | #bs-1310R | 1:200  1:500 |
| Cleaved Caspase-3 | WB | Rabbit | Cell Signaling Technology | #9664 | 1:1000 |
| Cleaved Caspase-7 | WB | Rabbit | Cell Signaling Technology | #8438 | 1:1000 |
| Cleaved Caspase-8 | WB | Rabbit | Cell Signaling Technology | #9496 | 1:1000 |
| Bax | WB | Rabbit | Proteintech | #50599-2-Ig | 1:2000 |
| Bcl-2 | WB | Rabbit | Proteintech | #12789-1-AP | 1:2000 |
| p-MLKL | WB | Mouse | Abcam | #ab196436 | 1:1000 |
| p-RIPK1 | WB | Rabbit | Proteintech | #28252-1-AP | 1:2000 |
| Cleaved gasdermin E | WB | Rabbit | Cell Signaling Technology | #55879 | 1:1000 |
| HMGB1 | WB, IF | Rabbit | Proteintech | #10829-1-AP | 1:2000 |
| Ki67 | IHC | Mouse | Servicebio | #GB121141-100 | 1: 300 |
| PCNA | IHC | Mouse | Servicebio | #GB12010-100 | 1: 500 |
| CD8 | IF | Rabbit | Abcam | #ab217344 | 1: 300 |
| CXCL10 | IF | Rabbit | Bioss | #bs-1502R | 1: 500 |
| FITC anti-CD3 | FC | / | MULTI SCIENCES | #AH00301-20 | / |
| PerCP anti-CD4 | FC | / | MULTI SCIENCES | #AH00407-20 | / |
| APC anti-CD8 | FC | / | MULTI SCIENCES | #AH008A0411-20 | / |
| ATP | ELISA | / | invitrogen | #A22066 | / |
| IL-18 | ELISA | / | Lianke Biotech | #EK118 | / |
| IL-1β | ELISA | / | Lianke Biotech | #EK101B | / |
| Annexin V-FITC/PI Cell Apoptosis Detection Kit | FC | / | Servicebio | #G1511-50T | / |
| Cell Activation Cocktail (with Brefeldin A) | FC | / | BioLegend | #423303 | / |
| Zombie Aqua™ Fixable Viability Kit | FC | / | BioLegend | #423101 | / |
| APC-Cy7 CD45- anti-mouse | FC | / | BioLegend | #103115 | / |
| FITC anti-mouse CD3ε | FC | / | BioLegend | #100306 | / |
| PE/Cyanine7 anti-mouse CD8a | FC | / | BioLegend | #100721 | / |
| PE Granzyme B | FC | / | BioLegend | #372208 | / |
| APC Perforin anti-mouse | FC | / | BioLegend | #154304 | / |
| CD4 | FC | / | BioLegend | #100539 | / |
| APC anti-mouse CD25 | FC | / | BioLegend | #102011 | / |
| PE anti-mouse FOXP3 | FC | / | BioLegend | #320008 | / |
| FITC anti-mouse/human CD11b | FC | / | BioLegend | #101205 | / |
| APC/Cyanine7 anti-mouse F4/80 | FC | / | BioLegend | #123118 | / |
| PerCP/Cyanine5.5 anti-mouse CD86 | FC | / | BioLegend | #105028 | / |
| PE anti-mouse CD206 (MMR) | FC | / | BioLegend | #141706 | / |
| **Secondary antibodies** |  |  |  |  |  |
| Goat anti-rabbit IgG | WB | Rabbit | Proteintech | #SA00001-2 | 1:8000 |
| Goat anti-mouse IgG | WB | Mouse | Proteintech | #SA00001-1 | 1:8000 |
| CoraLite488-conjugated Goat Anti-Mouse IgG(H+L) | IF | Mouse | Proteintech | #SA00013-1 | 1:500 |
| CoraLite594-conjugated Goat Anti-Rabbit IgG(H+L) | IF | Rabbit | Proteintech | #SA00013-4 | 1:500 |
| CoraLite647-conjugated Mouse Anti-Heavy Chain of Rabbit IgG | IF | Mouse | Proteintech | #SA00014-6 | 1:500 |

**Supplementary Figures**

**
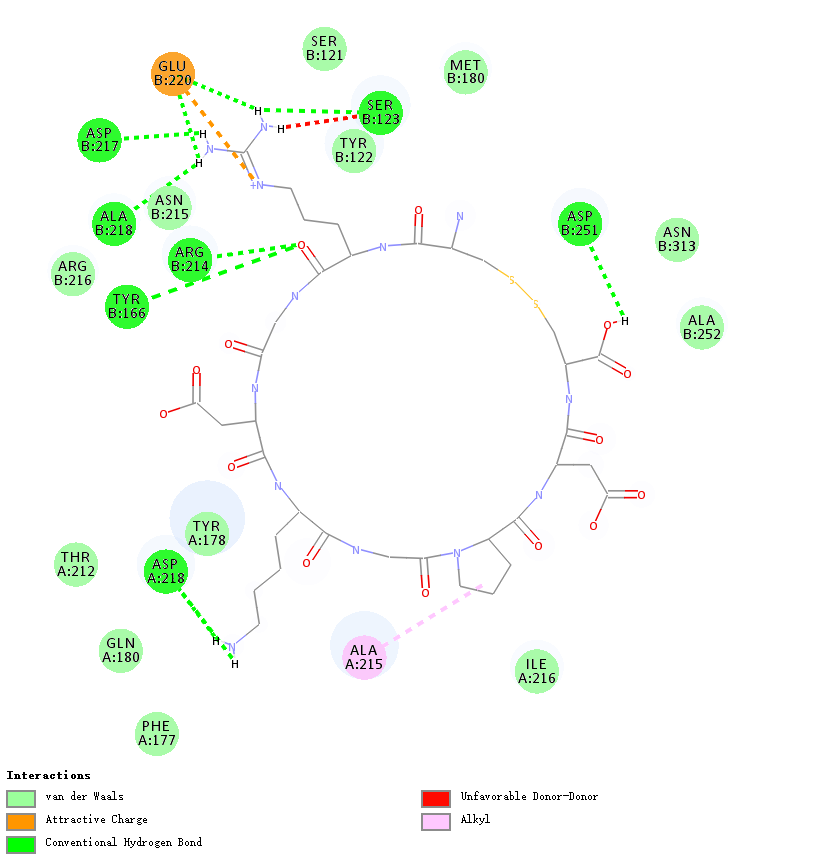
**

**Figure S1. Molecular docking interactions between the CJP-TiN ligand (iRGD) and the α_v_β_3_.** The figure shows key amino acid residues involved in interactions, with distances (in Å) indicated by green dashed lines.


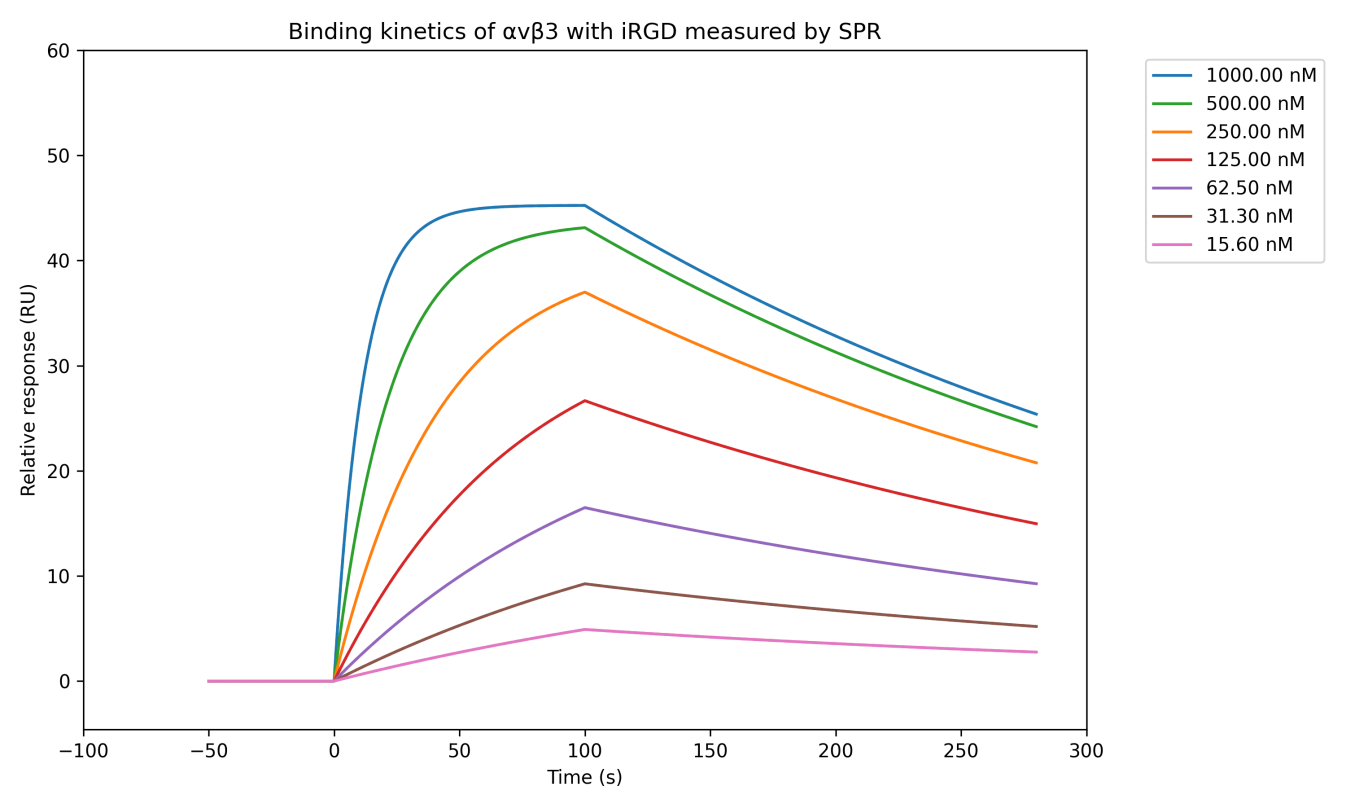


**Figure S2. Binding kinetics of αvβ3 with iRGD measured by SPR.** αvβ3 immobilized on a CM5 chip can bind iRGD with an affinity constant of 3.87e-08 M as determined in a SPR assay.


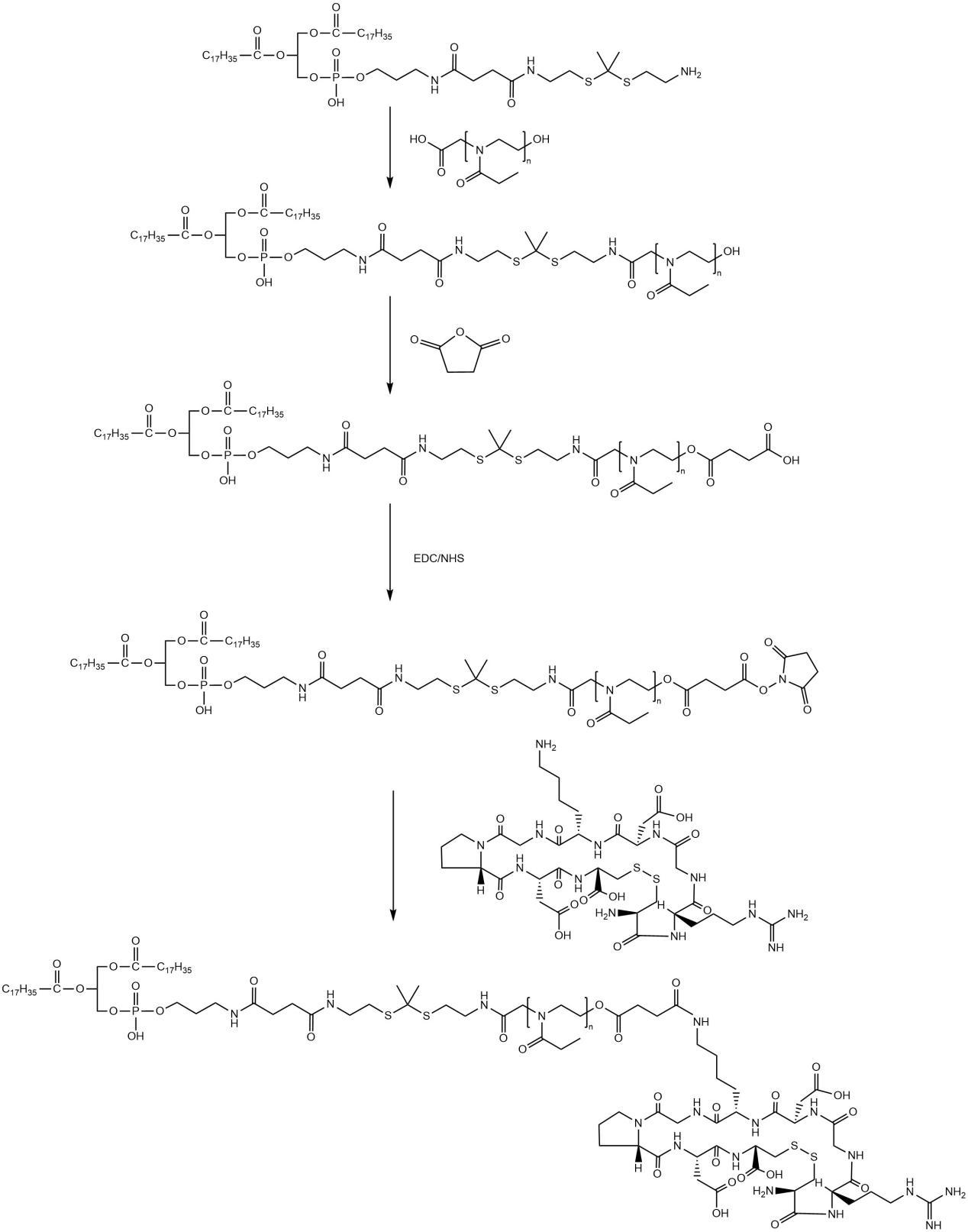


**Figure S3. Synthesis of DSPE-TK-PEOz_2k_-iRGD**


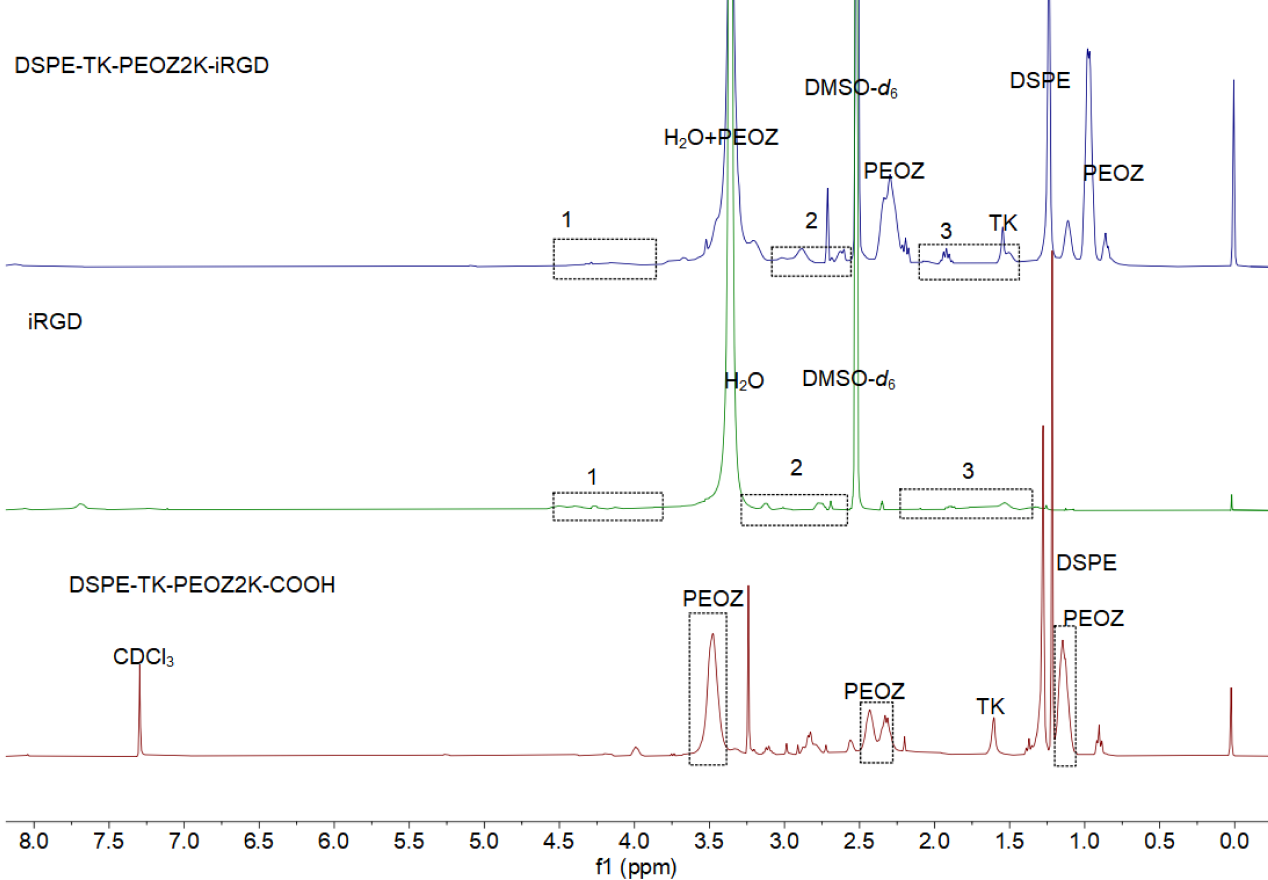


**Figure S4. The ^1^H NMR spectra of DSPE-TK-PEOz_2K_-iRGD (blue), iRGD (green), and DSPE-TK-PEOz_2K_-COOH (red) are shown.** Characteristic peaks corresponding to iRGD are labeled as 1, 2, and 3, representing key proton signals of the iRGD peptide. Peaks from the PEOz2K and DSPE components are also observed, confirming successful synthesis of the DSPE-TK-PEOz_2K_-iRGD conjugate. The presence of these peaks in the blue spectrum (DSPE-TK-PEOz_2K_-iRGD) validates the successful conjugation of iRGD to the DSPE-TK-PEOz_2K_ structure.


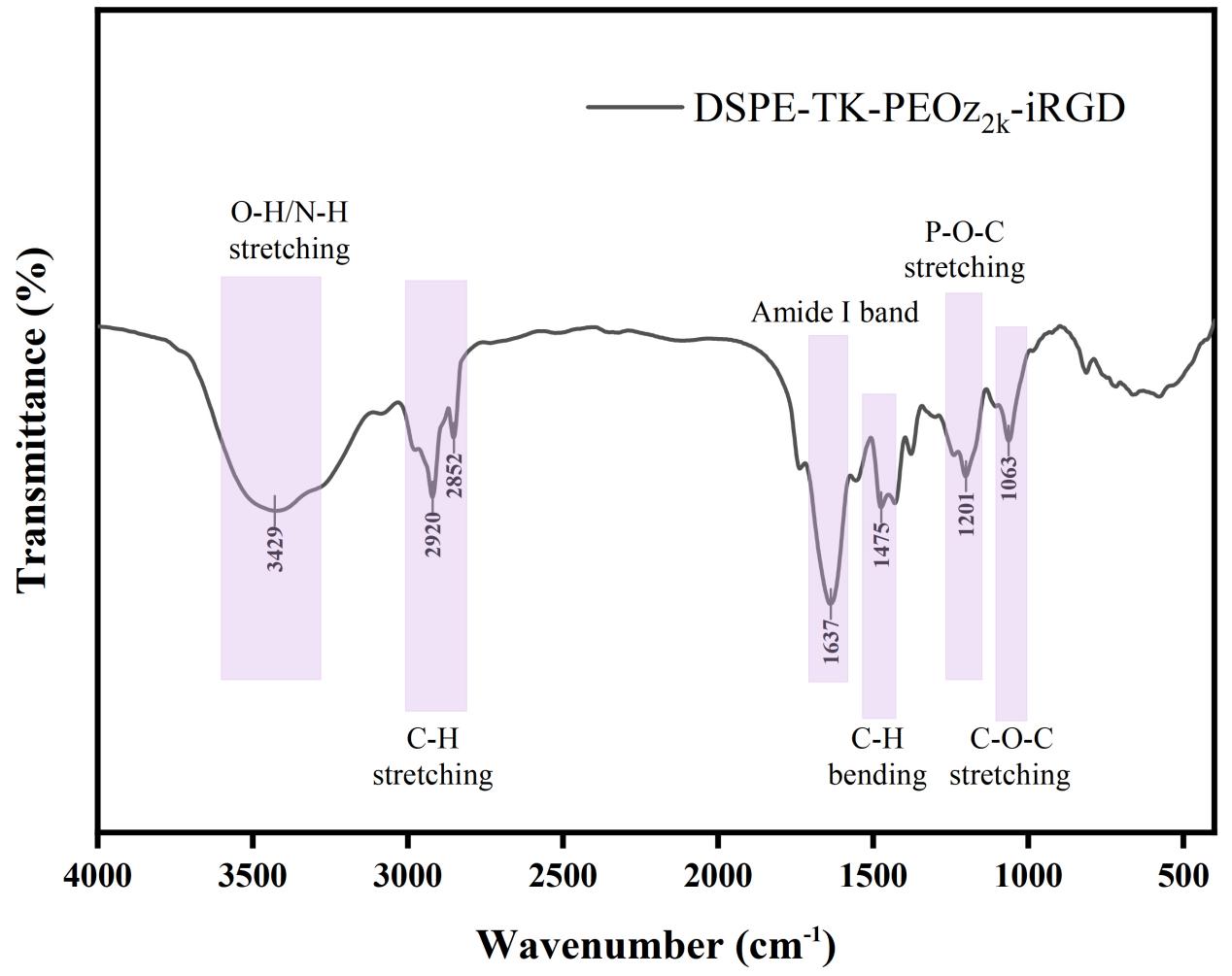


**Figure S5. FTIR spectrum of DSPE-TK-PEOz_2k_-iRGD**

As shown in the figure, FTIR analysis of the DSPE-TK-PEOz_2k_-iRGD sample shows a broad peak at 3429 cm^-1^, corresponding to the N-H stretching vibration and partial O-H vibration in the amide bond. The strong peaks at 2920 cm^-1^ and 2852 cm^-1^ correspond to the asymmetric and symmetric stretching vibrations of CH_2_ and CH_3_ in the lipid chains, respectively, consistent with the hydrophobic alkyl chains in DSPE. The peak at 1637 cm^-1^ corresponds to the C=O stretching vibration of the amide I band, indicating that the iRGD peptide forms an amide bond with DSPE-TK-PEOz_2k_-NHS via the amine group. The peak at 1475 cm^-1^ arises from the CH_2_ scissoring bending vibration in the lipid chain, further confirming the DSPE hydrophobic tail. Peaks at 1201 cm^-1^ and 1063 cm^-1^ correspond to the stretching vibrations of the phosphodiester bond (P-O-C) and the ether bond (C-O-C) in PEOz, consistent with the structural features of the DSPE head and the PEOz block. The C=O stretching peak (~1730 cm^-1^) of the NHS active ester in the reactant DSPE-TK-PEOz_2k_-NHS is absent in the product spectrum, indicating complete participation of the active ester in the amidation reaction. The presence of the amide I band (1637 cm^-1^) and N-H vibration peak (3429 cm^-1^) confirms successful covalent attachment of the iRGD peptide to the carrier molecule.

**Figure S6. Enlarged MALDI-TOF spectrum of DSPE-TK-PEOz-iRGD highlighting the periodic mass intervals, corresponding to the repeating units within the polymer backbone.**

The sample data were collected using a Bruker MALDI-TOF mass spectrometer, and the analysis was performed using the PolyTools software. The mass spectrum of DSPE-TK-PEOz-iRGD is shown in the figure S6. A series of polymer ion peaks was detected from the sample, successfully characterizing the polymer units with a mass difference of 99.1 Da (C5H9NO, PEOz, 99.1). Since PEOz is a polymer ^[1]^, it is consistent with the observations from the mass spectrometry analysis. The measured molecular weight is as follows: Mw = 3606.05, Mn = 3428.49, and the polydispersity index is 1.05179.

[1] Bauer M, Lautenschlaeger C, Kempe K, Tauhardt L, Schubert US, Fischer D. Poly(2-ethyl-2-oxazoline) as alternative for the stealth polymer poly(ethylene glycol): comparison of in vitro cytotoxicity and hemocompatibility. Macromol Biosci. 2012;12(7):986-998.

_
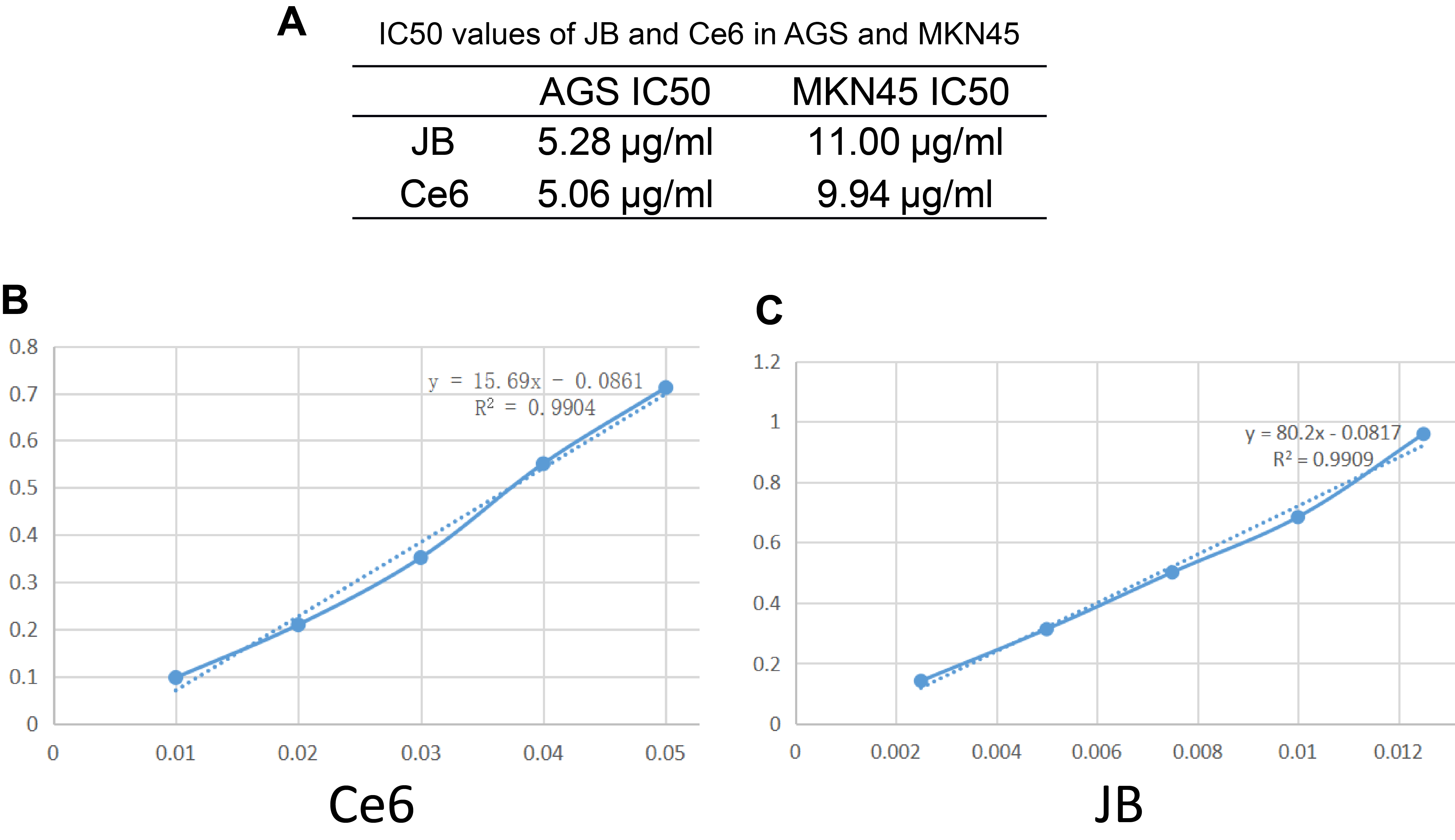
_

**Figure S7. IC_50_ and standard curves of Ce6 and JB in AGS and MKN45 cells.** (A) IC50 values of Ce6 and JB in AGS and MKN45 cells. The IC50 of Ce6 is 5.06 μg/ml in AGS cells and 9.94 μg/ml in MKN45 cells. The IC50 of JB is 5.28 μg/ml in AGS cells and 11.00 μg/ml in MKN45 cells, showing a concentration-dependent reduction in cell viability. (B) The standard curve of Ce6, showing a linear relationship within the experimental concentration range (R^2^ = 0.9904). (C) The standard curve of JB, showing a linear relationship within the experimental concentration range (R^2^ = 0.9909).


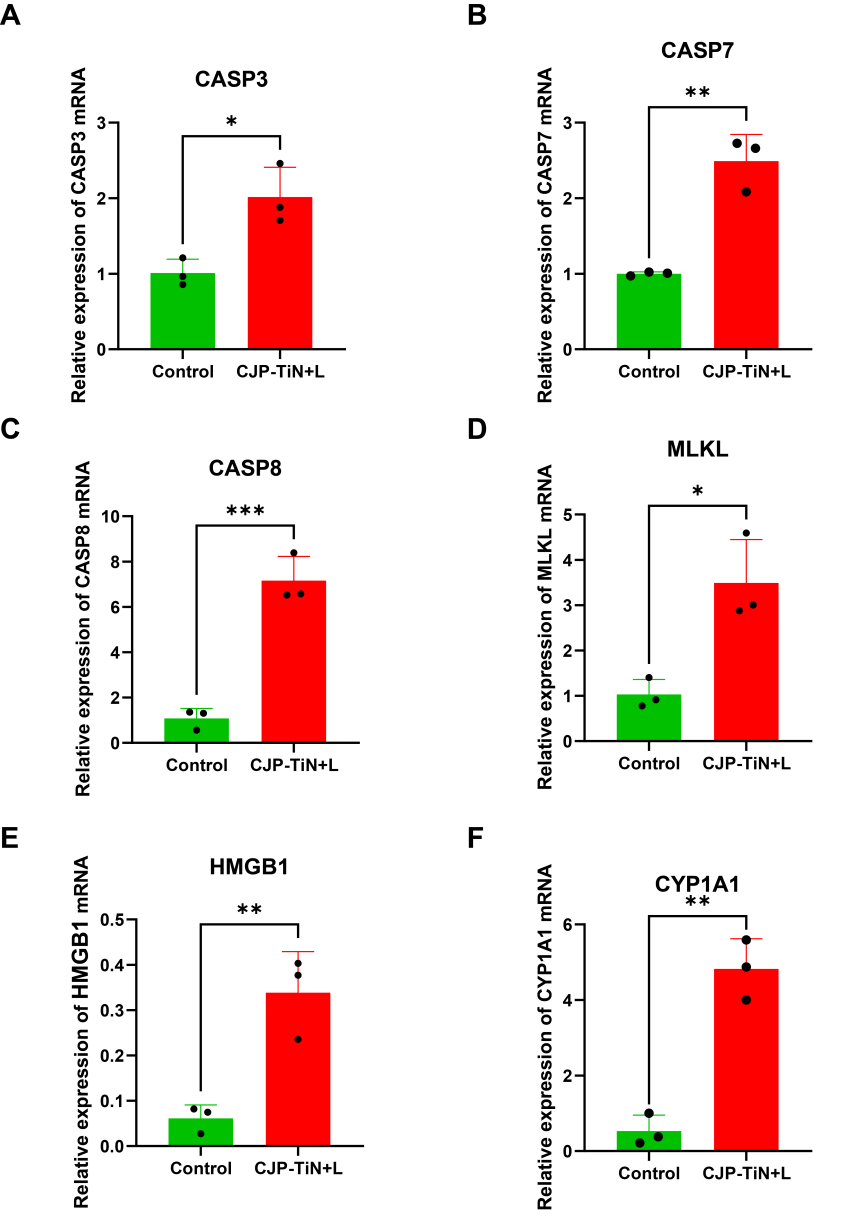


**Figure S8. mRNA expression analysis of key genes in response to CJP-TiN+L treatment.** Relative mRNA expression of CASP3 (A), CASP7 (B), CASP8 (C), MLKL (D), HMGB1 (E), and CYP1A1 (F) in control and CJP-TiN+L treated cells. Treatment with CJP-TiN+L significantly increased the expression of all tested genes compared to the control. The data are presented as mean ± SEM (n=3). Statistical significance was determined by Student's t-test (* *p* < 0.05, ** *p* < 0.01, *** *p* < 0.001).


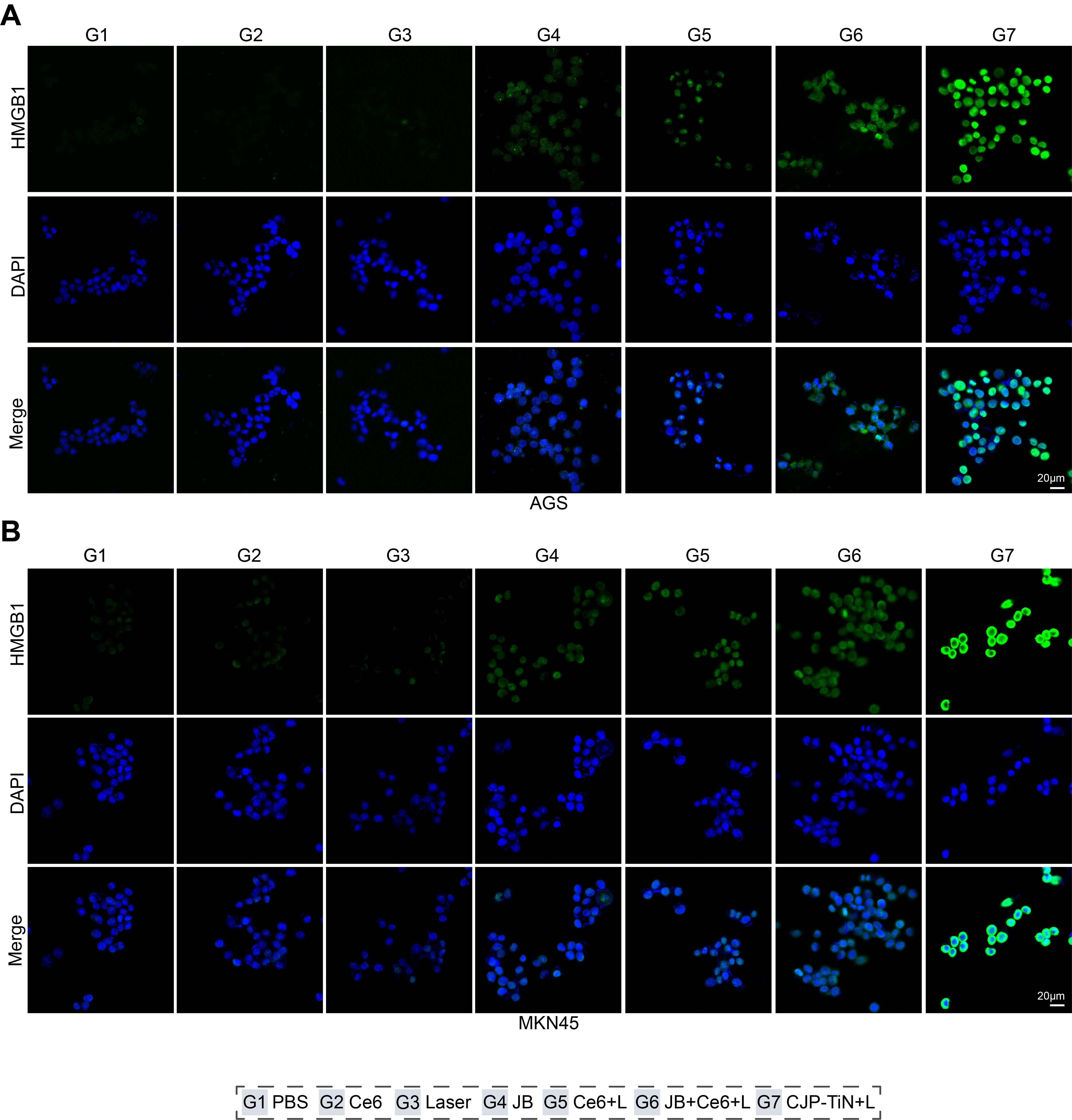


**Figure S9. Confocal imaging of HMGB1 expression in AGS and MKN45 cells.** (A) AGS cells were treated with various conditions (G1-G7) and stained for HMGB1 (green), DAPI (blue), and the merged image. G1: PBS, G2: Ce6, G3: Laser, G4: JB, G5: Ce6+L, G6: JB+Ce6+L, G7: CJP-TiN+L. The images show distinct HMGB1 staining in the cells under different treatment conditions. (B) MKN45 cells were treated similarly, and immunofluorescent staining was performed for HMGB1 (green) and DAPI (blue), with the merged image shown. The treatment groups are labeled as in (A). Both panels show that CJP-TiN+L treatment significantly induces HMGB1 expression in both AGS and MKN45 cells. Scale bar = 20 μm.


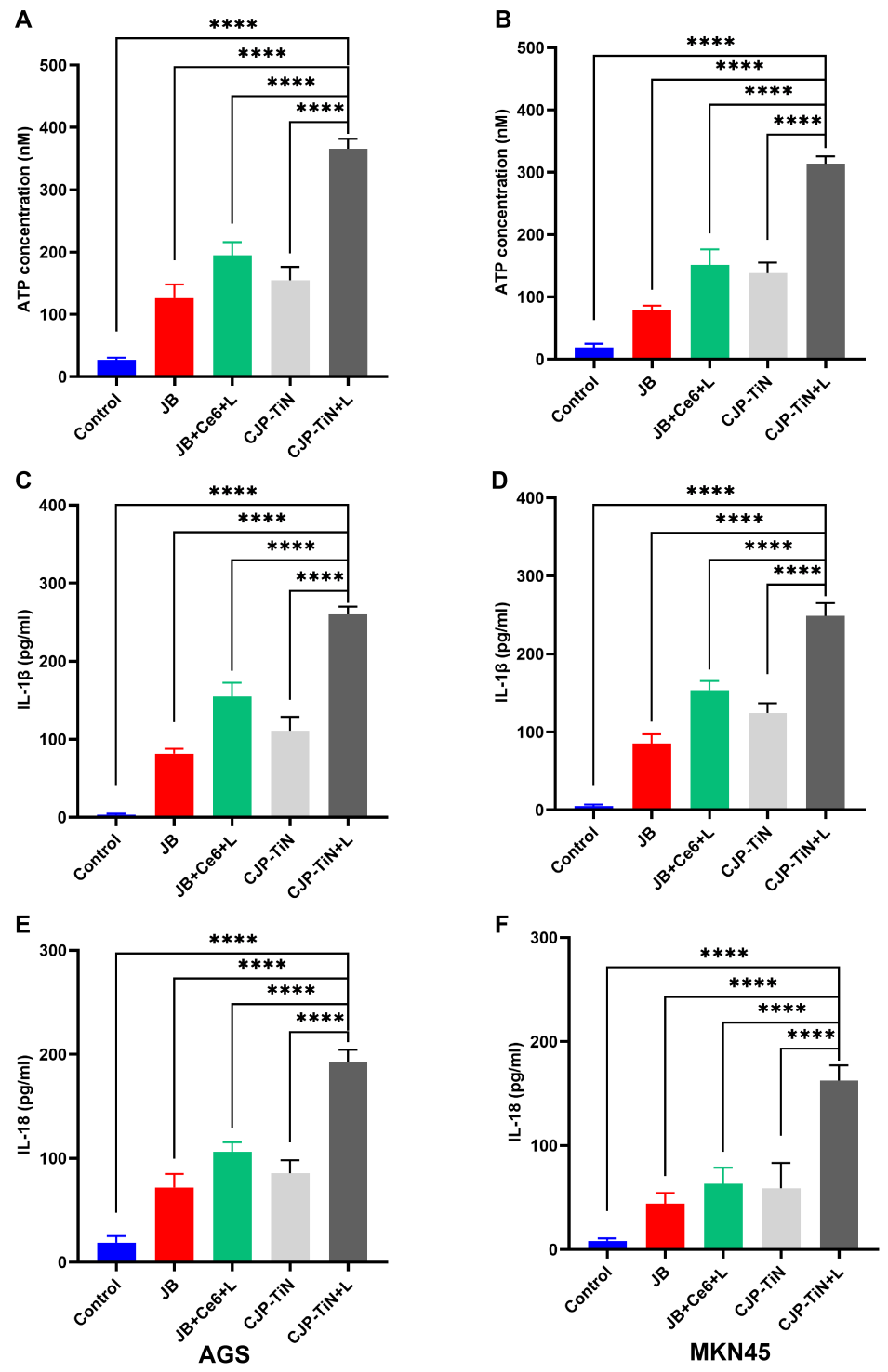


**Figure S10. Effects of different treatment groups on ATP concentration and inflammatory cytokine levels in AGS and MKN45 cells.** (A-B) show the changes in ATP concentration in AGS and MKN45 cells across different treatment groups. Compared to the control group, the JB, JB+Ce6+L, CJP-TiN, and CJP-TiN+L treatment groups significantly increased ATP concentration, with the CJP-TiN+L group showing the highest ATP levels (**** *p* < 0.0001). (C-D) show the concentration of IL-1β in each treatment group. All treatment groups had significantly higher IL-1β levels compared to the control group, with the CJP-TiN+L group having the highest concentration (**** *p* < 0.0001). (E) and (F) show the concentration of IL-18 in each treatment group. The results indicate that the JB, JB+Ce6+L, CJP-TiN, and CJP-TiN+L groups all significantly increased IL-18 levels, with the CJP-TiN+L group showing the most prominent effect (**** *p* < 0.0001). (mean ± SEM, n = 3).


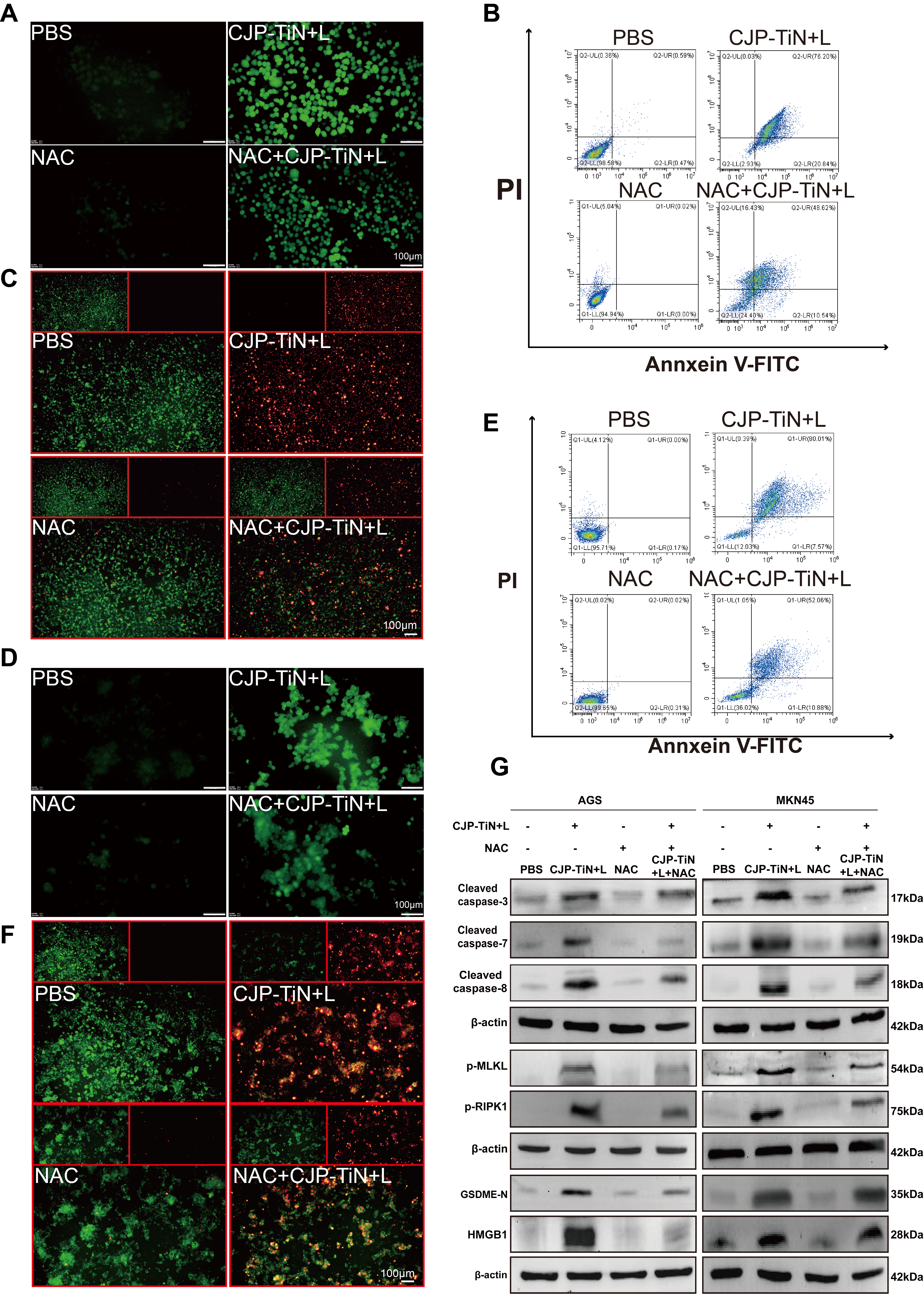


**Figure S11. Demonstrates that CJP-TiN induces cell death through ROS, and that NAC, as an ROS inhibitor, effectively attenuates CJP-TiN-induced cell death.** (A, D) Display the ROS generation (green) in AGS (A) and MKN45 (D) cells following treatment with PBS, CJP-TiN+L, and CJP-TiN+L combined with NAC. NAC treatment significantly reduces ROS generation induced by CJP-TiN. (B, E) Flow cytometry analysis of Annexin V-FITC and PI staining in AGS (B) and MKN45 (E) cells. NAC treatment reduces the proportion of apoptotic cells induced by CJP-TiN. (C, F) Representative fluorescence microscopy images showing PI staining (red) and cell viability (green) in AGS (C) and MKN45 (F) cells treated with PBS, CJP-TiN+L, and CJP-TiN+L combined with NAC. NAC treatment significantly reduces CJP-TiN-induced cell death. (G) Western blot analysis of cleaved caspase-3/7/8, p-MLKL, p-RIPK1, GSDME-N, and HMGB1 expression levels. CJP-TiN+L treatment activates PANoptosis pathways, while NAC inhibition suggests that ROS play a pivotal role in CJP-TiN-induced cell death. Scale bar = 100μm.


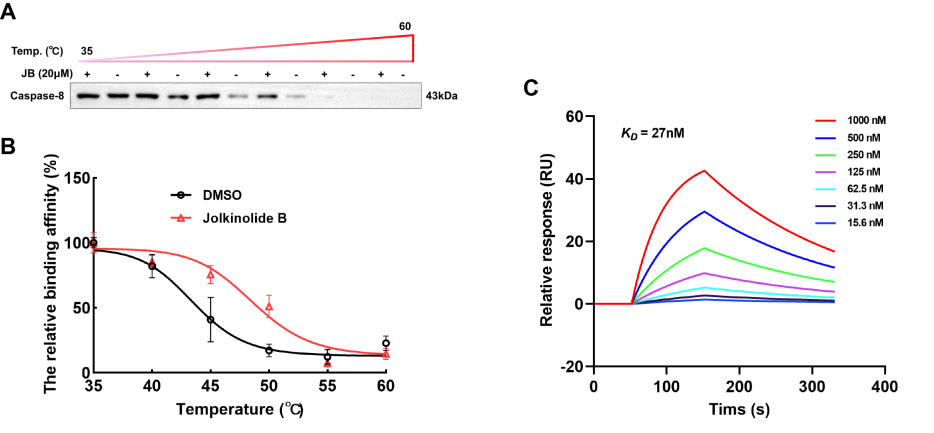


**Figure S12. CETSA and SPR analysis of Caspase-8 and JB. CETSA and SPR were employed to verify the direct interaction between JB and Caspase-8.** Experimental results (A-B) showed that JB significantly alleviated the impact of temperature variation on Caspase-8 stability, suggesting a direct interaction between JB and Caspase-8. Further surface plasmon resonance analysis (C) demonstrated that Caspase-8 immobilized on the CM5 chip could bind to JB, with an affinity constant of 27 nM, further supporting the specific binding between the two. These results indicate that JB exerts its biological effect through direct binding to Caspase-8, confirming their direct interaction while considering the actual impact of the cellular environment on the binding.

_
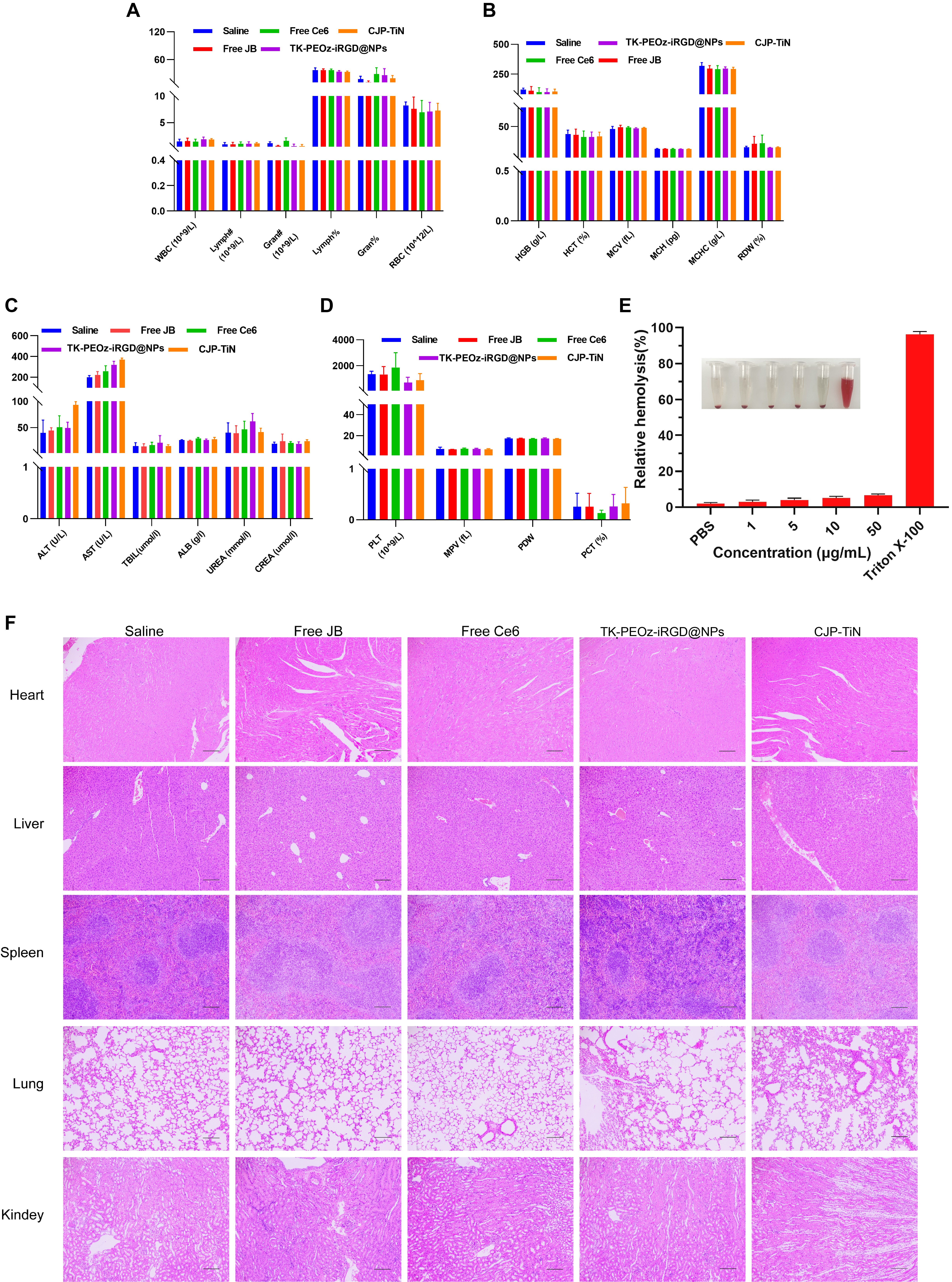
_

**Figure S13. Effects of different treatment groups on hematological parameters, liver and kidney function, coagulation function, and histopathology in mice.** (A) Effects of each treatment group on white blood cell (WBC), red blood cell (RBC), and hemoglobin (HGB) levels in mice. The results showed no significant differences among the groups. (B) Effects of each treatment group on platelet (PLT) count, neutrophil percentage (NE%), and lymphocyte percentage (LY%). No significant changes were observed among the groups. (C) Effects of each treatment group on liver function (ALT, AST, TBIL) and kidney function (UREA, CREA) in mice. No significant differences were observed among the groups. (D) Effects of each treatment group on coagulation function (PT, MPV, PDW, PCT). (E) Hemolysis test results at different concentrations for each treatment group. No significant hemolysis was observed in any group except the Triton X-100 group. (F) Histopathological examination of major organs (heart, liver, spleen, lung, kidney) in each treatment group (H&E staining). No obvious tissue damage or abnormal pathological changes were observed. Scale bar = 100μm.

_
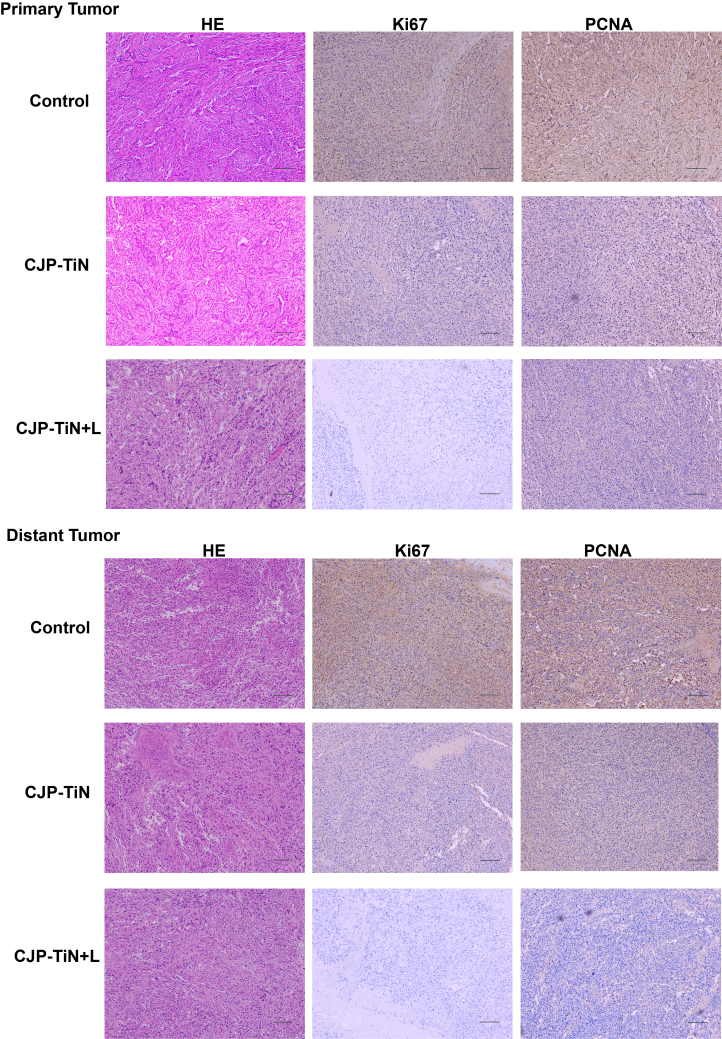
_

**Figure S14. Effects of CJP-TiN on proliferation markers in primary and distant tumor tissues.** In the primary tumor, CJP-TiN+L treatment significantly inhibited tumor cell proliferation in both primary and distant tumors, as evidenced by a marked reduction in the proportion of Ki67 and PCNA positive cells, along with disruption of tissue structure. In distant tumor tissues, the antitumor effect of the CJP-TiN+L group was the most significant compared to the CJP-TiN group. Scale bar = 100μm.

_
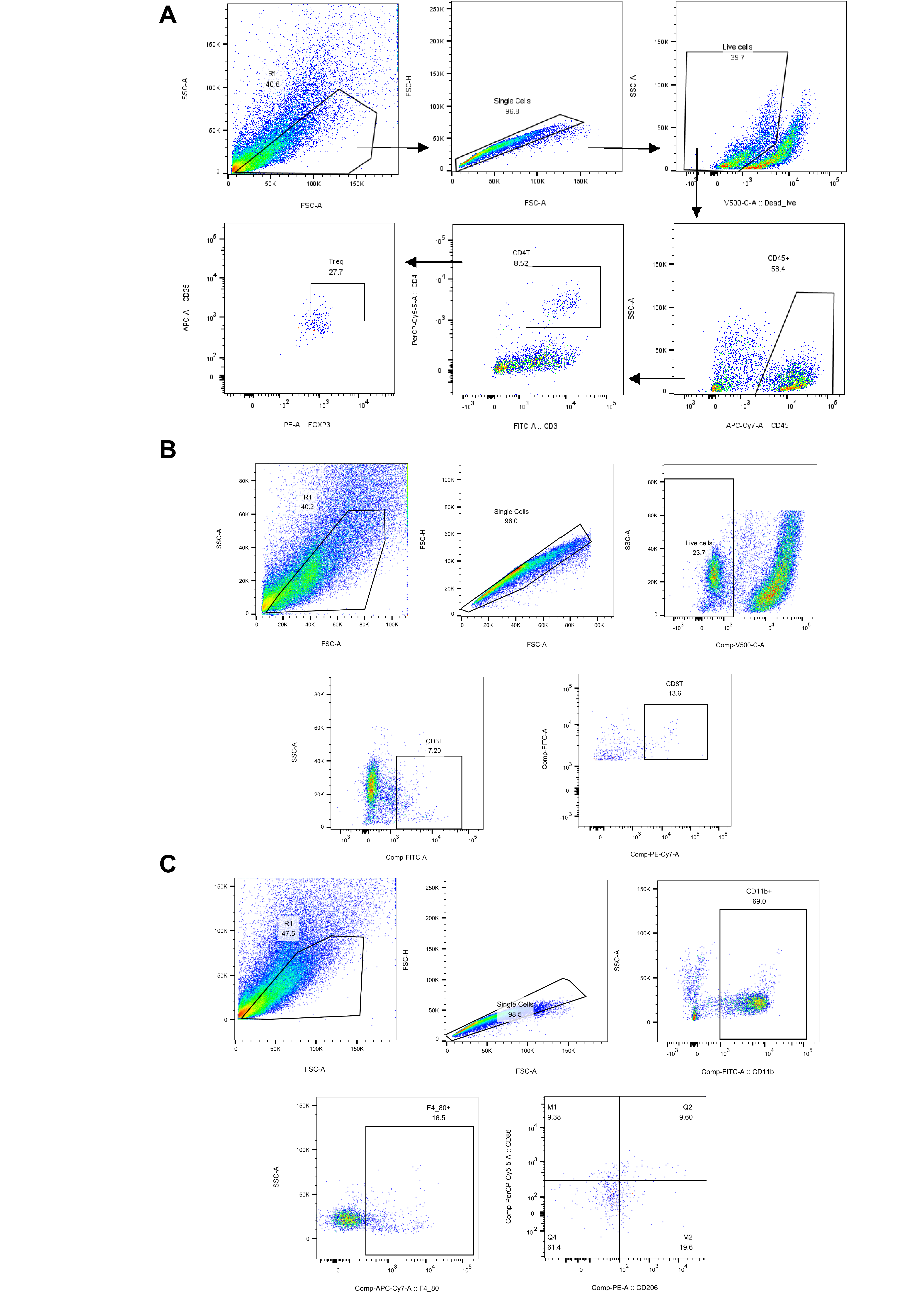
_

**Figure S15. Flow cytometry gating strategy.** (A) Flow cytometry analysis workflow for Treg and CD8+ T cells. The cell population was identified using SSC-A and FSC-A, followed by live/dead staining to distinguish live cells and further analyze the proportions of CD45+, CD4+, and FoxP3+ Treg cells. (B) This figure illustrates the gating strategy used in flow cytometry, starting from the exclusion of debris and doublets (R1), followed by the selection of single live cells, then CD3+ T cells, and finally identifying the CD8+ T cell population. (C) Analysis of myeloid-derived suppressor cell (MDSC) subsets. The total cell population was initially identified using SSC-A and FSC-A, followed by analysis of CD11b+ myeloid cells, and the proportions of different subsets were determined using CD86, CD206 and F4/80.

_
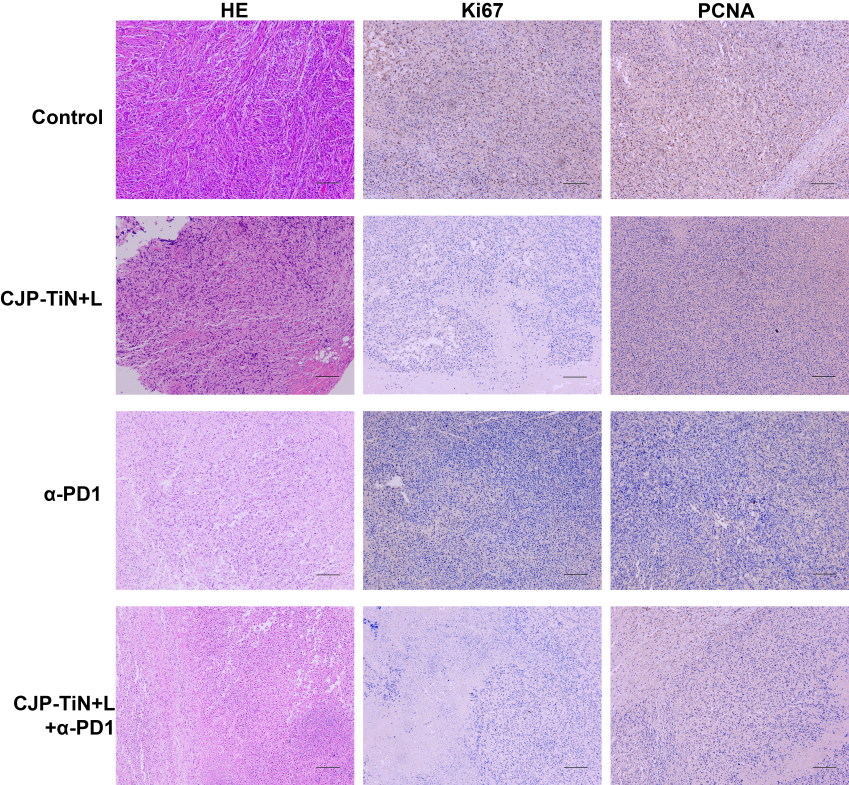
_

**Figure S16. Effects of different treatment groups on tumor proliferation markers (H&E, Ki67, and PCNA staining).** H&E staining showed significant necrosis and structural disruption in tumor tissues of the CJP-TiN+L group. Ki67 and PCNA staining results indicated that the CJP-TiN+L group had the lowest proportion of positive cells, demonstrating a significant inhibitory effect on proliferation. Scale bar = 100μm.
