# Supplementary material for: Targeted Dual‐Responsive Liposomes Co‐Deliver Jolkinolide B and Ce6 to Synergistically Enhance the Photodynamic/Immunotherapy Efficacy in Gastric Cancer through the PANoptosis Pathway
Source: Adv Sci (Weinh). 2025 May 19;12(29):e02289. doi: 10.1002/advs.202502289 (PMC12362734; doi:10.1002/advs.202502289)
Supplement: Supplementary file 2 — Supporting Information [file ADVS-12-e02289-s002.pptx]

## Slide 1
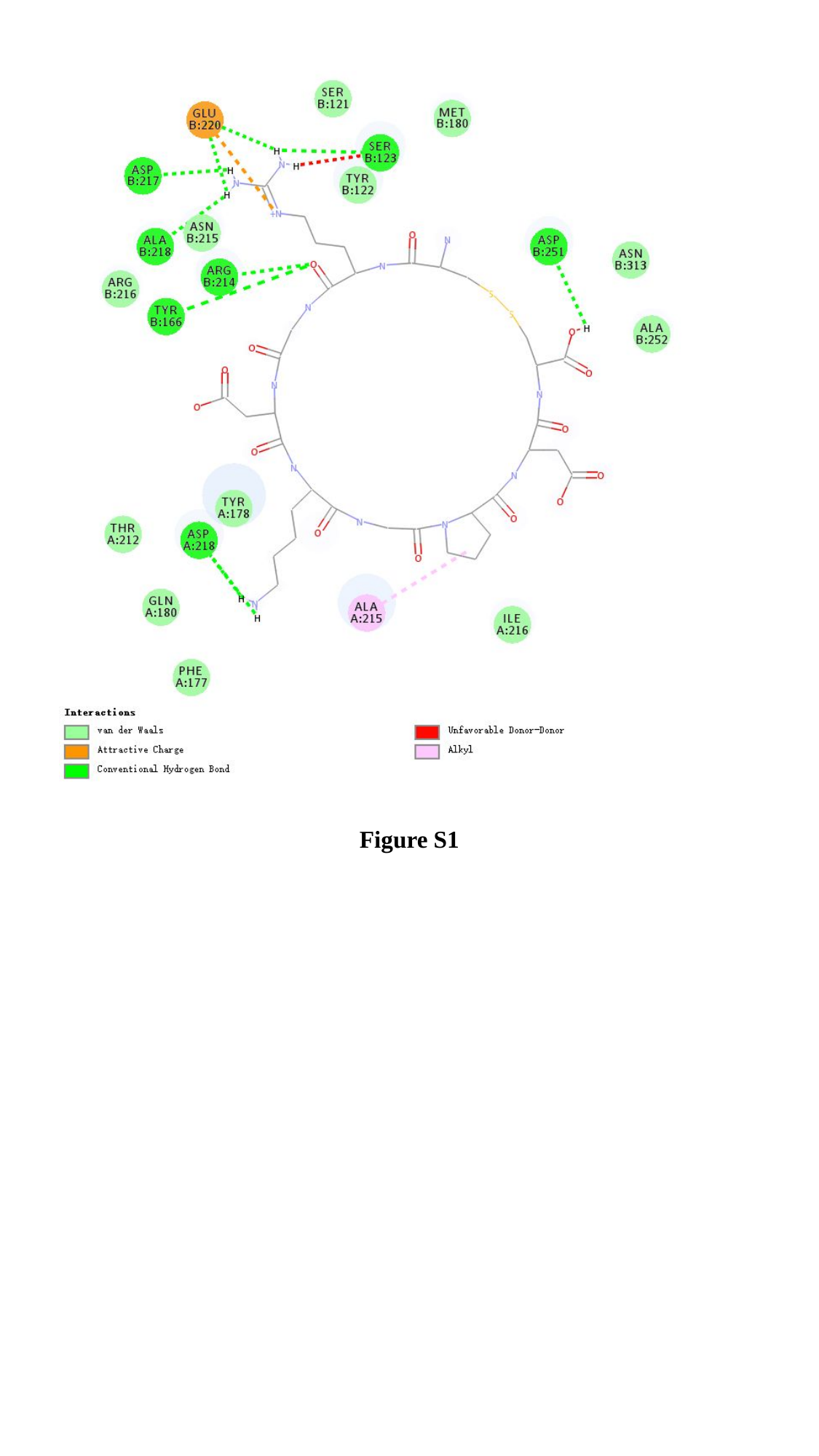

Figure S1

## Slide 2
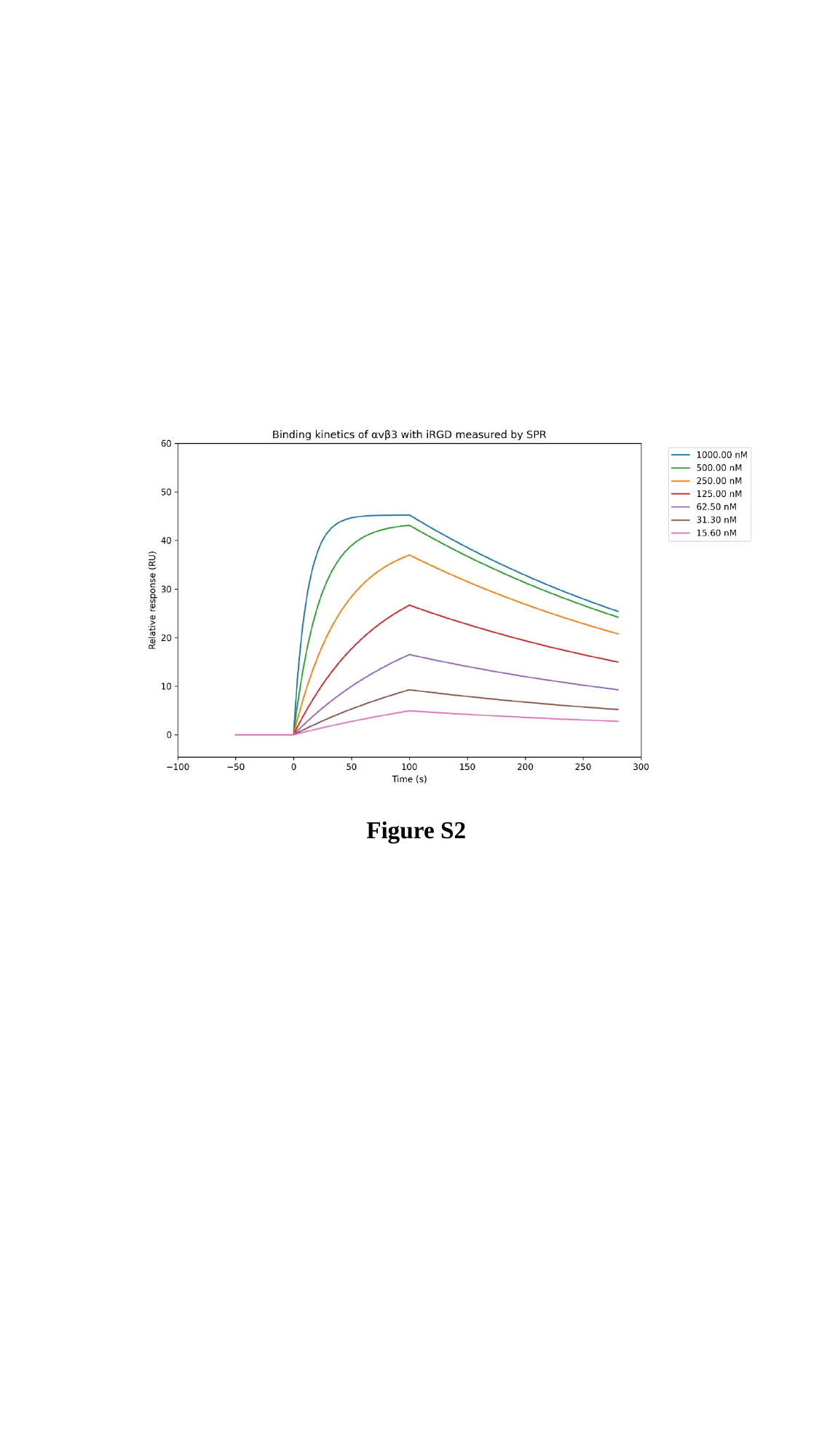

Figure S2

## Slide 3
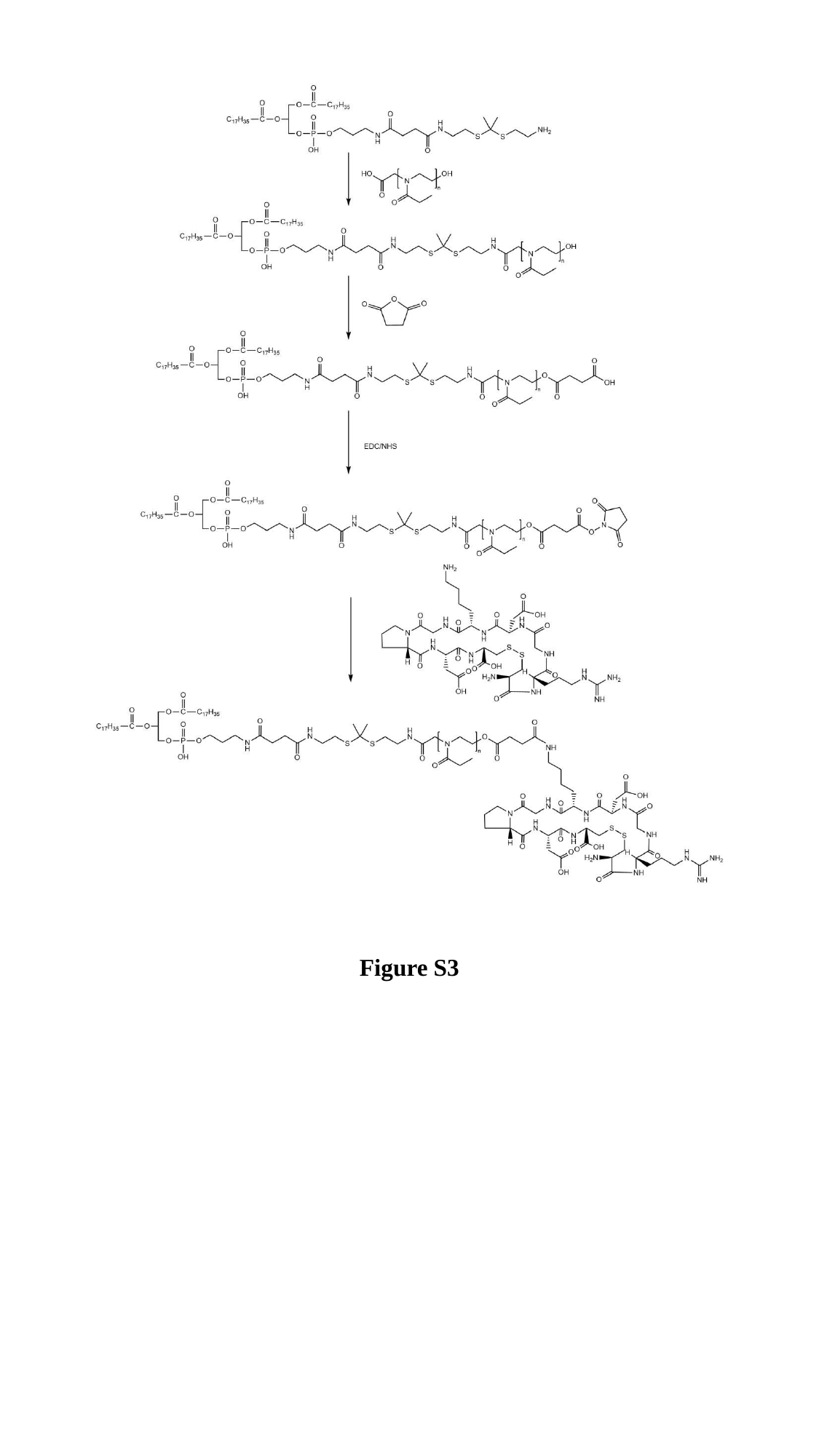

Figure S3

## Slide 4
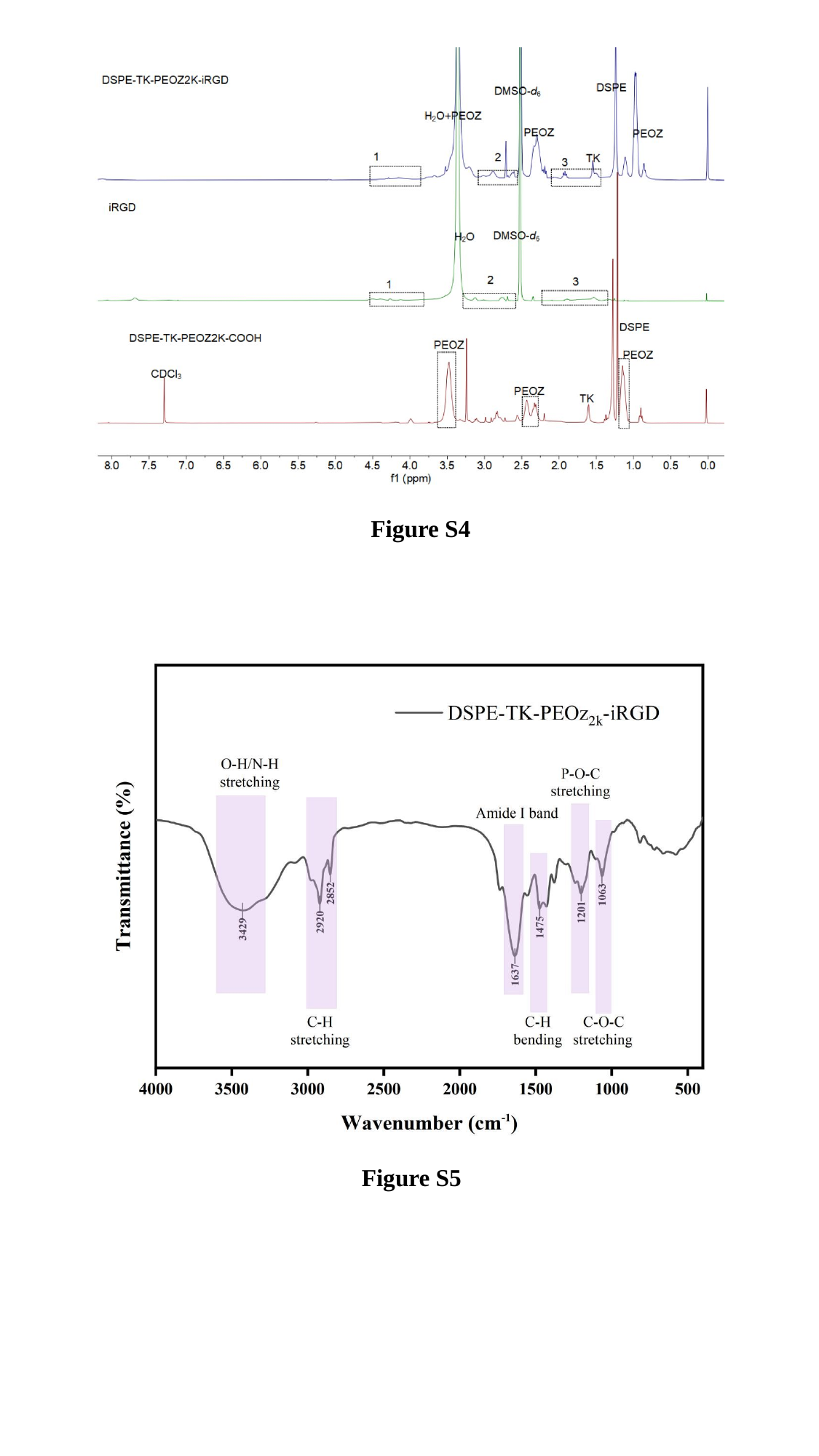

Figure S4
Figure S5

## Slide 5
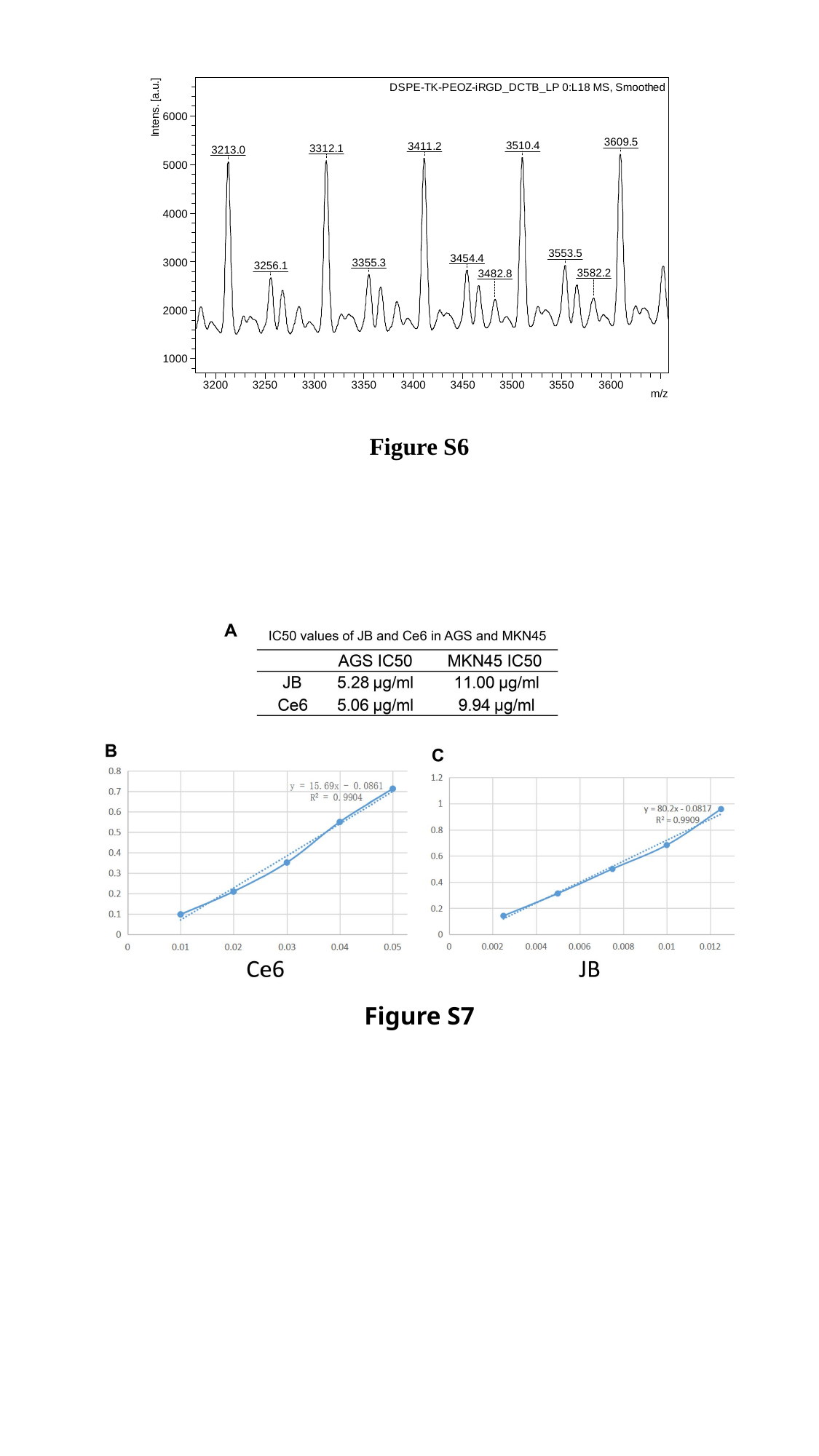

Figure S6
Figure S7

## Slide 6
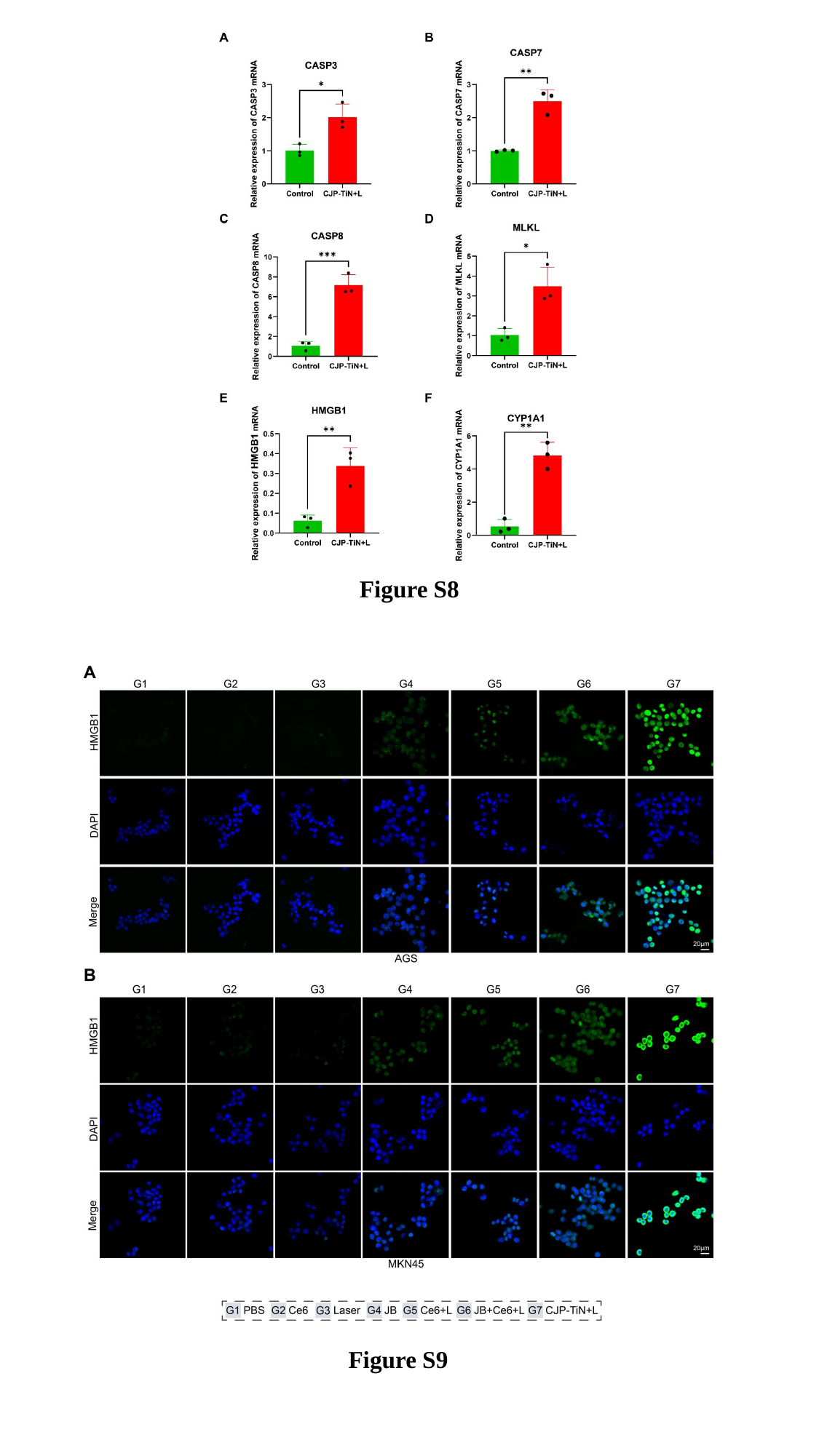

Figure S8
Figure S9

## Slide 7
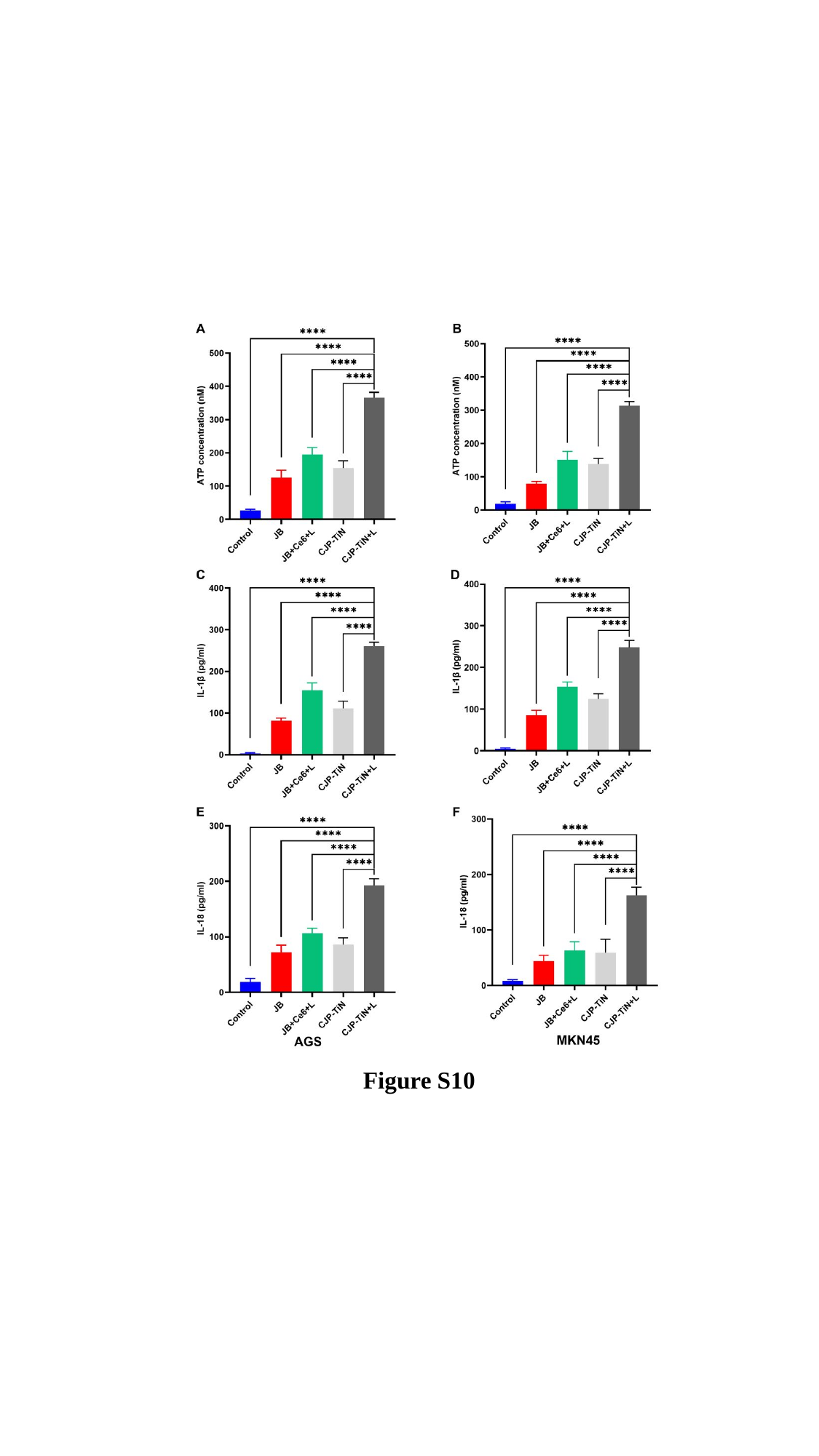

Figure S10

## Slide 8
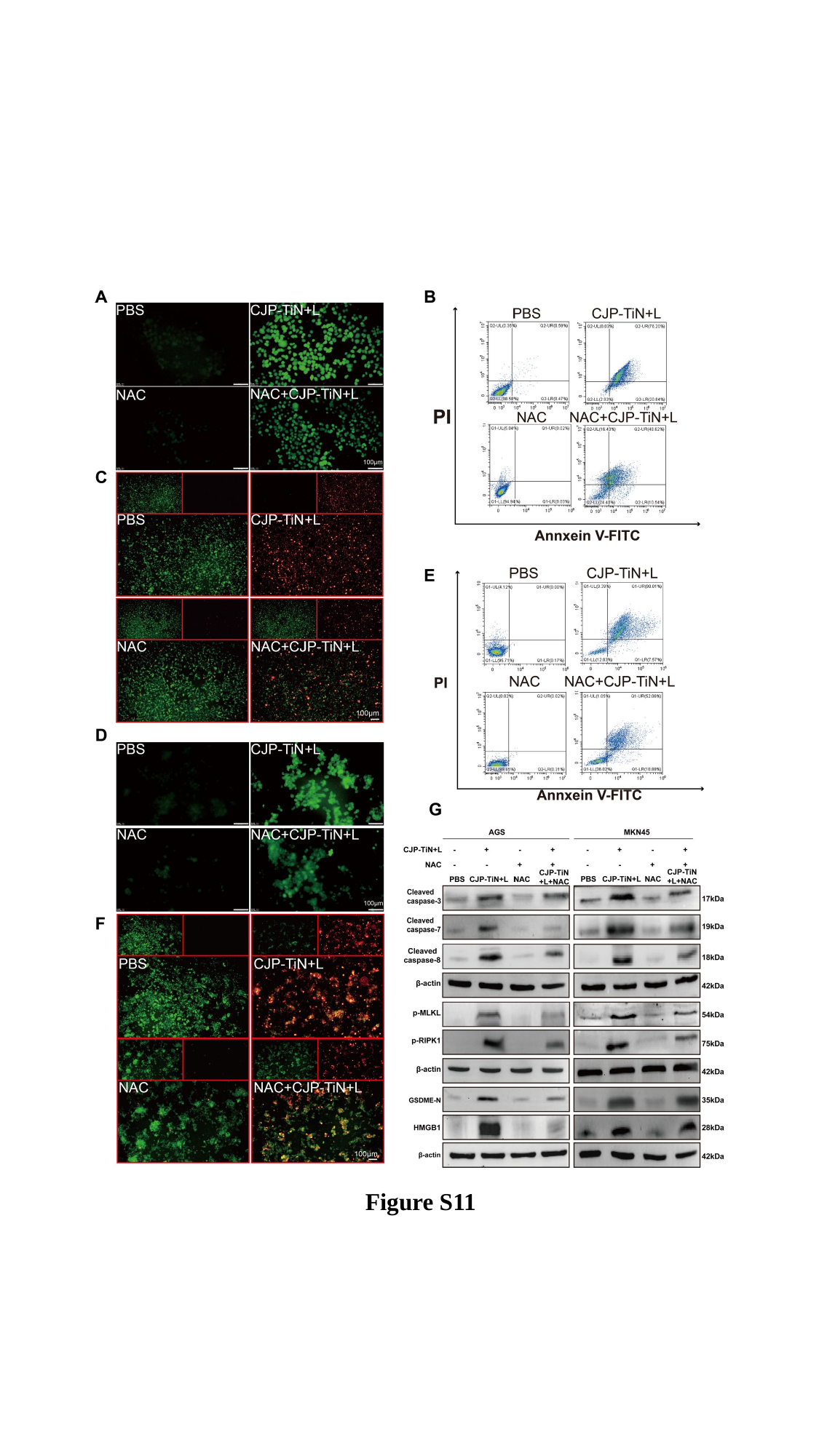

Figure S11

## Slide 9
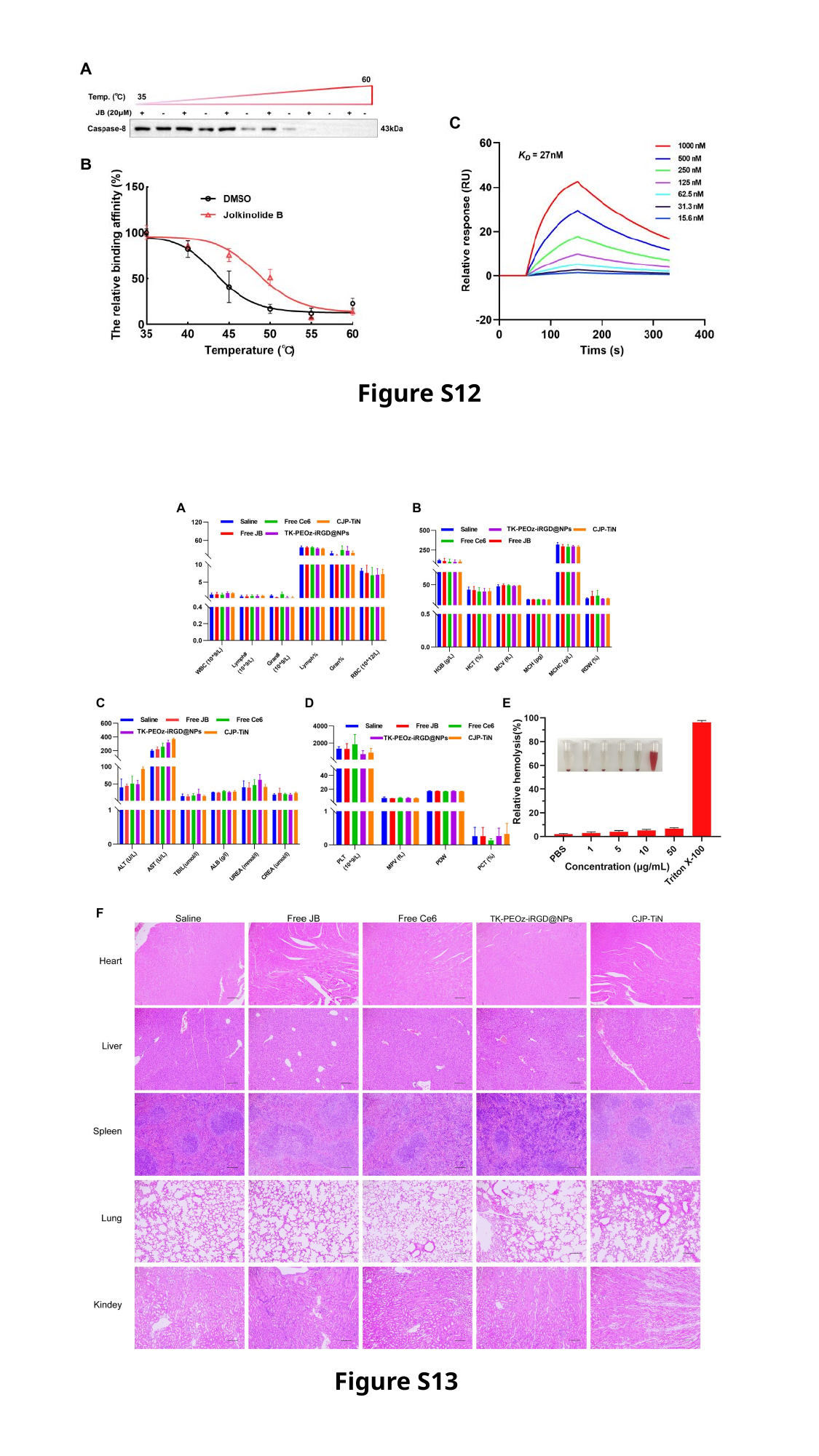

Figure S12
Figure S13

## Slide 10
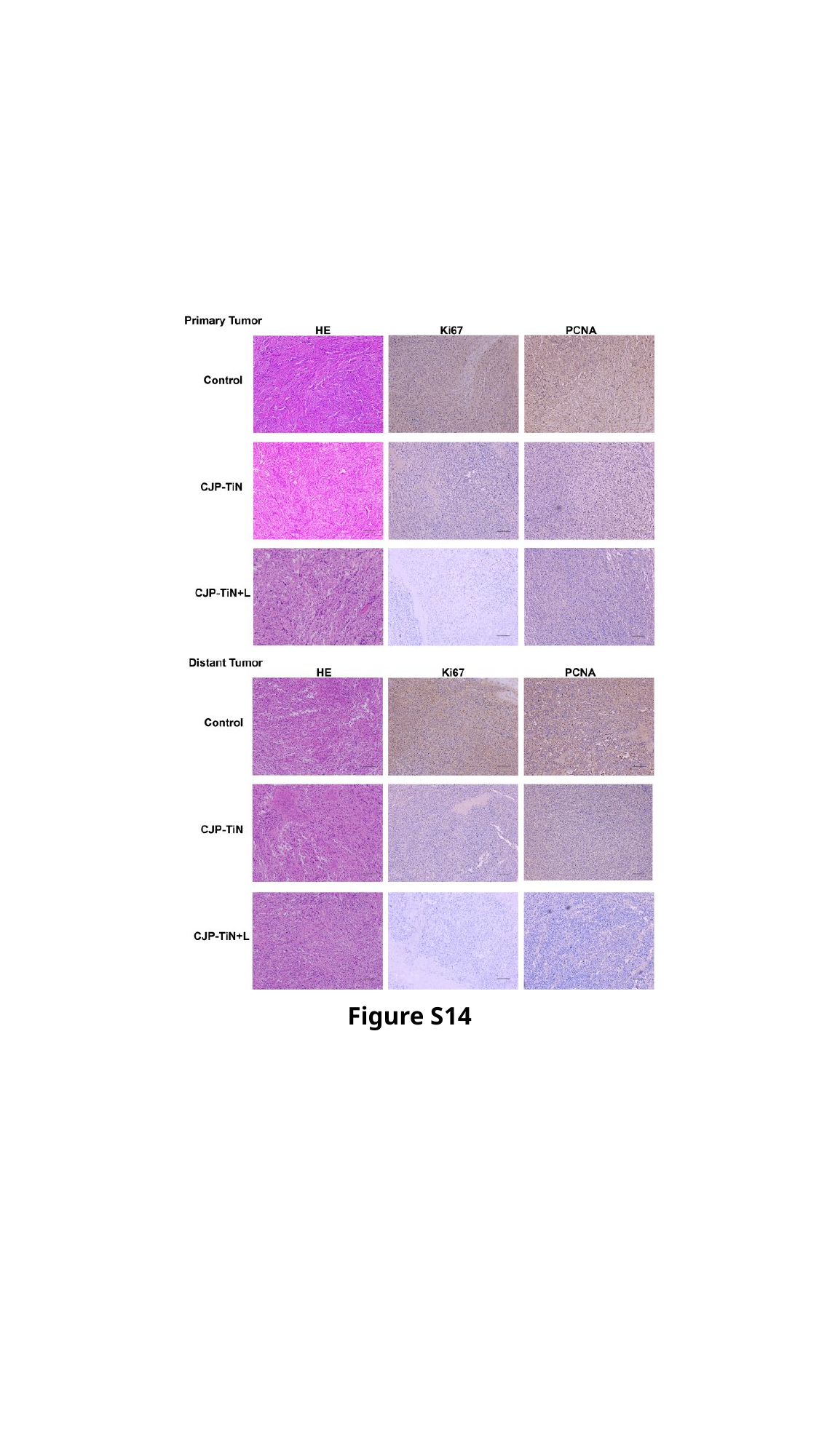

Figure S14

## Slide 11
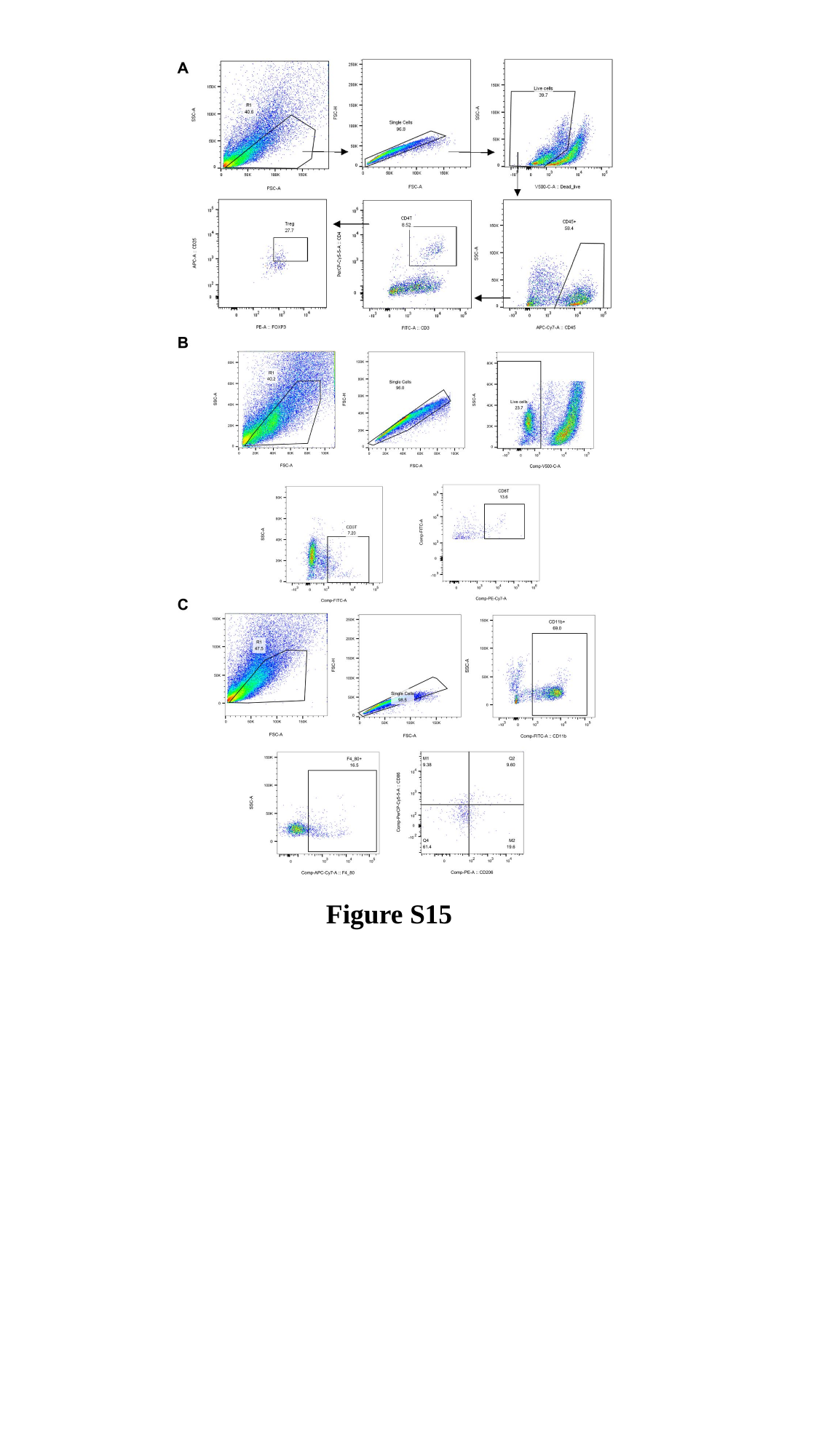

Figure S15

## Slide 12
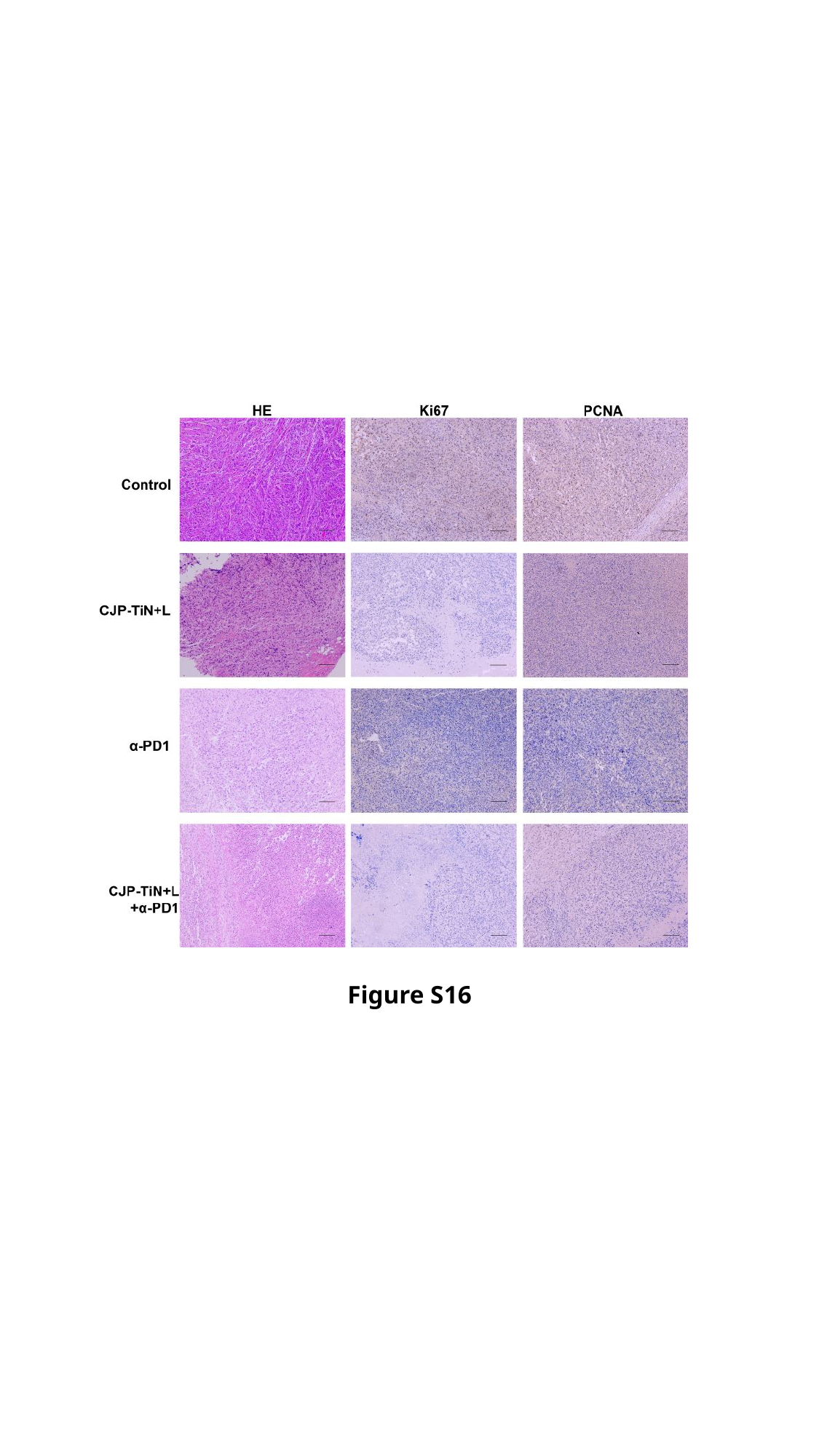

Figure S16
