# Supplementary material for: Targeted Dual‐Responsive Liposomes Co‐Deliver Jolkinolide B and Ce6 to Synergistically Enhance the Photodynamic/Immunotherapy Efficacy in Gastric Cancer through the PANoptosis Pathway
Source: Adv Sci (Weinh). 2025 May 19;12(29):e02289. doi: 10.1002/advs.202502289 (PMC12362734; doi:10.1002/advs.202502289)

ADVANCED SCIENCE
Open Access

Supporting Information

for *Adv. Sci.*, DOI 10.1002/advs.202502289

Targeted Dual-Responsive Liposomes Co-Deliver Jolkinolide B and Ce6 to Synergistically Enhance the Photodynamic/Immunotherapy Efficacy in Gastric Cancer through the PANoptosis Pathway

*Chenhui Ma, Lei Gao, Kewei Song, Baohong Gu, Bofang Wang, Yang Yu, Xueyan Wang, Xuemei Li, Jike Hu, Weigao Pu, Yunpeng Wang, Na Wang, Dedai Lu, Zhijian Han and Hao Chen**

Fig-1F

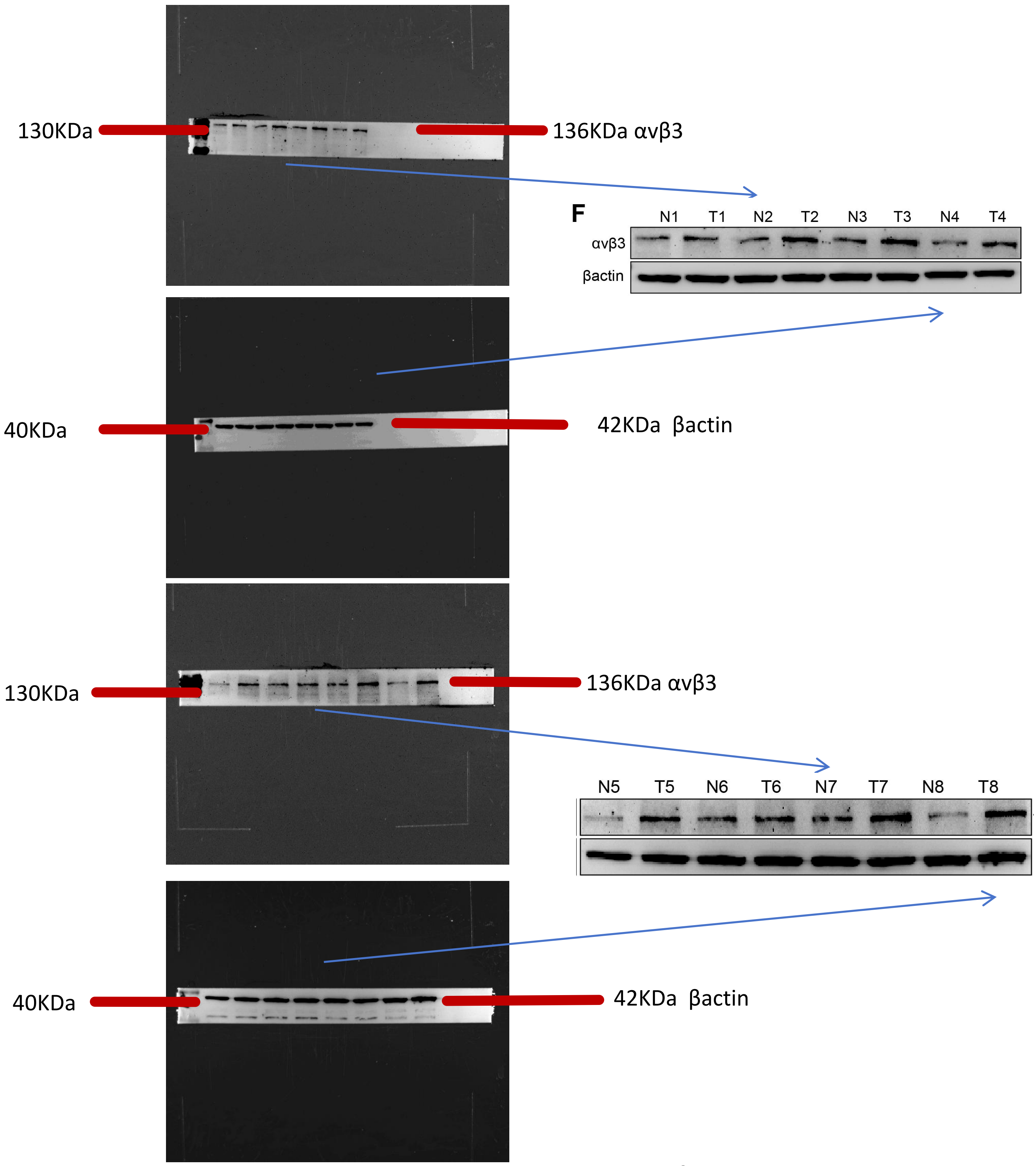
130KDa
136KDa αvβ3
F
N1 T1 N2 T2 N3 T3 N4 T4
αvβ3
βactin
40KDa
42KDa βactin
130KDa
136KDa αvβ3
N5 T5 N6 T6 N7 T7 N8 T8
40KDa
42KDa βactin

Fig-1E

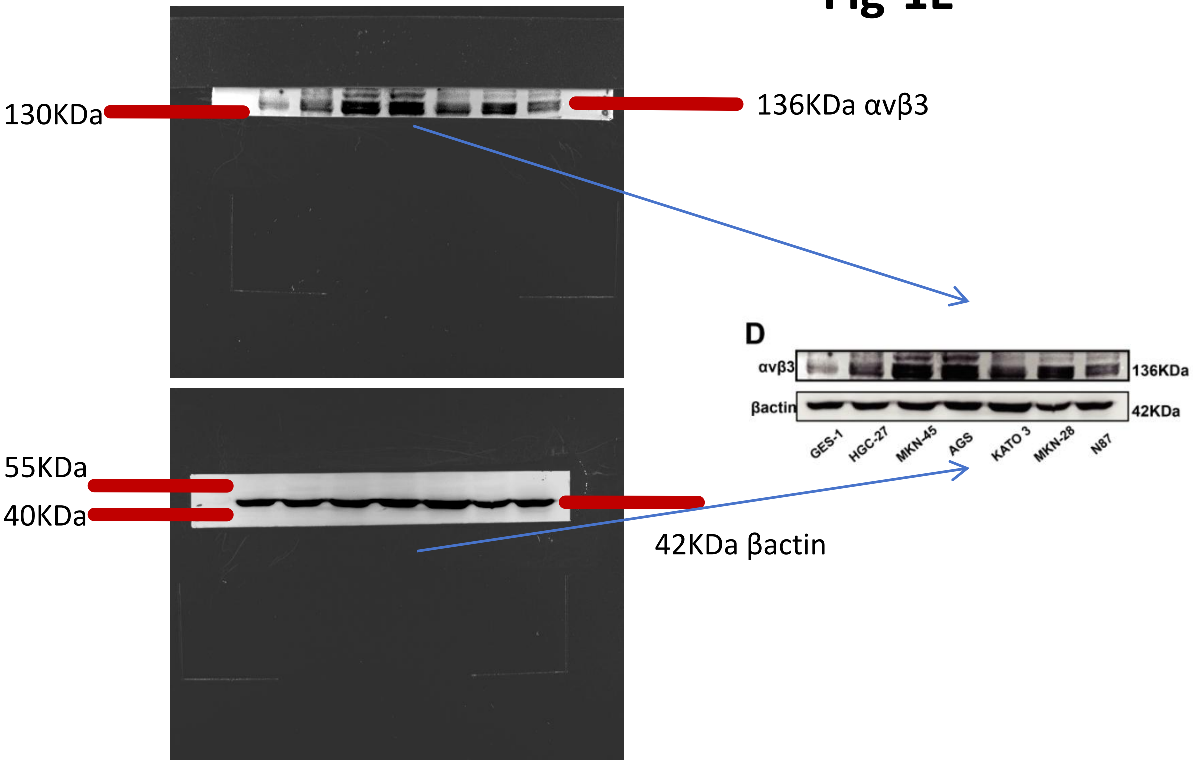
130KDa
136KDa αvβ3
D
αvβ3
136KDa
βactin
42KDa
GES-1
HGC-27
MKN-45
AGS
KATO 3
MKN-28
N87
55KDa
40KDa
42KDa βactin

Fig-4F-AGS

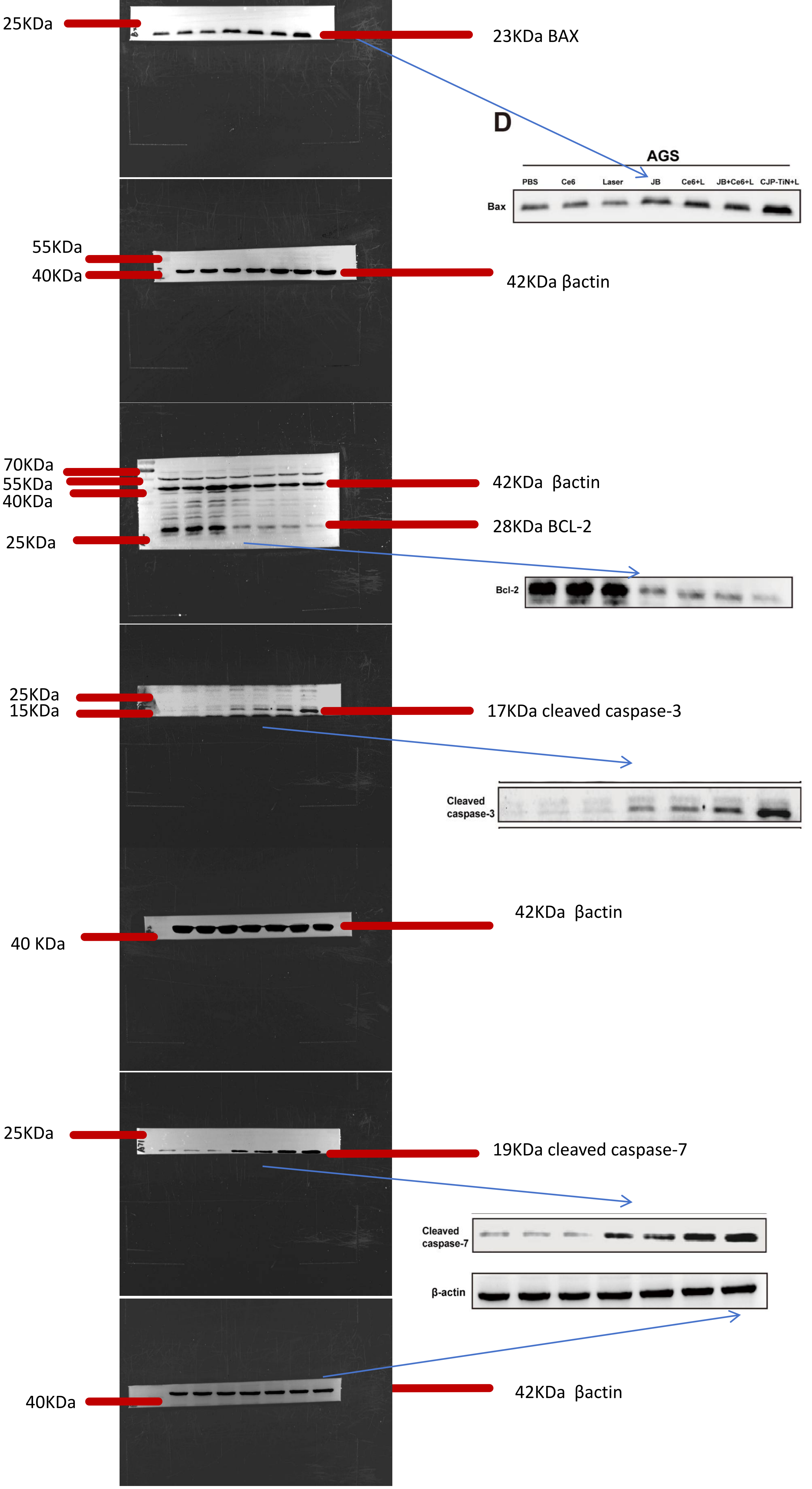
25KDa
23KDa BAX
D
AGS
PBS
Ce6
Laser
JB
Ce6+L
JB+Ce6+L
CJP-TiN+L
Bax
55KDa
40KDa
42KDa βactin
70KDa
55KDa
40KDa
42KDa βactin
25KDa
28KDa BCL-2
Bcl-2
25KDa
15KDa
17KDa cleaved caspase-3
Cleaved caspase-3
42KDa βactin
40 KDa
25KDa
19KDa cleaved caspase-7
Cleaved caspase-7
β-actin
40KDa
42KDa βactin

Fig-4F-AGS

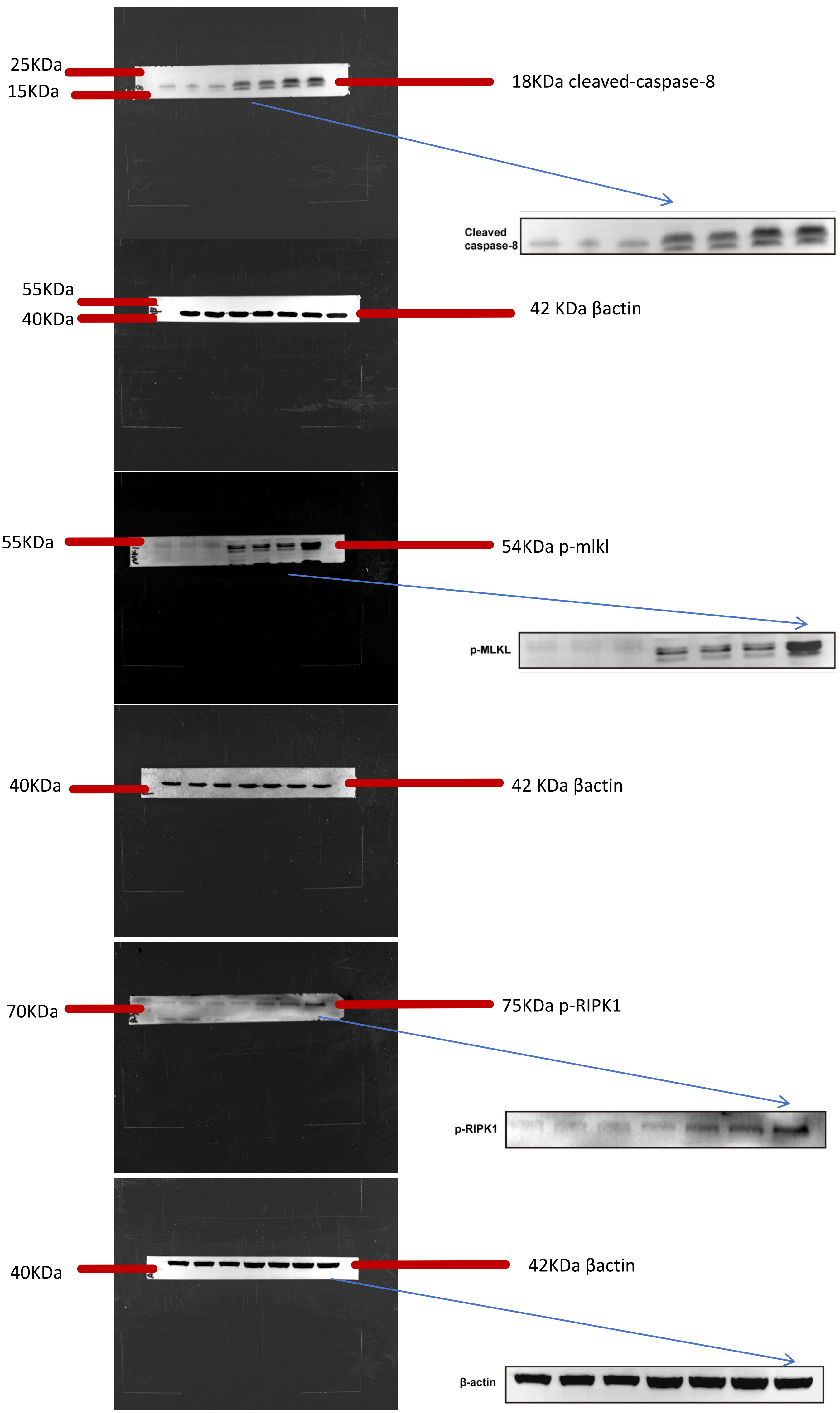
25KDa
15KDa
18KDa cleaved-caspase-8
Cleaved caspase-8
55KDa
40KDa
42 KDa βactin
55KDa
54KDa p-mlkl
p-MLKL
40KDa
42 KDa βactin
70KDa
75KDa p-RIPK1
p-RIPK1
40KDa
42KDa βactin
β-actin

Fig-4F-AGS

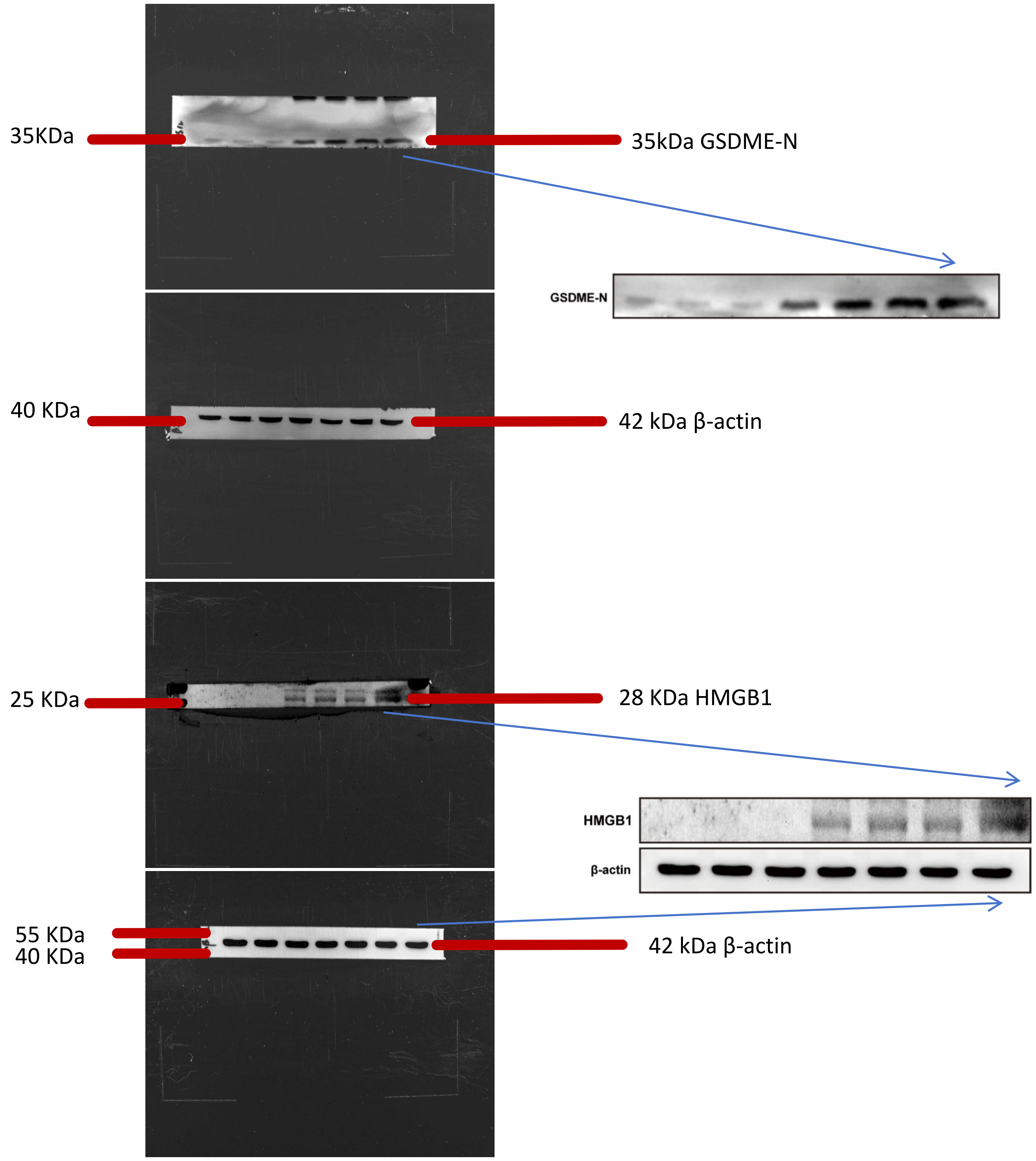

Fig-4F-MKN45

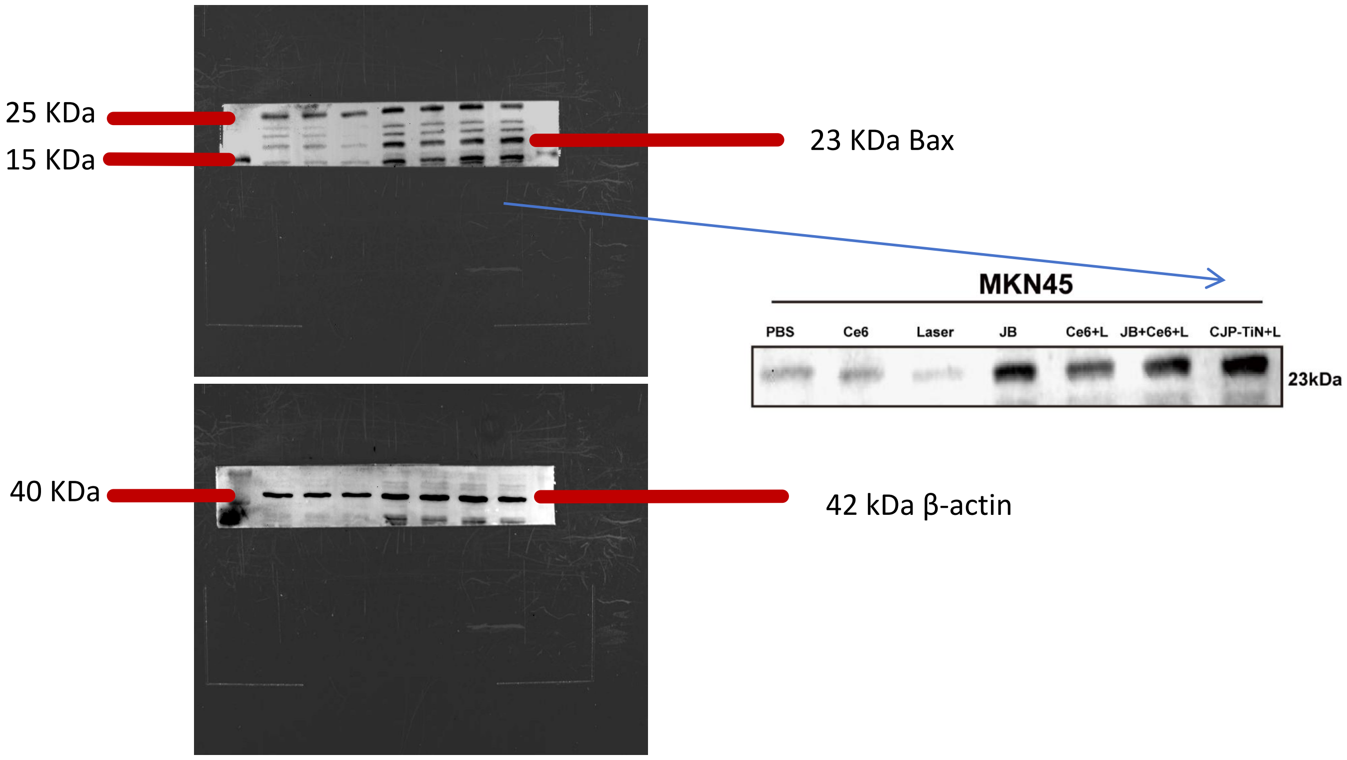

Fig-4F-MKN45

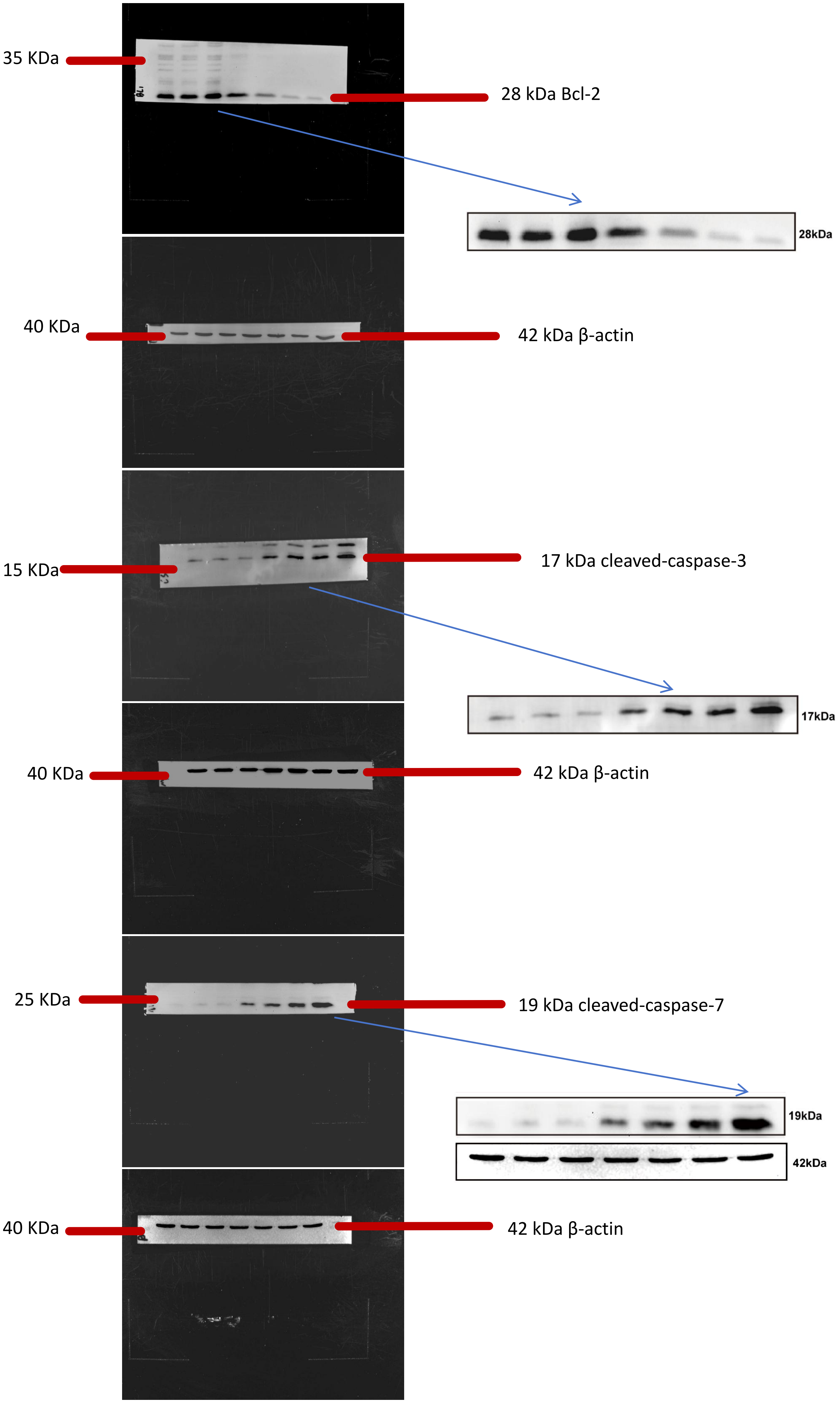
35 KDa
28 kDa Bcl-2
28kDa
40 KDa
42 kDa β-actin
15 KDa
17 kDa cleaved-caspase-3
17kDa
40 KDa
42 kDa β-actin
25 KDa
19 kDa cleaved-caspase-7
19kDa
42kDa
40 KDa
42 kDa β-actin

Fig-4F-MKN45

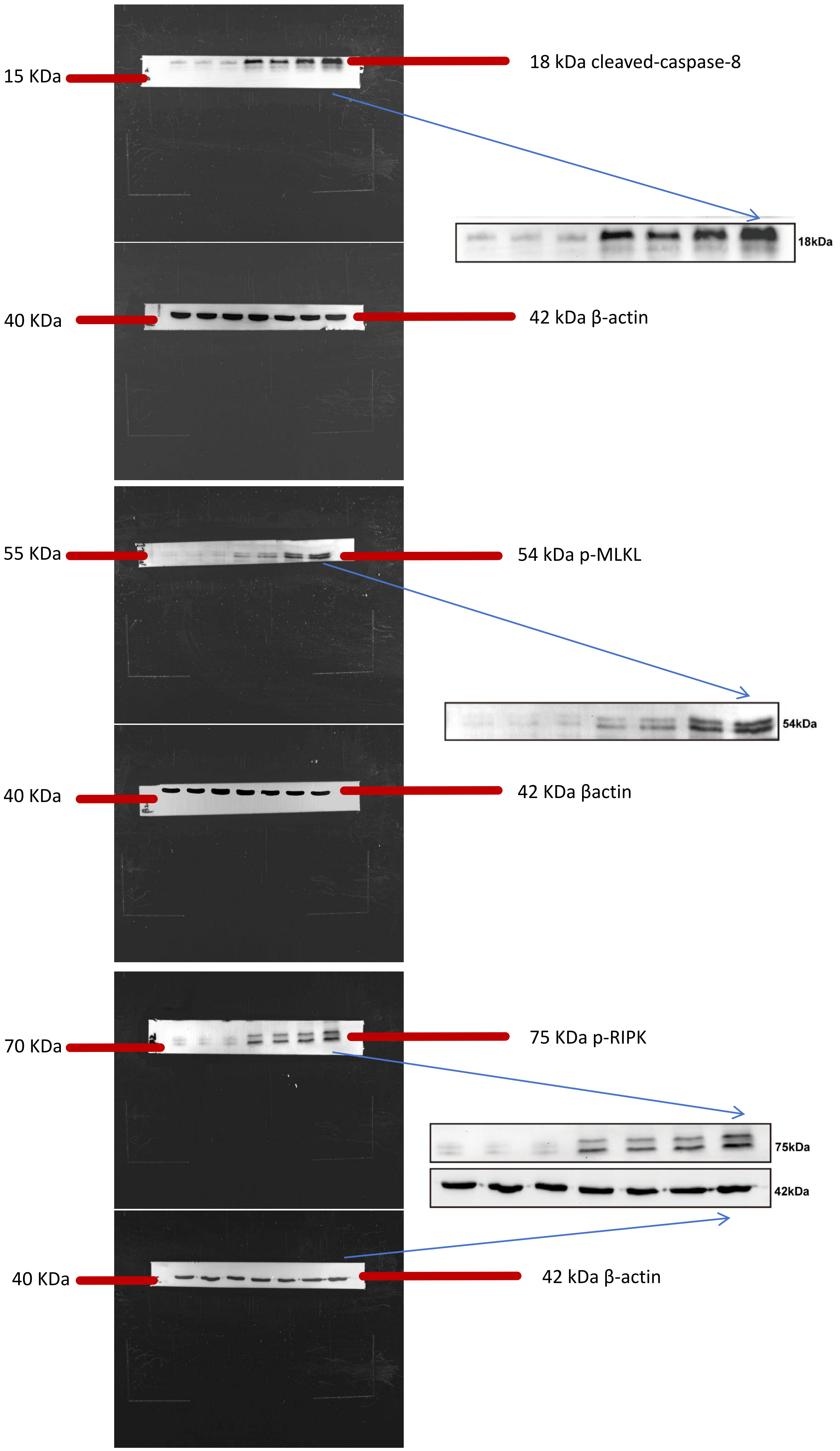
15 KDa
18 kDa cleaved-caspase-8
18kDa
40 KDa
42 kDa β-actin
55 KDa
54 kDa p-MLKL
54kDa
40 KDa
42 KDa βactin
70 KDa
75 KDa p-RIPK
75kDa
42kDa
40 KDa
42 kDa β-actin

Fig-4F-MKN45

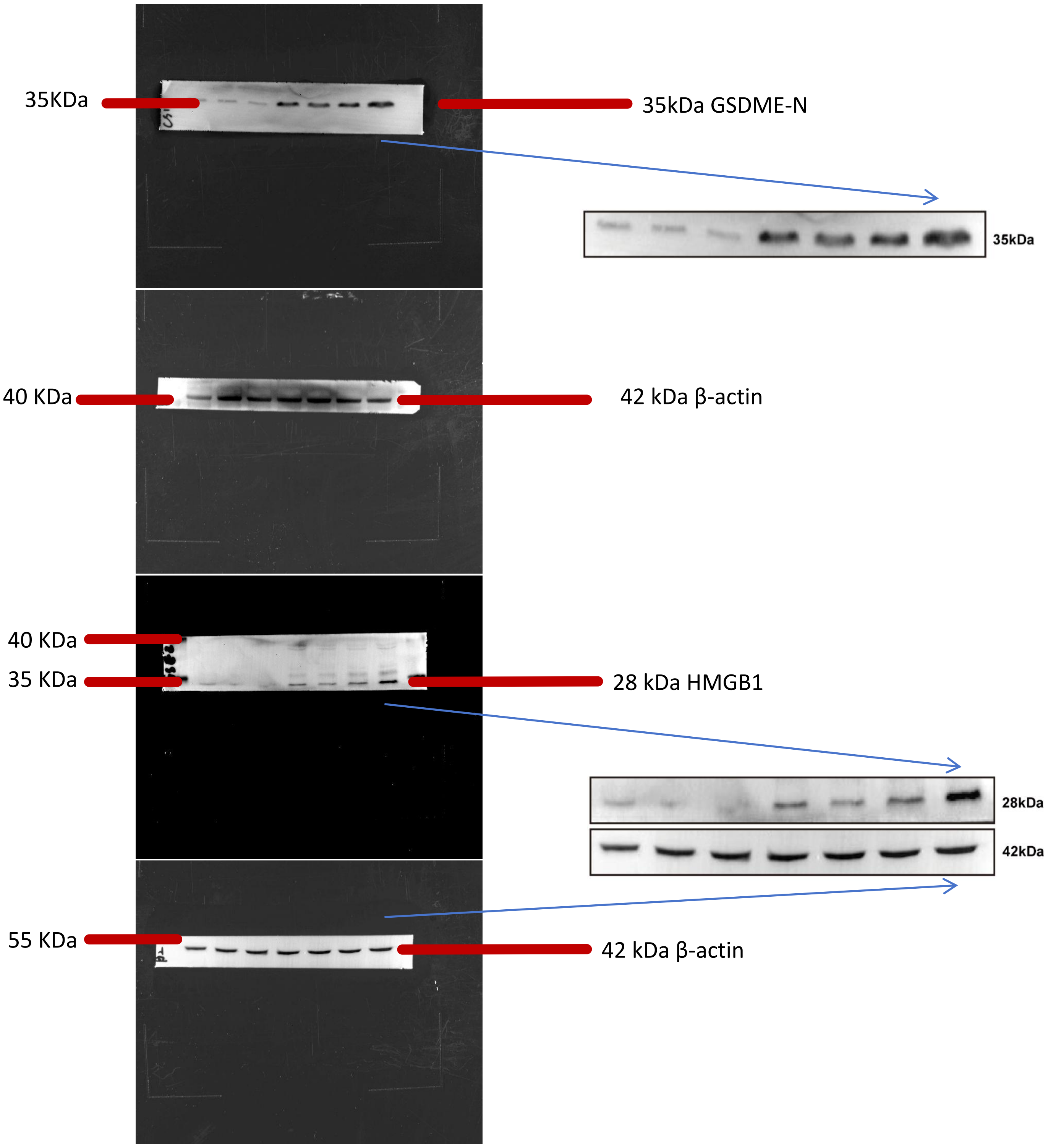
35KDa
35kDa GSDME-N
35kDa
40 KDa
42 kDa β-actin
40 KDa
35 KDa
28 kDa HMGB1
28kDa
42kDa
55 KDa
42 kDa β-actin

Fig-S8G-AGS

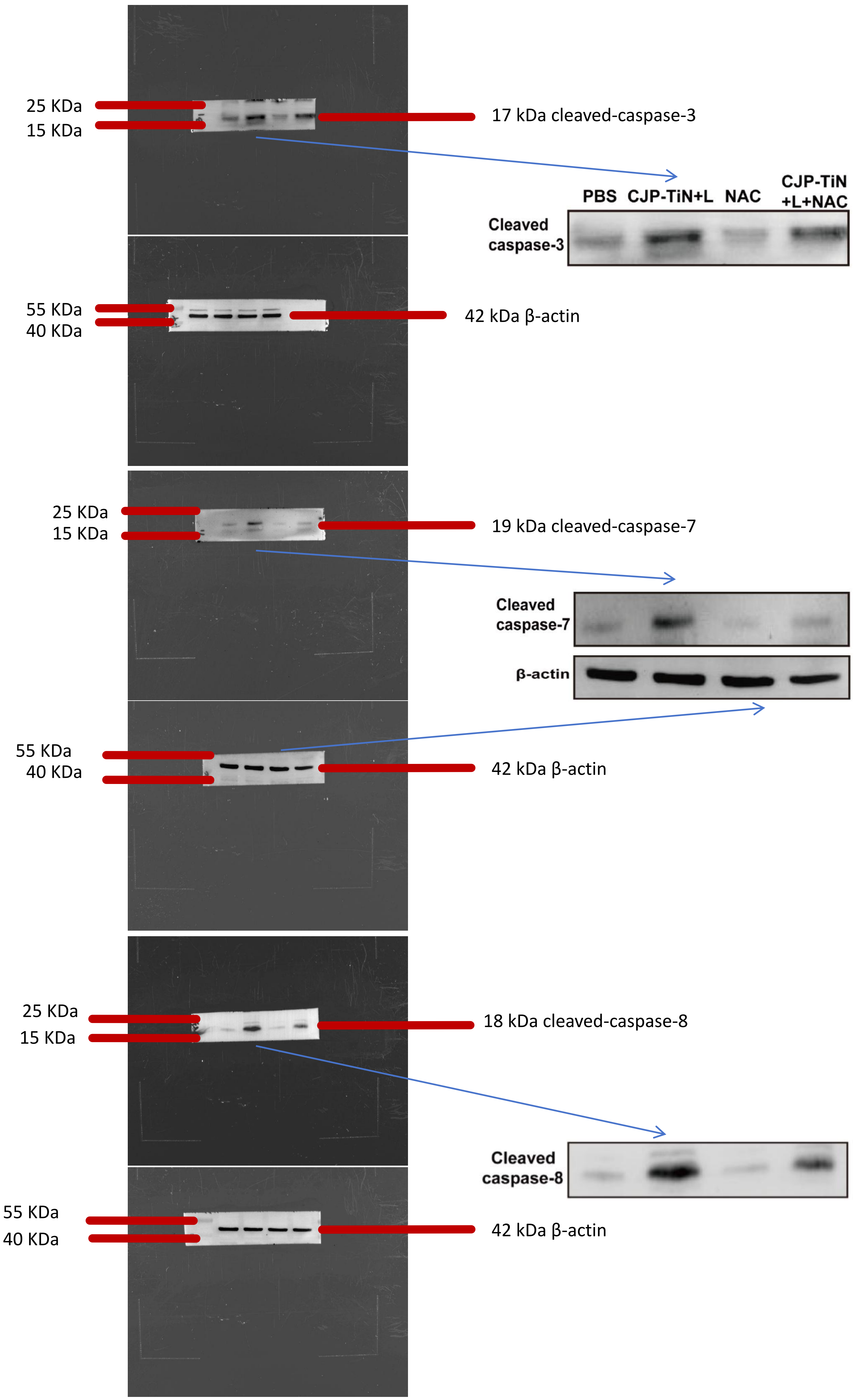
25 KDa
15 KDa
17 kDa cleaved-caspase-3
PBS
CJP-TiN+L
NAC
CJP-TiN +L+NAC
Cleaved caspase-3
55 KDa
40 KDa
42 kDa β-actin
25 KDa
15 KDa
19 kDa cleaved-caspase-7
Cleaved caspase-7
β-actin
55 KDa
40 KDa
42 kDa β-actin
25 KDa
15 KDa
18 kDa cleaved-caspase-8
Cleaved caspase-8
55 KDa
40 KDa
42 kDa β-actin

Fig-S8G-AGS

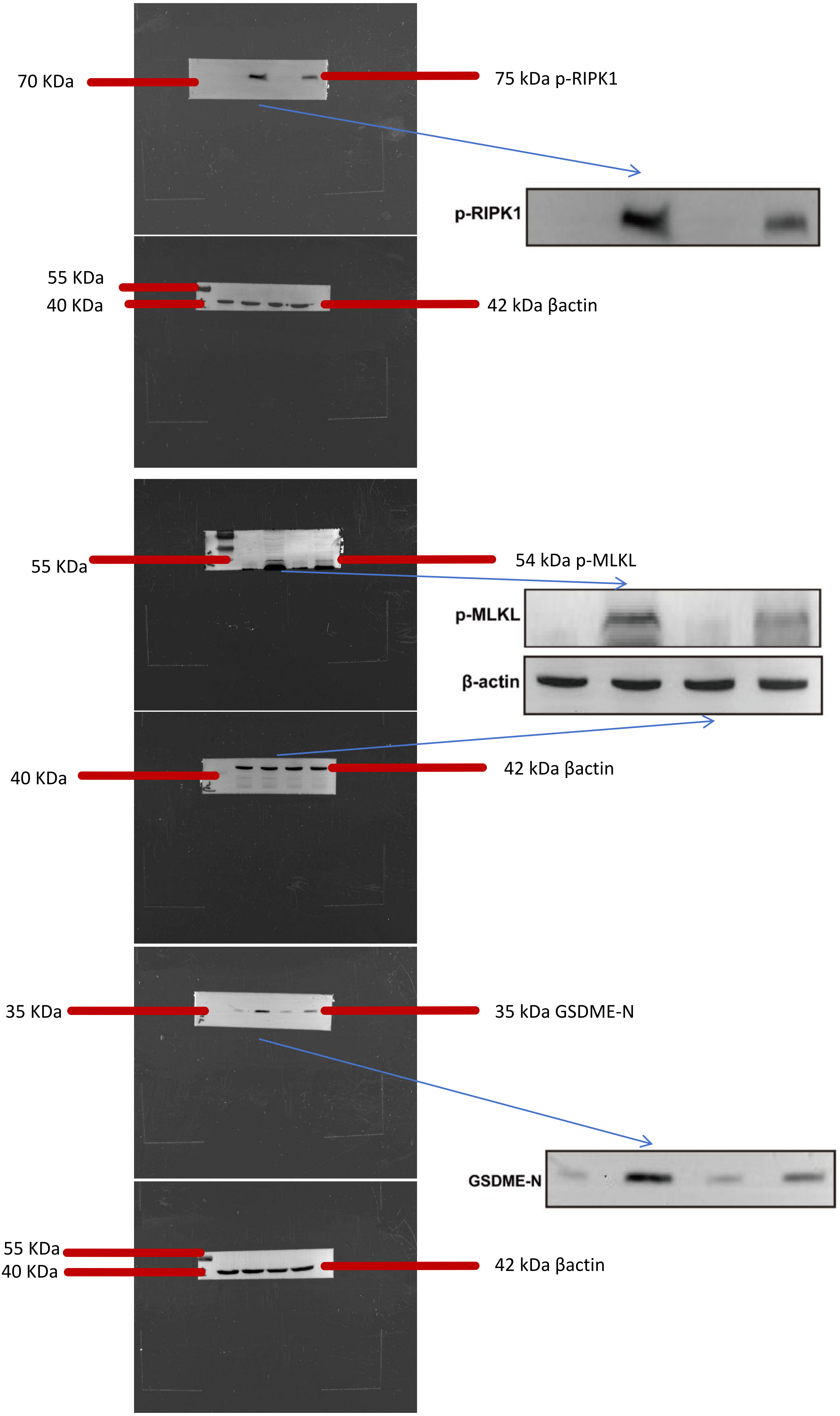
70 KDa
75 kDa p-RIPK1
p-RIPK1
55 KDa
40 KDa
42 kDa βactin
55 KDa
54 kDa p-MLKL
p-MLKL
β-actin
40 KDa
42 kDa βactin
35 KDa
35 kDa GSDME-N
GSDME-N
55 KDa
40 KDa
42 kDa βactin

Fig-S8G-AGS

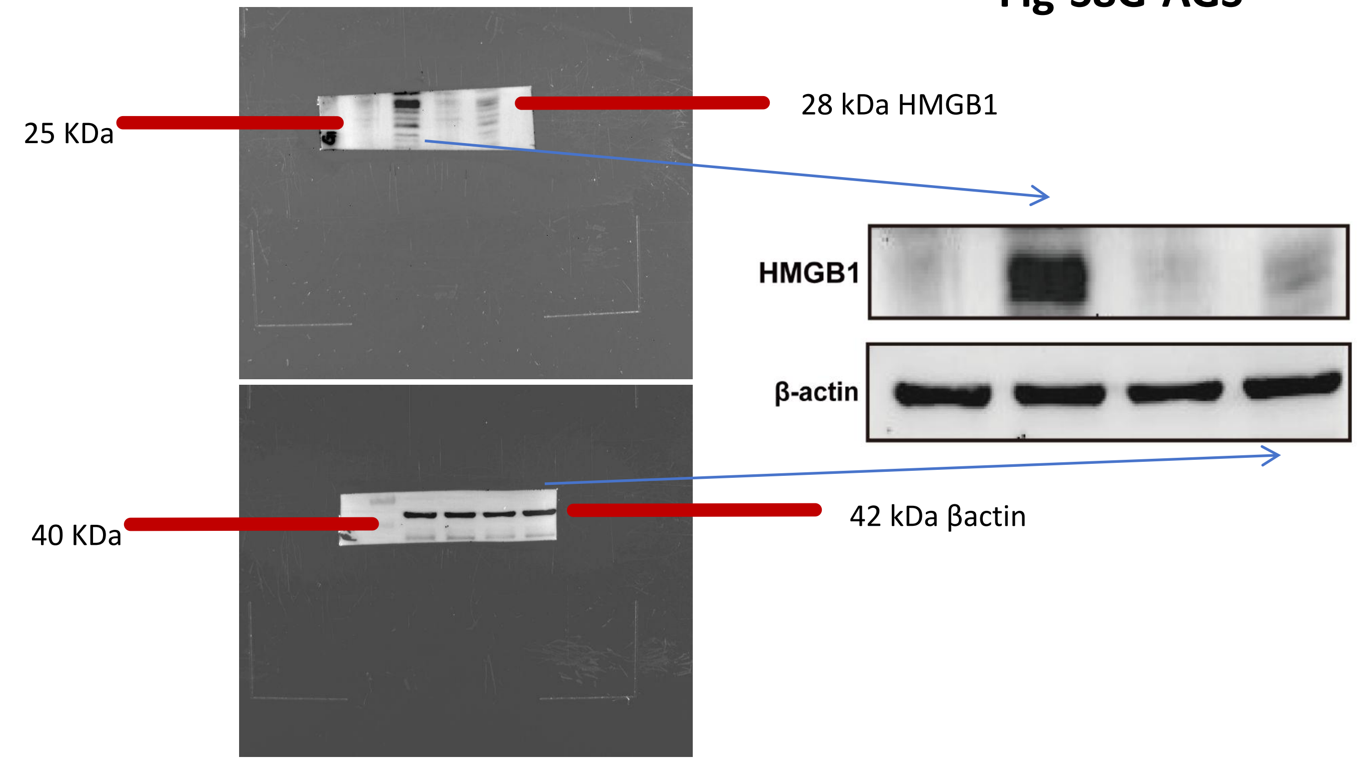

Fig-S8G-MKN45

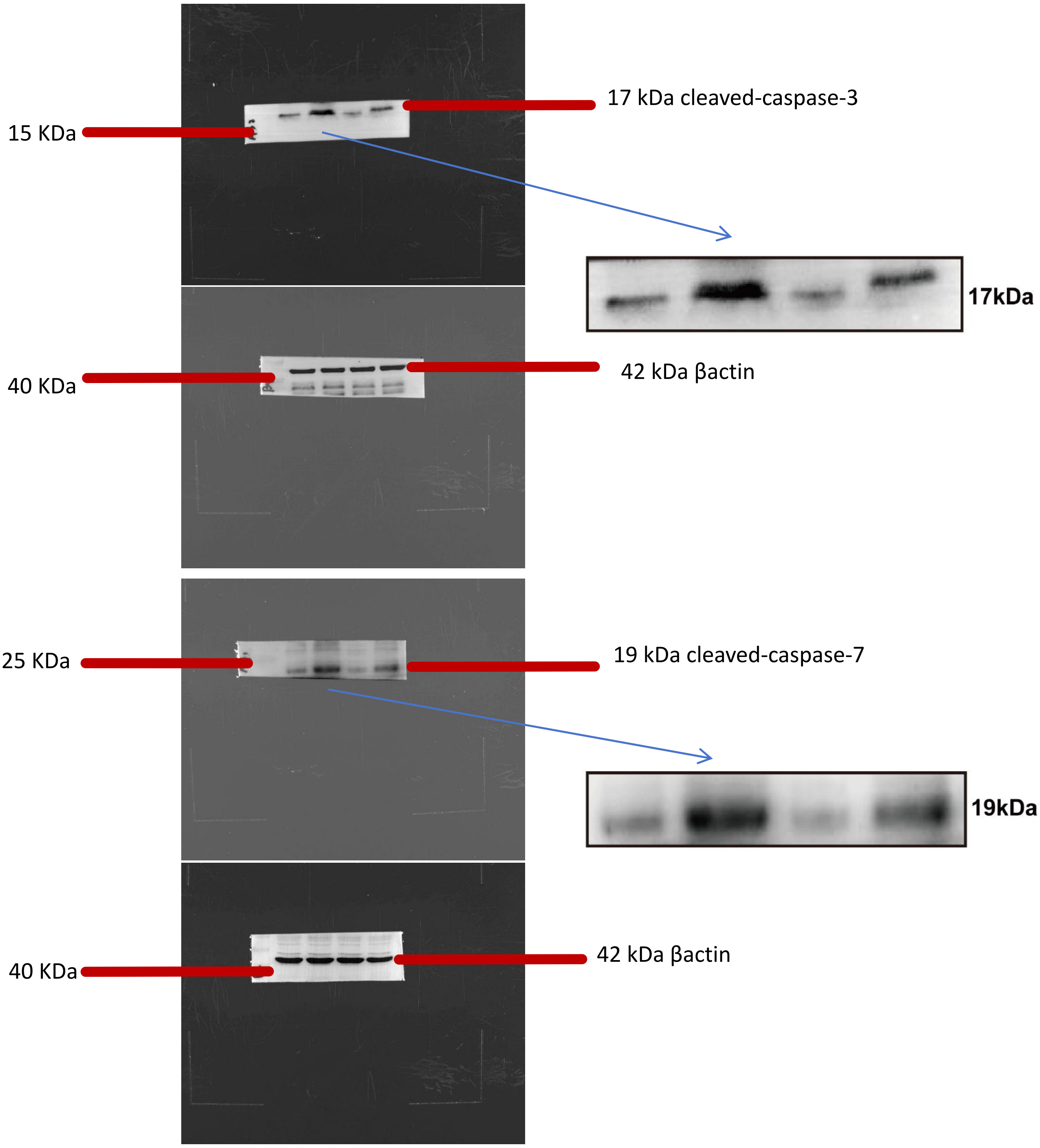

Fig-S8G-MKN45

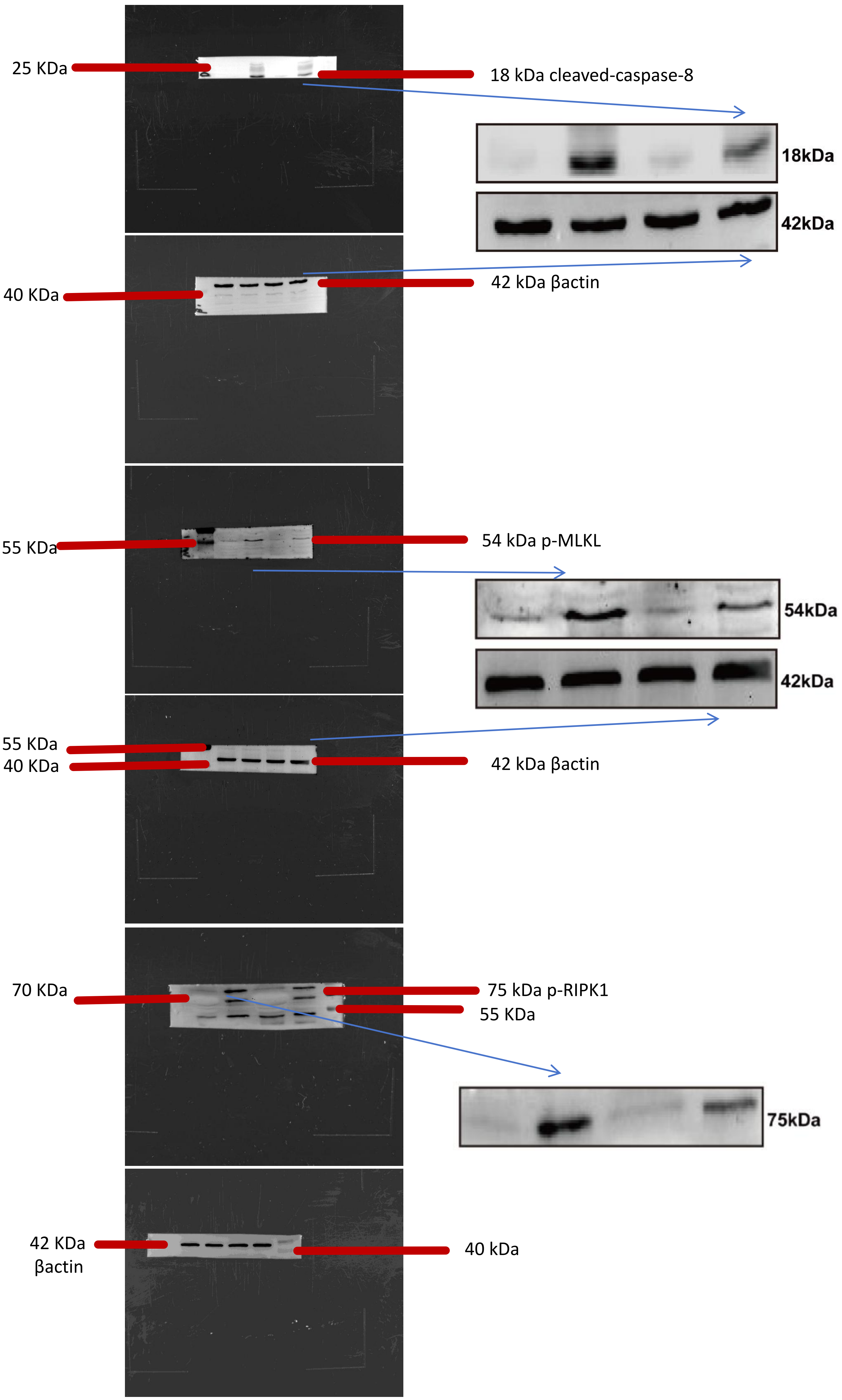

Fig-S8G-MKN45

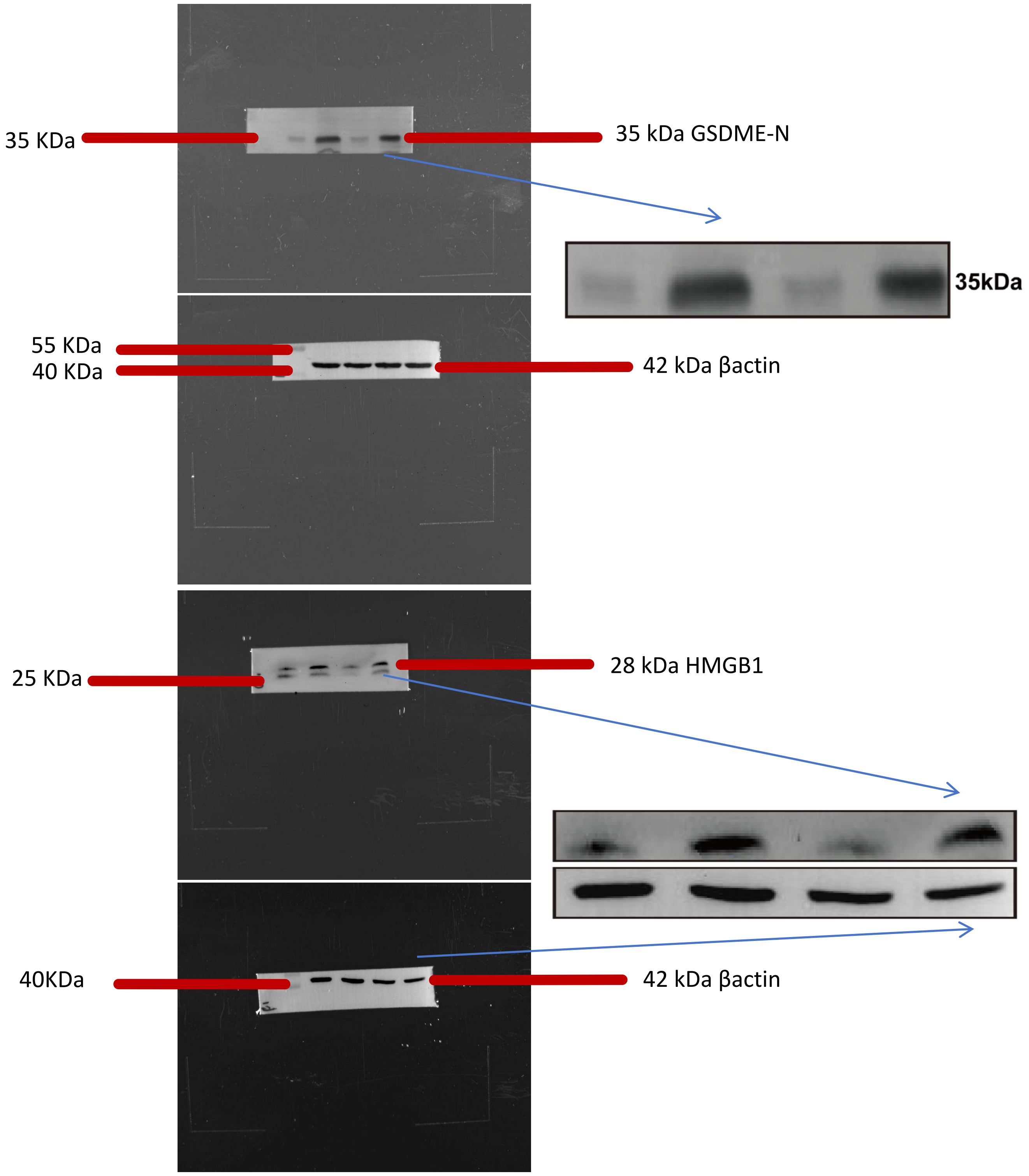

Supplement: Supplementary file 3 — Supporting Information [file ADVS-12-e02289-s003.pdf]
